# Bi-allelic *CAMSAP1* variants cause a clinically recognizable neuronal migration disorder

**DOI:** 10.1016/j.ajhg.2022.09.012

**Published:** 2022-10-24

**Authors:** Reham Khalaf-Nazzal, James Fasham, Katherine A. Inskeep, Lauren E. Blizzard, Joseph S. Leslie, Matthew N. Wakeling, Nishanka Ubeyratna, Tadahiro Mitani, Jennifer L. Griffith, Wisam Baker, Fida’ Al-Hijawi, Karen C. Keough, Alper Gezdirici, Loren Pena, Christine G. Spaeth, Peter D. Turnpenny, Joseph R. Walsh, Randall Ray, Amber Neilson, Evguenia Kouranova, Xiaoxia Cui, David T. Curiel, Davut Pehlivan, Zeynep Coban Akdemir, Jennifer E. Posey, James R. Lupski, William B. Dobyns, Rolf W. Stottmann, Andrew H. Crosby, Emma L. Baple

**Affiliations:** 1 Biomedical Sciences Department, Faculty of Medicine, Arab American University of Palestine, Jenin P227, Palestine; 2 Department of Clinical and Biomedical Science, University of Exeter Faculty of Health and Life Science, RILD building, Barrack Road, Exeter EX2 5DW, UK; 3 Peninsula Clinical Genetics Service, Royal Devon University Healthcare NHS Foundation Trust (Heavitree Hospital), Gladstone Road, Exeter EX1 2ED, UK; 4 Division of Developmental Biology, Cincinnati Children’s Hospital Medical Center, 3333 Burnet Avenue, MLC 7016, Cincinnati, OH 45229, USA; 5 Institute for Genomic Medicine at Nationwide Children’s Hospital, The Ohio State University College of Medicine, Columbus, OH 43205, USA; 6 Division of Human Genetics, Cincinnati Children’s Hospital Medical Center, 3333 Burnet Avenue, MLC 7016, Cincinnati, OH 45229, USA; 7 Department of Pediatrics, University of Cincinnati College of Medicine, Cincinnati, OH 45229, USA; 8 Department of Molecular and Human Genetics, Baylor College of Medicine, Houston, TX 77030, USA; 9 Department of Neurology, Washington University School of Medicine, St. Louis, MO 63110, USA; 10 Paediatrics Department, Dr. Khalil Suleiman Government Hospital, Jenin, Palestine; 11 Paediatrics Community Outpatient Clinics, Palestinian Ministry of Health, Jenin, Palestine; 12 Department of Pediatrics, Dell Medical School, 1400 Barbara Jordan Boulevard, Austin, TX 78723, USA; 13 Child Neurology Consultants of Austin, 7940 Shoal Creek Boulevard, Suite 100, Austin, TX 78757, USA; 14 Department of Medical Genetics, Başakşehir Çam and Sakura City Hospital, 34480 Istanbul, Turkey; 15 Department of Neurological Surgery, School of Medicine, Washington University in Saint Louis, St. Louis, MO 63110, USA; 16 Departments of Pediatrics and Medical Genetics, Boston Children’s Hospital and Harvard Medical School, Boston, MA 02115, USA; 17 Genome Engineering & Stem Cell Center, Department of Genetics, School of Medicine, Washington University in Saint Louis, St. Louis, MO 63110, USA; 18 Department of Biomedical Engineering, McKelvey School of Engineering, Washington University in Saint Louis, St. Louis, MO 63130, USA; 19 Division of Cancer Biology, Department of Radiation Oncology, School of Medicine, Washington University in Saint Louis, St. Louis, MO 63110, USA; 20 Biologic Therapeutics Center, Department of Radiation Oncology, School of Medicine, Washington University in Saint Louis, St. Louis, MO 63110, USA; 21 Division of Neurology and Developmental Neuroscience, Department of Pediatrics, Baylor College of Medicine, Houston, TX 77030, USA; 22 Human Genome Sequencing Center, Baylor College of Medicine, Houston, TX 77030, USA; 23 Department of Pediatrics, Baylor College of Medicine, Houston, TX 77030, USA; 24 Texas Children’s Hospital, Houston, TX 77030, USA; 25 Departments of Pediatrics and Genetics, University of Minnesota, Minneapolis, MN, USA

**Keywords:** neurodevelopmental disorder, tubulinopathy, lissencephaly, pachygyria, agyria, autosomal recessive, MARK2, patronin

## Abstract

Non-centrosomal microtubules are essential cytoskeletal filaments that are important for neurite formation, axonal transport, and neuronal migration. They require stabilization by microtubule minus-end-targeting proteins including the CAMSAP family of molecules. Using exome sequencing on samples from five unrelated families, we show that bi-allelic *CAMSAP1* loss-of-function variants cause a clinically recognizable, syndromic neuronal migration disorder. The cardinal clinical features of the syndrome include a characteristic craniofacial appearance, primary microcephaly, severe neurodevelopmental delay, cortical visual impairment, and seizures. The neuroradiological phenotype comprises a highly recognizable combination of classic lissencephaly with a posterior more severe than anterior gradient similar to *PAFAH1B1(LIS1)-*related lissencephaly and severe hypoplasia or absence of the corpus callosum; dysplasia of the basal ganglia, hippocampus, and midbrain; and cerebellar hypodysplasia, similar to the tubulinopathies, a group of monogenic tubulin-associated disorders of cortical dysgenesis. Neural cell rosette lineages derived from affected individuals displayed findings consistent with these phenotypes, including abnormal morphology, decreased cell proliferation, and neuronal differentiation. *Camsap1*-null mice displayed increased perinatal mortality, and RNAScope studies identified high expression levels in the brain throughout neurogenesis and in facial structures, consistent with the mouse and human neurodevelopmental and craniofacial phenotypes. Together our findings confirm a fundamental role of CAMSAP1 in neuronal migration and brain development and define bi-allelic variants as a cause of a clinically distinct neurodevelopmental disorder in humans and mice.

## Teaser

We describe bi-allelic variants in *CAMSAP1*, which encodes a molecule crucially important for minus-end microtubule stabilization, as a cause of a clinically recognizable, syndromic neuronal migration disorder with similarities to the “tubulinopathies.” *Camsap1*^−/−^ mice displayed increased perinatal mortality, and proband-derived neural cell rosette lineages showed decreased cell proliferation and differentiation.

## Main text

Neuronal migration disorders arise from defects in the locomotion of neurons in the prenatal developing brain, resulting in early onset developmental impairment and seizures.^1^^,^^2^ These conditions are characterized by neuroradiological and histopathological abnormalities of cortical layering, absence of normal folding (lissencephaly), agenesis of the corpus callosum, and hypo/dysgenesis of the cerebellum. A number of monogenic causes have been described, almost all of which impact the formation or functioning of microtubules.^3^^,^^4^ Owing to the severity of the neurological phenotype, most cases are sporadic, resulting from *de novo* heterozygous loss-of-function variants. In published cohorts, heterozygous pathogenic variants in *PAFAH1B1* (MIM: 601545) (platelet-activating factor acetylhydrolase, isoform 1b, alpha subunit, formerly *LIS1*), which interacts with the microtubule motor cytoplasmic dynein,^5^ account for more than a third of affected individuals,^6^ and hemizygous or heterozygous pathogenic variants in doublecortin (*DCX*), important for microtubule stabilization and inhibition of neurite outgrowth,^7^ a further quarter.^6^ Variants in several other microtubule-interacting molecules including dynein, cytoplasmic 1, heavy chain 1 (*DYNC1H1*) as well as the alpha (*TUBA1A*) (MIM: 611603), beta (*TUBB2A*, *TUBB2B*, *TUBB3*, *TUBB4A*, *TUBB*) (MIM: 615763, 610031, 614039, 612438, 615771), and gamma-tubulin (*TUBG1*) (MIM: 615412) subunits are also well-recognized genetic causes of neuronal migration and brain malformation disorders.^4^^,^^8^ Those conditions caused by pathogenic variants in tubulin subunits are collectively termed the “tubulinopathies”^9^ and display distinctive neuroradiological features, which in addition to the cortical layering abnormalities, include hypoplasia/aplasia of the corpus callosum, hypoplasia of the oculomotor and optic nerves, cerebellar hypodysplasia (including foliar dysplasia), and dysmorphism of the basal ganglia and hind-brain structures.^10^

The CAMSAP (calmodulin-regulated spectrin-associated protein) family contains three human proteins (CAMSAP1, CAMSAP2 [CAMSAP1L1/KIAA1078] and CAMSAP3 [KIAA1543]) essential for the formation and maintenance of the minus end of non-centrosomal microtubules.^11^ At least one orthologue exists in all eumetazoans, including the well-described *Patronin* in *Drosophila*, indicating a fundamental requirement for at least one CAMSAP molecule in mitotic processes.^12^^,^^13^ CAMSAP proteins are defined by a highly conserved “CKK” (CAMSAP1, KIAA1078/CAMSAP2, KIAA1543/CAMSAP3) domain at the C terminus^12^ with an additional 5′ calponin homology (CH) domain and intermediate coiled-coil (CC) motif also invariably present.^14^ The CKK domain, predicted to adopt a β-barrel conformation with a single invariant tryptophan residue within its core, directs each CAMSAP protein to the microtubule minus end. CAMSAP1 binds transiently to the outermost microtubule ends,^15^ stabilizing them without affecting tubulin incorporation rate *in vitro*.^11^^,^^16^^,^^17^ Conversely, CAMSAP2 and CAMSAP3 remain bound to and decorate the microtubule lattice, exerting a stabilizing effect and reducing tubulin incorporation rates.^11^^,^^17^^,^^18^ A recently described *Camsap1* knockout mouse model (*Camsap1*^−/−^) manifested preweaning lethality with epileptic seizures and abnormal cortical lamination, highly suggestive of impaired neuronal migration.^19^ Neurons from *Camsap1*^−/−^ mice displayed abnormal polarization resulting in a multi-axon phenotype. To date however, no human monogenic disorder has been associated with any of the *CAMSAP* genes. Here, we define bi-allelic *CAMSAP1* (MIM: 613774) variants as a cause of a clinically and radiologically distinct syndromic neuronal migration disorder.

**Figure 1:**
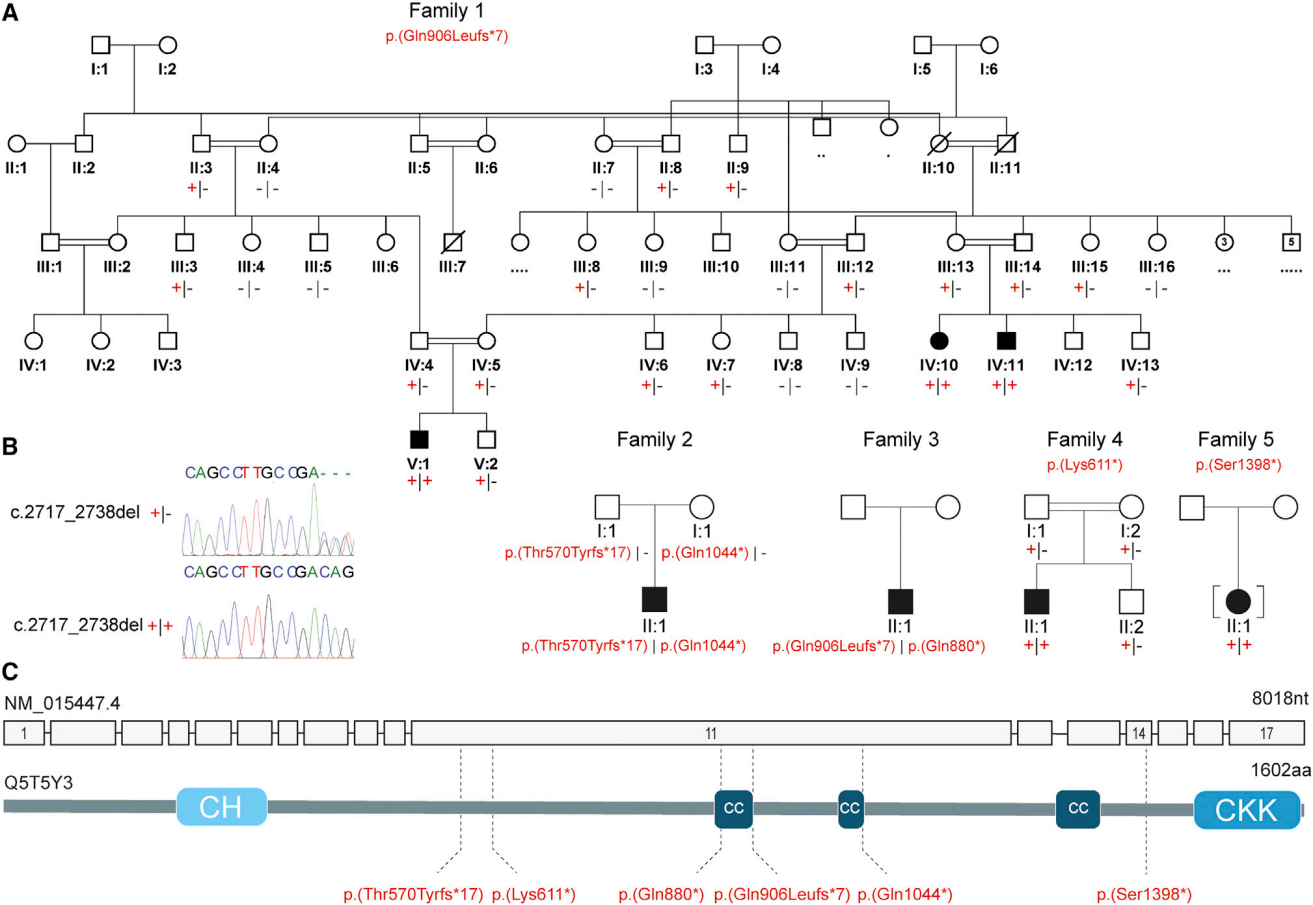
Family pedigrees and bi-allelic *CAMSAP1* variants associated with a syndromic neuronal migration disorder (A) Simplified pedigrees of families in the study showing cosegregation of the variants identified (“–” wild-type allele; “+” familial variant), with reference to transcript GenBank: NM_015447.4. (B) Chromatogram for the c.2717_2738 deletion is shown with heterozygous (top) and homozygous variant (bottom) individuals shown. (C) Intron/exon genomic organization of *CAMSAP1* (top) and protein domain architecture of CAMSAP1 (bottom) illustrating the calponin homology (CH), coiled coil (CC), and calmodulin-regulated spectrin-associated (CKK [CAMSAP1, KIAA1078/CAMSAP2, KIAA1543/CAMSAP3] domain) domains alongside the location of each of the identified pathogenic variants (dotted line).

**Figure 2:**
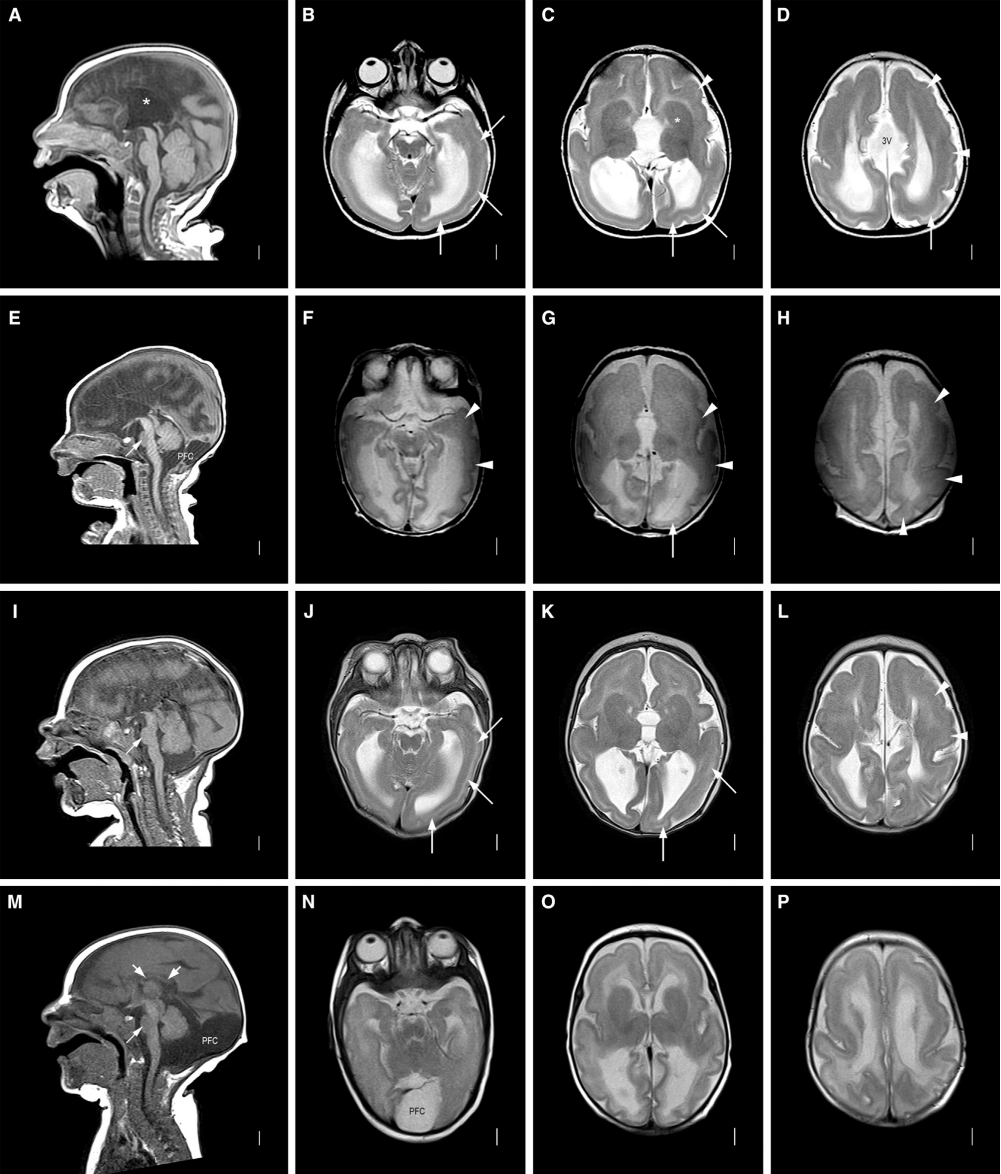
Neuroradiology of affected individuals Neuroimaging in four individuals with the *CAMSAP1*-related neuronal migration disorder (for further imaging see Figure S2): row 1 (A-D) is family 1, V:1 aged 7 months; row 2 (E-H) is family 2, II:1 aged 2 days; row 3 (I-L) is family 3, II:1 aged 3 months; row 4 (M-P) is family 4, II:1 aged 3 months. T1-weighted midline sagittal images show absent (∗ in A, also E and I) or short and thin (short white arrows in M) corpus callosum and small base of the pons (thin white arrow in E, I, and M). Enlarged posterior fossa or “mega cisterna magna” (PFC in E and M) was seen in 2/4 subjects. T2-weighted axial images show posterior-more-severe-than-anterior gradient with areas of agyria or severe pachygyria with prominent cell sparse zones and reduced thickness of the cerebral mantle/wall in posterior regions (white arrows in B–D, G, J, and K) and areas of less severe pachygyria with thicker cerebral mantle/wall in anterior regions (white arrowheads in C, D, F–H, and L). The gradient in family 4, II:1 (N–P) was less clear with lower resolution images. The boundaries of the basal ganglia and thalami were difficult to see, and the internal capsules are not seen (^∗^ in C, also in G, K, and O). The third ventricle was enlarged in all and dramatically enlarged into a midline interhemispheric cyst in family 1, V:1 (3V in D).

We initially identified an extended Palestinian kinship comprising of two interlinking nuclear families with three children aged between 4 months and 3 years 9 months (family 1, V:1, 1V:10, and IV:11, pedigree is shown in Figure 1), affected by a syndromic neuronal migration disorder (recruited with informed consent and Palestinian Health Research Council PHRC/51819 ethical approval). The three children presented with severe primary microcephaly (−4.8 to −6.4 SDS [standard deviation score]), profound global developmental impairment, and craniofacial dysmorphism including large ears, high palate, metopic ridging, and a flat wide nasal bridge (Figure S1). Neurological examination findings included central hypotonia and peripheral hypertonia with brisk reflexes and positive Babinski sign bilaterally. All three affected individuals developed seizures at an early age, which have been refractory to treatment, and the two older children (V:1 and IV:10) have a diagnosis of cortical visual impairment. MRI neuroimaging findings in all three children were consistent and include agyria/severe pachygyria with a posterior greater than anterior gradient, dysmorphic basal ganglia, and absent corpus callosum (Figures 2A–2D for family 1, V:1, Figures S2A–S2D for family 1, IV:11).

To define the genetic cause of disease, exome sequencing was undertaken with DNA from individuals IV:10 and V:1 (Illumina HiSeq and Twist Human Core Exome Kit), assuming homozygosity for a founder variant, although also considering other inheritance mechanisms. SNVs and indels were detected with GATK HaplotypeCaller and annotated with Alamut batch (v.1.8). Copy-number variants were detected with both SavvyCNV^20^ and ExomeDepth (https://github.com/vplagnol/ExomeDepth). Variants failing quality filters or present at a frequency of >0.1% or with >1 homozygous individual in gnomAD (v.2.1.1 or v.3.1.1) or in our in-house database were excluded. Homozygous and compound heterozygous variants common to both affected individuals and present in exons or within ±6 nucleotides in the intron were then evaluated (a full description of the exome bioinformatic pipeline is included in the supplemental methods). A single standout candidate cause of the phenotype was identified, a homozygous variant common to both individuals, Chr9(GRCh38): g.135821923_135821944del (GenBank: NM_015447.4) (*CAMSAP1*) (c.2717_2738del; p.Gln906Leufs^∗^7), located within a ∼8Mb region of homozygosity shared between IV:10 and V:1 [Chr9(GRCh38): g.130299324 to the 9q terminus] (Figure S3). The variant was confirmed by dideoxy sequencing (Figure S4) and found to cosegregate as expected for an autosomal recessive disease in the third affected child (IV:11), parents, and three unaffected siblings. The variant, located in exon 11/17, is predicted to cause a frameshift and premature termination, most likely resulting in nonsense-mediated decay and bi-allelic loss of function.

Through international collaboration (including GeneMatcher),^21^ we identified four additional affected children from four unrelated families, in whom exome sequencing undertaken on Illumina platforms identified bi-allelic predicted loss-of-function *CAMSAP1* variants. These individuals (aged 1–6 years) presented with clinical and neuroradiological features overlapping those of the Palestinian children. Clinical data were obtained with informed consent by local clinicians using a standardized proforma. Clinical findings on the seven affected individuals are summarized in Table 1. The pedigrees and clinical photos depicting morphological features are shown in Figures 1 and S1. Detailed clinical descriptions of all individuals are provided in the supplemental note (case reports). Retrospective analysis of neuroimaging findings (5/5 where complete data were available) was undertaken by author W.B.D. and is shown in Figures 2 and S2. The *CAMSAP1* variants and exome variants identified in each family in this study are listed in Tables S1 and S2, respectively.

**Table 1:** Summary of clinical and neurological features of individuals with *CAMSAP1*-related neuronal migration disorder

**Individual**	**Family 1, V:1**	**Family 1, IV:10**	**Family 1, IV:11**	**Family 2, II:1**	**Family 3, II:1**	**Family 4, II:1**	**Family 5, II:1**
*CAMSAP1* variants (GenBank: NM_015447.4)	homozygous c.2717_2738del (p.Gln906Leufs^∗^7)	homozygous c.2717_2738del (p.Gln906Leufs^∗^7)	homozygous c.2717_2738del (p.Gln906Leufs^∗^7)	c.1707dupT (p.Thr570Tyrfs^∗^17); c.3130C>T (p.Gln1044^∗^)	c.2717_2738del (p.Gln906Leufs^∗^7); c.2638C>T (p.Gln880^∗^)	homozygous c.1831A>T (p.Lys611^∗^)	homozygous c.4193C>G (p.Ser1398^∗^)
Region	Palestine	Palestine	Palestine	North America	North America	Turkey	North America
Sex	M	F	M	M	M	M	F

**Growth**							

Measurement age (y)	3 y	3.8 y	0.3 y	3.6 y	1.1 y	6.4 y	4.8 y
Birth OFC cm (SDS)	microcephaly	microcephaly (1 m)	microcephaly	microcephaly	microcephaly (4 m)	32 (−2.5)	31.8 (−2.2)
Height cm (SDS)	NK	NK	NK	99.1 cm (−0.2)	66 cm (−4.2)	125 cm (+1.3)	106.5 cm (−0.1)
Weight kg (SDS)	NK	NK	NK	13.5 kg (−1.4)	7.7 kg (−14.5)	25 kg (+1.0)	17.3 kg (−0.2)
OFC cm (SDS)	42.7 cm (−4.8)	40.7 cm (−6.4)	microcephaly	45 cm (−4.8)	42 cm (−5.0)	53 cm (−0.2)	42.5 cm (−7.1) OFC at 3.8 y

**Neurology**							

Age at assessment (y)	3 y	3.8 y	0.3 y	5 y	1.7 y	5.5 y	4.8 y
Global dev. delay	profound	profound	profound	severe	severe	severe	severe
Central tone	↓	↓	↓	↓	↓	↓ (severe)	↓
Peripheral tone	↑	↑	NK	↑	↓	↑	↑
Deep tendon reflexes	+++	+++	NK	++; brisk	++	+++	+++
Plantar reflexes	↑	↑	NK	NK	absent	NK	NK
Seizures/age of onset	from 1 m	–	from 2 m	from 4 to 5 m	from 5 m	from 5 m	from 2 m
EEG findings	NK	NK	NK	modified hypsarrhythmia	hypsarrhythmia	burst suppression	multifocal epileptiform discharges
Cortical visual impairment	+	+	NK	+	+	+	+
Feeding difficulties	+	+	+	+	gastrostomy	+	gastrostomy

**Facial features**							

Prominent metopic suture	+	+	+	+	+	–	–
Wide nasal bridge	+	+	+	+	+	+	+
Pronounced cupids bow	+	+	+	+	+	–	+
Large prominent ears	+	+	+	+	+	+	–
High arched palate	+	+	NK	–	NK	+	+

**Neuroimaging**							

Lissencephaly/pachygyria	+	+	+	+	+	+	+
ACC/severe HCC	+	+	+	+	+	+	+
Dysplastic basal ganglia	+	+	+	+	+	+	+
Enlarged posterior fossa	–	–	–	+	–	+	–
Cerebellar hypoplasia	mild	mild	+	+	+	+	mild
Other clinical features	hyperopia with astigmatism, clinodactyly	clinodactyly	none noted	cryptorchidism, scoliosis	none noted	cryptorchidism, femoral hernia	deceased age 5.5 y

Abbreviations: +, feature is present; –, feature is absent; ↑, increased; ↓, decreased; +++, hyperactive; aCC, agenesis of the corpus callosum; cm, centimeters; dev., developmental; DWS, Dandy-Walker syndrome; F, female; hCC, hypogenesis of the corpus callosum; m, months; M, male; NK, not known; OFC, occipitofrontal circumference; SDS, standard deviation score; y, years.

Family 2, II:1 is the eldest child of unaffected, unrelated North American parents of North European ancestry. Antenatal brain imaging showed lissencephaly, agenesis of the corpus callosum (aCC), and a small cerebellum. He was hypotonic at birth and exhibited early feeding difficulties. From 4 months, he was affected by infantile spasms and tonic seizures and his EEG showed a modified hypsarrhythmia pattern. At age 5 years, he has severe global developmental delay (GDD), is unable to sit unsupported, and is non-verbal but uses a gaze tracking device to indicate his needs. Neurological findings include axial hypotonia, peripheral hypertonia, and cortical visual impairment. He has a similar facial gestalt (prominent metopic suture, wide nasal bridge, and prominent cupids bow) to the affected Palestinian children (Figures S1E and S1F). MRI revealed diffuse severe pachygyria with a “posterior more severe than anterior” (P > A) gradient, dysmorphic basal ganglia, absent corpus callosum, and enlarged posterior fossa or "mega cisterna magna" (Figures 2E–2H). He underwent diagnostic trio-exome sequencing through GeneDx (USA), which identified *in trans* compound heterozygous predicted loss-of-function *CAMSAP1* variants [Chr9(GRCh38): g.135822954dupA (GenBank: NM_015447.4) (c.1707dupT; p.Thr570Tyrfs^∗^17) and Chr9(GRCh38): g.135821531G>A (GenBank: NM_015447.4) (c.3130C>T; p.Gln1044^∗^)]. To exclude other potential causes of disease, the exome data were then reanalyzed with the same bioinformatic pipeline and filtering strategy applied to the exome data from family 1 (variant list – Table S2).

Family 3, II:1, a 20-month-old male child born to North American parents of Northern European ancestry, was noted to be microcephalic and display abnormal movements in early infancy. His EEG showed hypsarrhythmia and he was diagnosed with infantile spasms at 5 months of age. His seizures have progressed, requiring polytherapy for effective control. He has severe GDD (crawling, non-verbal, reaching for objects), cortical visual impairment, and generalized hypotonia. He has a history of feeding difficulties requiring nasogastric feeding and parenteral gastrostomy (PEG) placement for an unsafe swallow. His craniofacial features are similar to those of the other affected children. Brain MRI (Figures 2I–2L) revealed pachygyria with thicker cerebral mantle anteriorly, enlarged 3^rd^ ventricle, and dysmorphic basal ganglia and thalami with internal capsule not seen. Singleton diagnostic exome sequencing undertaken through GeneDx (USA) identified compound heterozygous *in trans* predicted loss-of-function variants in *CAMSAP1*, including the same frameshift variant identified in family 1 [Chr9(GRCh38): g.135821923_135821944del (GenBank: NM_015447.4) (c.2717_2738del; p.Gln906Leufs^∗^7)] and a nonsense variant, Chr9(GRCh38): g.135822023G>A (GenBank: NM_015447.4) (c.2638C>T; p.Gln880^∗^).

Family 4, II:1 is a six-year-old child of related Turkish parents, investigated as part of a large cohort study to define candidate genetic causes of neurodevelopmental disorder.^22^ He was found to be microcephalic at birth (−2.5 SDS) and was diagnosed with infantile spasms aged 5 months, before going on to develop multiple other seizure types. His EEG showed a burst-suppression pattern. He has mild craniofacial dysmorphism, profound GDD, central hypotonia, limb spasticity, epilepsy, and cortical visual impairment. Limb movements are described as dyskinetic with varying spasticity of his limbs and intermittent guarded rigidity. MRI revealed diffuse lissencephaly, dysmorphic basal ganglia, a thin corpus callosum and enlarged posterior fossa or “mega cisterna magna” (Figures 2M–2P). Trio-exome sequencing performed at Baylor College of Medicine (USA), as previously described,^22^ identified a candidate homozygous nonsense variant, Chr9(GRCh38): g.135822830T>A (GenBank: NM_015447.4) (c.1831A>T; p.Lys611^∗^), located within a 4.2 Mb region of homozygosity.

Family 5, II:1 is a 4-year-old adopted child, with profound GDD, generalized seizures and severe microcephaly (−7.1 SDS). Neurological examination revealed central hypotonia with bilateral lower limb spasticity and dystonic movements, with craniofacial features including a wide nasal bridge and pronounced cupids bow (Figure S1H). EEG findings of multifocal epileptiform discharges were consistent with electroclinical seizures that appeared to lateralize to either hemisphere. MRI findings include holohemispheric bilateral lissencephaly and grey matter band heterotopia with notable white matter volume loss, a prominent cisterna magna, and diffusely small brainstem with decreased volume of the dorsal pons (Figures S2E and S2F). Proband-only exome sequencing performed at Cincinnati Children’s Hospital Medical Center (CCHMC) with previously described methodology^23^ identified a homozygous *CAMSAP1* variant [Chr9: g.135818055G>C (GenBank: NM _015447.4) (c.4193C>G; p.Ser1398^∗^)].

All the *CAMSAP1* variants identified as part of this study were predicted to result in nonsense mediated mRNA decay and loss of function. The variants were predominantly (5/6) located in the largest exon of the *CAMSAP1* gene (exon 11/17, Figure 1B) and are absent from the genome aggregation database (gnomAD v.2.1.1 and v.3.1.1), with the exception of p.Gln906Leufs^∗^7, present in two unrelated families in this study and one additional heterozygous Finnish individual in gnomAD (Table S1). Further inspection of the genomic architecture surrounding this variant suggests its recurrent nature may result from a homologous recombination event (Figure S5). Furthermore, there are no homozygous loss-of-function *CAMSAP1* variants listed in publicly accessible genomic databases. The exome filtering steps followed in families 3–5 were similar to those described for families 1 and 2 and are detailed in the supplemental methods. In all families, the *CAMSAP1* variants cosegregated as expected for an autosomal recessive trait (Figure 1). In family 3, the closely collocated compound heterozygous variants in *CAMSAP1* (p.Gln906Leufs^∗^7 and p.Gln880^∗^) could be determined to be *in trans* by phasing the short-read exome sequencing data.

**Figure 3:**
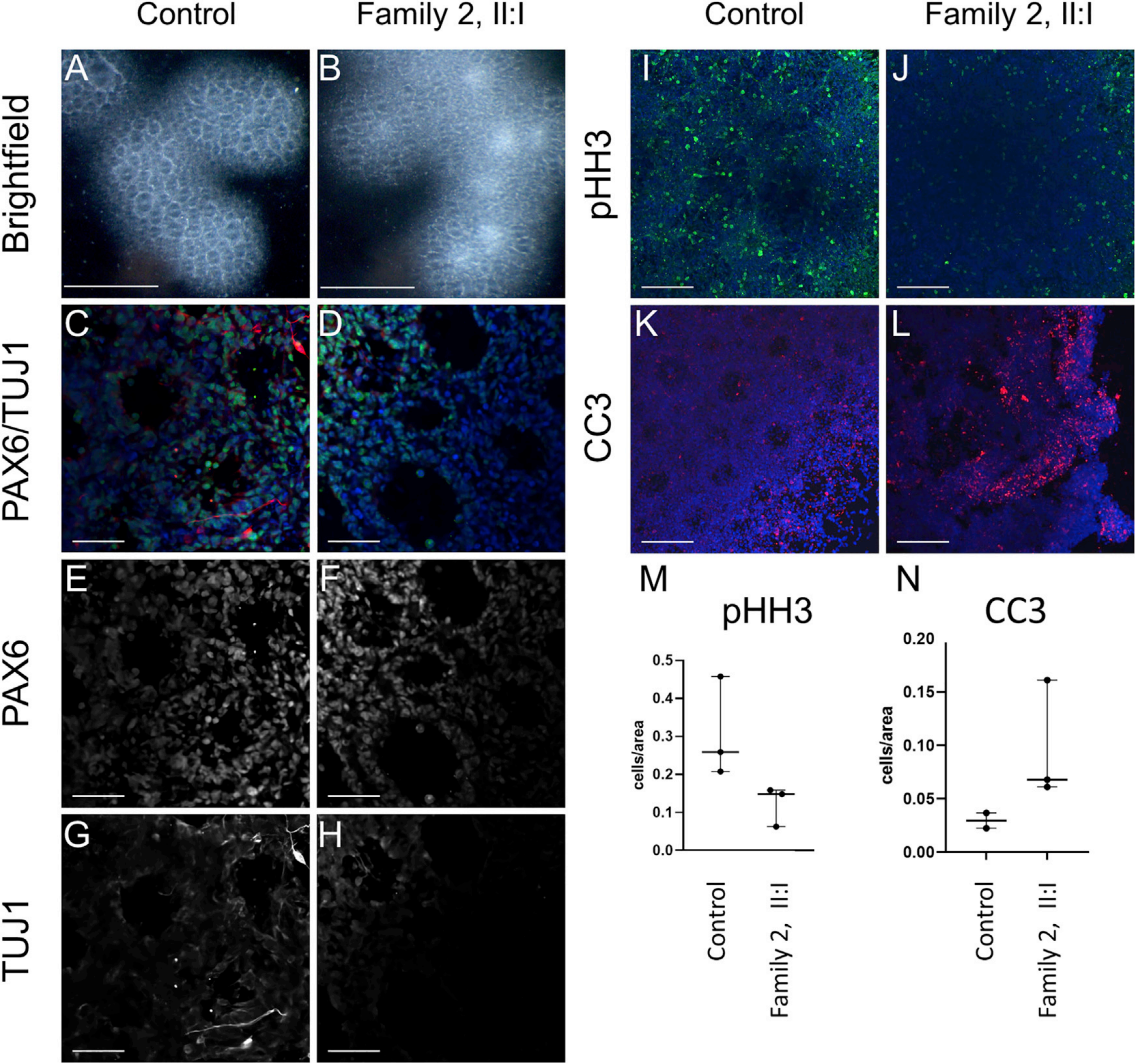
iPSCs from affected individuals display decreased proliferation and differentiation and increased apoptosis of neural progenitor cells (A and B) Brightfield images showing abnormal clustering of cells in rosettes from an affected individual at 11 days *in vitro*. Control cell rosettes have a clearly visible interior region of reduced cell density as compared to more dense cells from the affected individual. Scale bar, 500 μm. (C–L) Immunohistochemistry (IHC) analysis highlights molecular features of affected-individual-derived rosettes. Scale bars, 50 μm. (C–H) IHC for PAX6 (green, a marker of neuronal progenitors), and TUJ1/TUBB3 (red, marks differentiated neurons) demonstrated significantly fewer differentiated neurons at 11 days in rosettes derived from affected individual iPSCs compared to control iPSCs. (E–H) PAX6 (E and F) and TUJ1 (G and H) shown independently. (I and J) IHC for phosphohistone H3 (pHH3) shows reduced staining suggesting a proliferation defect in rosettes derived from iPSCs obtained from an affected individual. (K and L) IHC for cleaved caspase-3 (CC3) demonstrating increased apoptosis in rosettes derived from iPSCs obtained from an affected individual. (M and N) Quantification of counts for PHH3+ cells (n = 3 images × 3 replicates) and CC3+ cells (n = 3 images × 3 replicates). iPSCs from the affected individual were derived from family 2, II:1, one clone of which was received from the Genome Engineering and Stem Cell Center, Department of Genetics, School of Medicine, Washington University in Saint Louis. Control cells are from iPSC line iPSC72.3.

We next investigated the functional consequences of the *CAMSAP1* variants in induced pluripotent stem cells (iPSCs). Peripheral blood mononuclear cells (PBMCs) were isolated from whole blood from an affected individual (family 2, II:1), cultured to enrich erythroid progenitor cells, which were transduced with a Sendai viral cocktail that expresses the Yamanaka factors, Klf4, cMyc, Oct4, and Sox2, to obtain iPSCs. The *CAMSAP1* genotype of the iPSC line was confirmed by next-generation sequencing. These iPSCs were used alongside an iPSC wild-type control cell line (iPSC72.3, CCHMC PSCF) to generate neural rosettes: radially organized two-dimensional structures of neural progenitor cells (NPCs) and differentiating neurons. We analyzed the neural rosettes at both 8 days *in vitro*, when they are entirely composed of progenitor cells, and 11 days *in vitro*, when the progenitors begin to produce differentiating neurons along the outer edge of each rosette. The morphology of rosettes derived from the affected child (family 2, II:2) were abnormal, showing large clusters of cells improperly collecting in the center of each rosette (Figures 3A and 3B). We stained rosettes for PAX6, a marker of neuronal progenitors, and TUJ1 which marks differentiated neurons. These data demonstrated significantly fewer differentiated neurons present at 11 days *in vitro* in rosettes from the affected individual (Figures 3C–3H). Additionally, a proliferation defect was evident by the reduced number of pHH3+ cells present at both 8 and 11 days (Figures 3I–3J). Finally, cleaved caspase-3 (CC3) is upregulated in disease-associated rosettes, indicating an increased level of cell death, which may explain the presence of the dense cell clusters previously noted (Figures 3K–3N). Overall, iPSCs and neural rosettes derived from the affected child with the *CAMSAP1*-related neuronal migration disorder show increased apoptosis, and decreased proliferation and differentiation of neuronal progenitor cells, consistent with the neuronal migration defects observed in affected individuals.

**Figure 4:**
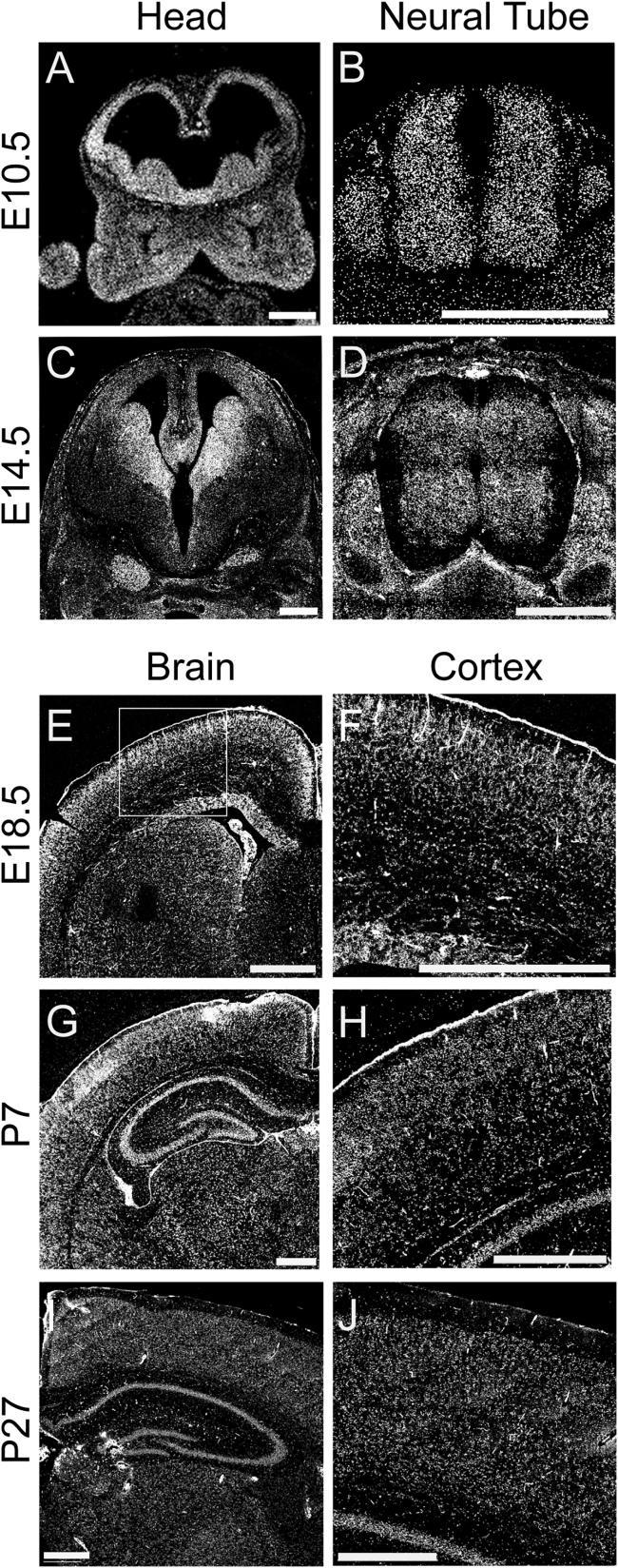
Expression of *Camsap1* in the CNS and developing facial primordia RNAScope probe for *Camsap1* demonstrate robust mRNA expression at E10.5 (A and B) and E14.5 (C and D) in the developing head (A and C), and neural tube (B and D) with a clear enrichment in the neural tissues (A–J) in addition to the developing pharyngeal arches (A). Expression at E18.5 (E and F), P7 (G and H), and P27 (I and J) remains high in the brain with slightly enriched expression in upper layers (F, H, and J are higher magnification views of E, G, and I, respectively). All scale bars, 500 μm.

Despite the availability of RNA sequencing (RNA-seq) datasets describing general expression of the *CAMSAP* genes in mouse and human tissues including during brain development, no detailed temporospatial expression studies of *CAMSAP1* in the developing embryo have been performed. This is relevant given the craniofacial and specific central nervous system (CNS) abnormalities associated with this condition. We thus performed RNAScope *in situ* RNA hybridization in the developing mouse at several stages from embryonic day 10.5 (E10.5) to adulthood (postnatal day 27). At E10.5, *Camsap1* is highly and ubiquitously expressed in the head, particularly in the brain and throughout the first pharyngeal arch, and neural tube (Figures 4A and 4B). At E14.5 or mid-neurogenesis, expression in the brain becomes slightly more localized to the ganglionic eminences and cortex (Figure 4C) and present (albeit somewhat reduced) in the caudal neural tube (Figure 4D). At late stages of neurogenesis (E18.5), *Camsap1* in the brain is expressed most highly in the cortex, particularly in upper layers of differentiated neurons and the ventricular zone (Figures 4E and 4F). Postnatally, *Camsap1* is particularly evident in upper cortical layers and in the hippocampus (Figures 4G–4J). High embryonic expression in the developing face tissues and cortex from E10.5–18.5 would be consistent with craniofacial abnormalities and CNS malformations such as microcephaly and lissencephaly, which we observe in individuals with *CAMSAP1*-related neuronal migration disorder. Postnatal expression in upper cortical layers correlates with previous mouse and rat expression data indicating that *Camsap1* is expressed in specific populations of neurons and astrocytes.^24^

Although previous studies of *Camsap1* knockout mice were robust,^19^ they focused on the laminar organization of the cortex and neuronal polarity and not craniofacial phenotypes. We thus obtained a null allele of *Camsap1* (*Camsap1*^*em1(IMPC)J*^; hereafter referred to as the “null” allele) in order to investigate the wider effects of CAMSAP1 loss in the mouse. Homozygous null mice were examined at birth (P0) and during weaning (P21) (see Figure S6A for conclusive genotyping), revealing that while we observe normal Mendelian survival of *Camsap1*^*null/null*^ animals at embryonic stages E14.5–E18.5 (Figure S6B), *Camsap1*^*null/null*^ animals do not survive in Mendelian ratios postnatally, with only approximately one-third of expected homozygous null animals present at P1 (Figure S6C, n = 3 of an expected 9) and zero of an expected ten at P21 (Figure S6D). We examined surviving *Camsap1*^*null/null*^ animals, finding no overt morphological or skeletal abnormalities (Figures S7A–S7K).

Together our clinical, genetic, murine, and molecular findings define bi-allelic likely loss-of-function variants in *CAMSAP1* as a cause of a recognizable syndromic microcephalic neuronal migration disorder. The cardinal clinical features of the *CAMSAP1*-related neuronal migration disorder in humans include craniofacial dysmorphism comprising of large ears, a prominent metopic suture, wide nasal bridge and pronounced cupids bow (7/7 individuals, Figure S1), primary microcephaly (7/7 individuals, defined as >2 standard deviations below the mean for age and sex; i.e., *Z* score = −2),^25^ severe to profound global developmental delay (7/7 individuals), feeding difficulties (7/7), sometimes requiring gastrostomy (2/7), cortical visual impairment (6/6 for which data was available), and seizures (6/7), typically infantile spasms with onset before one year of age, progressing to other generalized seizures refractory to antiepileptic treatment. Neurological findings although variable, include central hypotonia, with peripheral hypertonia, brisk reflexes, and positive Babinski sign.

The neuroradiological abnormalities (Figure 2) are strikingly consistent across all of the affected individuals in this study. Affected individuals display a classic (thick) lissencephaly with P > A gradient. In posterior regions, areas of agyria or severe pachygyria with prominent cell-sparse zones and reduced thickness of the cerebral mantle/wall are seen, with areas of less severe pachygyria and thicker cerebral mantle/wall in anterior regions. Extra-cortical features include dysplasia of the hippocampus (short, globular, and under-rotated), basal ganglia, and thalami, alongside absence of internal capsule, an absent or extremely short and thin corpus callosum, mild-moderate brainstem hypoplasia, small base of the pons, severe underdeveloped frontal horn (likely due to the basal ganglia abnormalities), enlarged third ventricle, mild borderline enlarged tectum, and cerebellar hypoplasia, although the cerebellar folia pattern remains normal (within the limits of resolution). An enlarged posterior fossa or “mega cisterna magna” was also observed in 2/5 affected individuals (Table 1).

These observed abnormalities combine cortical findings characteristic of *PAFAH1B1*(*LIS1*)-related classic lissencephaly with wider brain findings more in keeping with the tubulinopathies. The classical thick lissencephaly appearance with P > A gradient and prominent cell-sparse zone seen in the *CAMSAP1*-related neuronal migration disorder is analogous to the findings seen in *PAFAH1B1*(*LIS1*)-related lissencephaly^3^ and thus predictive of a four-layer cortical histopathology. However, the extra-cortical malformations are more in keeping with a severe tubulinopathy disorder, most similar to classical *TUBA1A*-related disease.^3^^,^^10^^,^^26^^,^^27^ Notable neuroradiological differences between the *CAMSAP1*-related neuronal migration disorder and the tubulinopathies involve the cerebellar and corpus callosum phenotypes. At the milder end of the tubulinopathies spectrum, cerebellar hypoplasia with an abnormal folia pattern may be the only abnormality present, while folia patterning is apparently preserved in the *CAMSAP1*-related neuronal migration disorder. Conversely, hypogenesis of the corpus callosum is typically seen in the tubulinopathies, whereas agenesis is very rarely described but was universally seen in individuals affected by the *CAMSAP1*-related neuronal migration disorder.^27^ Taken together, the *CAMSAP1*-related neuroradiology may best be described as resembling that of an atypical tubulinopathy but with a distinct and unusual pattern of abnormalities (classic lissencephaly with a P > A gradient and complete agenesis of the corpus callosum), which appear pathognomonic of the disorder.

The close alignment of neuroradiological features between the tubulinopathies, *PAFAH1B1(LIS1)*-related lissencephaly, and the *CAMSAP1*-related neuronal migration disorder is consistent with a shared pathomolecular mechanism underlying these diseases and highlights the role of the minus end of the microtubule in neuronal migration disorders. PAFAH1B1(LIS1) facilitates the function of the minus-end-directed microtubule motor dynein, and mutations in the heavy chain of cytoplasmic dynein (DYNC1H1) are associated with a neuronal migration disorder that may also show P > A gradient of lissencephaly in addition to extra-cortical malformations.^3^^,^^28^ Dynein is a molecular motor that traffics substrates and organelles to this microtubule minus end, whereas CAMSAP proteins play a stabilizing role and tubulins are the core structural component.^11^^,^^16^^,^^17^

There are, however, distinct clinical, neuroradiological, and pathophysiological features only associated with the *CAMSAP1*-related neuronal migration disorder. We thus investigated the temporospatial expression of *Camsap1* to better understand these phenotypes. Previous studies have identified high *Camsap1* expression in the cortex, subventricular zone, and hippocampus of mice and rats^19^^,^^24^ and in neurons, astrocytes, and their precursor neuronal stem cells.^24^^,^^29^ Our murine brain expression studies determined that *Camsap1* is widely expressed early in neurogenesis in the developing brain and neural tube, in keeping with the wide range of brain malformations observed in the disorder. In addition, the fine spatial resolution that our methods enable highlight CAMSAP1 specificity to the ganglionic eminences, ventricular zone, and outer cortical layers throughout embryonic neurogenesis, in keeping with a role in neuronal migration. Supporting this, postnatal *Camsap1* expression remains evident in the outer cortex and hippocampus, the endpoints of these migration routes. The normal development of these structures is critically dependent on precisely controlled molecular mechanisms, which govern the highly intricate neuronal migration events ongoing during these developmental stages.^30^ Our findings thus implicate *Camsap1* in these neuronal migration processes, potentially explaining the clinical findings in affected individuals with the *CAMSAP1*-related neuronal migration disorder.

Previously studied *Camsap1* knockout mice generated by Zhou et al. identified radial migration defects of cortical neurons leading to cortical laminar disorganization and a significant increase of neurons in the intermediate zone after the completion of neuronal migration.^19^ Although mice do not have sufficiently folded brains to adequately model human lissencephaly, the identification of impaired neuronal migration by Zhou et al.^19^ is highly consistent with the lissencephaly phenotype identified here. The mice were born in Mendelian ratios, although they exhibited reduced brain and body size and a high level of early postnatal mortality resulting from seizures in the immediate postnatal period.^19^ This phenotype is analogous to the early and severe forms of epilepsy invariably observed in individuals with the *CAMSAP1*-related neuronal migration disorder (Table 1), with the lack of perinatal mortality observed in humans potentially explained by the efficacy of pharmacological anti-epileptic therapies. Our studies of *Camsap1*^null/null^ mice indicate a significantly increased mortality occurring in the third trimester or at birth for null animals, with survival into the second trimester (E14.5–E18.5) unaffected. This may reflect a variation in phenotype severity, leading to prenatal seizures or CNS abnormalities affecting fundamental physiological processes and related to differences in the genomic knockout strategies and/or potentially different genetic strains used in generating each murine model. Alternatively, stochastic variation of expression of a dosage-sensitive gene may play a role. While we did not observe gross anatomical or histological changes in the second trimester (E14.5–E18.5), postnatal histological findings in the mice generated by Zhou et al. suggest that the absence of CAMSAP1 leads to disorganization of cortical layering.^19^

Histological studies in mammalian neurons have established that CAMSAP1 inhibits neurite extension^12^ and is critical for the differentiation of neurites into mature axons and dendrites.^19^ A proposed mechanism for ensuring the development of (typically) a single neuronal axon involves the accumulation of CAMSAP1-associated microtubule clusters in the longest neurite, given the finding that depletion of CAMSAP1 results in an abnormal multi-axon phenotype.^19^ The kinase MARK2, which phosphorylates CAMSAP1, controlling its ability to bind microtubules, appears to regulate this process.^19^ Additionally, a role in neuronal polarization and axonal/dendritic differentiation may not be unique to CAMSAP1: other studies also implicate a similar role for CAMSAP2 and CAMSAP3^31^ in this process.^17^^,^^18^ Other previous *in vitro* and animal studies have suggested roles for CAMSAP1 in mammalian neuronal polarization, axon/dendrite differentiation, and cytotaxis comprising important elements of neuronal migration, findings that are now consolidated by our studies.

The discovery of a non-tubulin cause of a tubulinopathy-like disorder highlights other microtubule-associated proteins, including microtubule minus-end-targeting proteins, as candidate genetic causes of neuronal migration disorders.

## Data Availability

Full exome sequencing data are not available because of reasons of confidentiality; anonymized variant data will be made available on reasonable request. The authors declare that all other data are contained within the manuscript and supplemental materials. The accession numbers for the *CAMSAP1* variants reported in this paper are ClinVar: SCV002574746–SCV002574751.

## Supplemental information

Document S1. Figures S1–S9, Tables S1–S3, supplemental notes, and supplemental methods

Document S2. Article plus supplemental information

## References

[1] Oegema R., Barakat T.S., Wilke M., Stouffs K., Amrom D., Aronica E., Bahi-Buisson N., Conti V., Fry A.E., Geis T. (2020). International consensus recommendations on the diagnostic work-up for malformations of cortical development. Nat. Rev. Neurol..

[2] Severino M., Geraldo A.F., Utz N., Tortora D., Pogledic I., Klonowski W., Triulzi F., Arrigoni F., Mankad K., Leventer R.J. (2020). Definitions and classification of malformations of cortical development: practical guidelines. Brain.

[3] Di Donato N., Chiari S., Mirzaa G.M., Aldinger K., Parrini E., Olds C., Barkovich A.J., Guerrini R., Dobyns W.B. (2017). Lissencephaly: Expanded imaging and clinical classification. Am. J. Med. Genet..

[4] Fry A.E., Cushion T.D., Pilz D.T. (2014). The genetics of lissencephaly. Am. J. Med. Genet. C Semin. Med. Genet..

[5] Smith D.S., Niethammer M., Ayala R., Zhou Y., Gambello M.J., Wynshaw-Boris A., Tsai L.H. (2000). Regulation of cytoplasmic dynein behaviour and microtubule organization by mammalian Lis1. Nat. Cell Biol..

[6] Di Donato N., Timms A.E., Aldinger K.A., Mirzaa G.M., Bennett J.T., Collins S., Olds C., Mei D., Chiari S., Carvill G. (2018). Analysis of 17 genes detects mutations in 81% of 811 patients with lissencephaly. Genet. Med..

[7] Caspi M., Atlas R., Kantor A., Sapir T., Reiner O. (2000). Interaction between LIS1 and doublecortin, two lissencephaly gene products. Hum. Mol. Genet..

[8] Kumar R.A., Pilz D.T., Babatz T.D., Cushion T.D., Harvey K., Topf M., Yates L., Robb S., Uyanik G., Mancini G.M.S. (2010). TUBA1A mutations cause wide spectrum lissencephaly (smooth brain) and suggest that multiple neuronal migration pathways converge on alpha tubulins. Hum. Mol. Genet..

[9] Desikan R.S., Barkovich A.J. (2016). Malformations of cortical development. Ann. Neurol..

[10] Romaniello R., Arrigoni F., Fry A.E., Bassi M.T., Rees M.I., Borgatti R., Pilz D.T., Cushion T.D. (2018). Tubulin genes and malformations of cortical development. Eur. J. Med. Genet..

[11] Hendershott M.C., Vale R.D. (2014). Regulation of microtubule minus-end dynamics by CAMSAPs and Patronin. Proc. Natl. Acad. Sci. USA..

[12] Baines A.J., Bignone P.A., King M.D.A., Maggs A.M., Bennett P.M., Pinder J.C., Phillips G.W. (2009). The CKK domain (DUF1781) binds microtubules and defines the CAMSAP/ssp4 family of animal proteins. Mol. Biol. Evol..

[13] Pavlova G.A., Razuvaeva A.V., Popova J.V., Andreyeva E.N., Yarinich L.A., Lebedev M.O., Pellacani C., Bonaccorsi S., Somma M.P., Gatti M., Pindyurin A.V. (2019). The role of Patronin in Drosophila mitosis. BMC Mol. Cell Biol..

[14] Akhmanova A., Hoogenraad C.C. (2015). Microtubule minus-end-targeting proteins. Curr. Biol..

[15] King M.D.A., Phillips G.W., Bignone P.A., Hayes N.V.L., Pinder J.C., Baines A.J. (2014). A conserved sequence in calmodulin regulated spectrin-associated protein 1 links its interaction with spectrin and calmodulin to neurite outgrowth. J. Neurochem..

[16] Atherton J., Jiang K., Stangier M.M., Luo Y., Hua S., Houben K., van Hooff J.J.E., Joseph A.-P., Scarabelli G., Grant B.J. (2017). A structural model for microtubule minus-end recognition and protection by CAMSAP proteins. Nat. Struct. Mol. Biol..

[17] Jiang K., Hua S., Mohan R., Grigoriev I., Yau K.W., Liu Q., Katrukha E.A., Altelaar A.F.M., Heck A.J.R., Hoogenraad C.C., Akhmanova A. (2014). Microtubule minus-end stabilization by polymerization-driven CAMSAP deposition. Dev. Cell.

[18] Yau K.W., van Beuningen S.F.B., Cunha-Ferreira I., Cloin B.M.C., van Battum E.Y., Will L., Schätzle P., Tas R.P., van Krugten J., Katrukha E.A. (2014). Microtubule minus-end binding protein CAMSAP2 controls axon specification and dendrite development. Neuron.

[19] Zhou Z., Xu H., Li Y., Yang M., Zhang R., Shiraishi A., Kiyonari H., Liang X., Huang X., Wang Y. (2020). CAMSAP1 breaks the homeostatic microtubule network to instruct neuronal polarity. Proc. Natl. Acad. Sci. USA..

[20] Laver T.W., De Franco E., Johnson M.B., Patel K.A., Ellard S., Weedon M.N., Flanagan S.E., Wakeling M.N. (2022). SavvyCNV: Genome-wide CNV calling from off-target reads. PLoS Comput. Biol..

[21] Sobreira N., Schiettecatte F., Valle D., Hamosh A. (2015). GeneMatcher: a matching tool for connecting investigators with an interest in the same gene. Hum. Mutat..

[22] Mitani T., Isikay S., Gezdirici A., Gulec E.Y., Punetha J., Fatih J.M., Herman I., Akay G., Du H., Calame D.G. (2021). High prevalence of multilocus pathogenic variation in neurodevelopmental disorders in the Turkish population. Am. J. Hum. Genet..

[23] Liegel R.P., Finnerty E., Blizzard L., DiStasio A., Hufnagel R.B., Saal H.M., Sund K.L., Prows C.A., Stottmann R.W. (2019). Using human sequencing to guide craniofacial research. Genesis.

[24] Yamamoto M., Yoshimura K., Kitada M., Nakahara J., Seiwa C., Ueki T., Shimoda Y., Ishige A., Watanabe K., Asou H. (2009). A new monoclonal antibody, A3B10, specific for astrocyte-lineage cells recognizes calmodulin-regulated spectrin-associated protein 1 (Camsap1). J. Neurosci. Res..

[25] Centers for Disease Control and Prevention (2020). Facts about Microcephaly. https://www.cdc.gov/ncbddd/birthdefects/microcephaly.html.

[26] Bahi-Buisson N., Poirier K., Fourniol F., Saillour Y., Valence S., Lebrun N., Hully M., Bianco C.F., Boddaert N., Elie C. (2014). The wide spectrum of tubulinopathies: what are the key features for the diagnosis?. Brain.

[27] Hebebrand M., Hüffmeier U., Trollmann R., Hehr U., Uebe S., Ekici A.B., Kraus C., Krumbiegel M., Reis A., Thiel C.T., Popp B. (2019). The mutational and phenotypic spectrum of TUBA1A-associated tubulinopathy. Orphanet J. Rare Dis..

[28] Splinter D., Razafsky D.S., Schlager M.A., Serra-Marques A., Grigoriev I., Demmers J., Keijzer N., Jiang K., Poser I., Hyman A.A. (2012). BICD2, dynactin, and LIS1 cooperate in regulating dynein recruitment to cellular structures. Mol. Biol. Cell.

[29] Yoshioka N., Asou H., Hisanaga S.I., Kawano H. (2012). The astrocytic lineage marker calmodulin-regulated spectrin-associated protein 1 (Camsap1): phenotypic heterogeneity of newly born Camsap1-expressing cells in injured mouse brain. J. Comp. Neurol..

[30] Ayala R., Shu T., Tsai L.-H. (2007). Trekking across the Brain: The Journey of Neuronal Migration. Cell.

[31] Toya M., Kobayashi S., Kawasaki M., Shioi G., Kaneko M., Ishiuchi T., Misaki K., Meng W., Takeichi M. (2016). CAMSAP3 orients the apical-to-basal polarity of microtubule arrays in epithelial cells. Proc. Natl. Acad. Sci. USA..

