# Supplementary material for: Bi-allelic CAMSAP1 variants cause a clinically recognizable neuronal migration disorder
Source: Am J Hum Genet. 2022 Oct 24;109(11):2068–79. doi: 10.1016/j.ajhg.2022.09.012 (PMC9674946; doi:10.1016/j.ajhg.2022.09.012)
Supplement: Document S1. Figures S1–S9, Tables S1–S3, supplemental notes, and supplemental methods [file mmc1.pdf]

The American Journal of Human Genetics, Volume *109*

Supplemental information

Bi-allelic *CAMSAP1* variants cause a clinically recognizable neuronal migration disorder

Reham Khalaf-Nazzal, James Fasham, Katherine A. Inskeep, Lauren E. Blizzard, Joseph S. Leslie, Matthew N. Wakeling, Nishanka Ubeyratna, Tadahiro Mitani, Jennifer L. Griffith, Wisam Baker, Fida' Al-Hijawi, Karen C. Keough, Alper Gezdirici, Loren Pena, Christine G. Spaeth, Peter D. Turnpenny, Joseph R. Walsh, Randall Ray, Amber Neilson, Evguenia Kouranova, Xiaoxia Cui, David T. Curiel, Davut Pehlivan, Zeynep Coban Akdemir, Jennifer E. Posey, James R. Lupski, William B. Dobyns, Rolf W. Stottmann, Andrew H. Crosby, and Emma L. Baple

Supplemental Note

Detailed clinical summaries

Family 1, IV:10 is a three-year-old girl born to first-cousin parents at full term following an uncomplicated pregnancy. At one month of age, she was noted to be microcephalic with absent movement on her right side and increased tone. At three years and nine months of age she had made no developmental progress and made no eye contact. She startles to noise but has no verbal or non-verbal communication. She was severely microcephalic (-6.4 SDS) with large ears, gingival hyperplasia, high palate, metopic ridging, a flat wide nasal bridge (**Fig. S1A-B**) and bilateral fifth finger clinodactyly. Neurological examination revealed generally increased peripheral tone with upgoing planter reflexes; her right arm adopts a rigid extensor posture.

Her younger male sibling (**IV:11**) was found to have microcephaly with agenesis of the corpus callosum (aCC) on an antenatal ultrasound scan at 22 weeks gestation. He developed intractable epilepsy at nine weeks and was noted to have similar craniofacial dysmorphism to his sister.

A male second cousin (**V:1**) now aged three years, was born at 36 weeks gestation weighing 2kg (-1.8 SDS), microcephaly and shortened long bones were identified on antenatal scans. He had his first generalized seizure at six weeks of age, at three years these continue to be refractory to anti-epileptic treatment. Like his cousins, he has profound global developmental delay (GDD). He displayed extreme irritability and has suffered repeated, complicated respiratory tract infections. He is severely microcephalic (-4.8 SDS) with a prominent synophrys, metopic ridging, a flat wide nasal bridge, large ears, gingival hyperplasia, high palate (**Fig. S1C-D**) and fifth finger clinodactyly bilaterally. Neurological findings include generalized hypertonia, hyperreflexia and positive Babinski sign bilaterally.

MRI neuroimaging findings in all three children (**IV:10, IV:11** and **V:1**) are consistent and include agyria/severe pachygyria with a posterior-greater-than-anterior gradient, dysmorphic basal ganglia and absent corpus callosum (V-1: **Fig. 2A-D,** IV-11: **S2A-D**).

Family 2, II:1 is a 5-year-old male, the eldest of two siblings born to unaffected, unrelated North American parents of North European ancestry (**Fig. 1**). Antenatal brain imaging showed lissencephaly, absent corpus callosum and a small cerebellum. His birth at 38+2 weeks was uncomplicated, although he was subsequently noted to be hypotonic and exhibited early feeding difficulties.

Probable tonic seizure activity began at four-to-five months old as ocular roving/deviation, arm extension, and truncal extension in addition to infantile spasms. His electroencephalogram (EEG) showed diffuse beta frequencies, poor organization and right parietal interictal discharges. Seizure activity was refractory to initial levetiracetam monotherapy which was discontinued. Repeat EEG was performed that demonstrated a “modified hypsarrhythmia pattern” classically associated with West Syndrome. A 30-day prednisolone taper stabilized this pattern; seizure activity resolved and was maintained with vigabatrin and clobazam dual-therapy for approximately 18 months. Thereafter there was good control on clobazam monotherapy

(0.5-0.6 mg/kg/day). Recently breakthrough seizure activity has occurred requiring supplementation of the treatment regimen with Topiramate (~2 mg/Kg BD). At 3 years 7 months of age growth parameters were: Height 99.1 cm (-0.2 SDS), 13.5 kg (-1.4 SDS), 45 cm (-4.8 SDS).

Aged 5 years his neurodevelopment is severely, globally, delayed despite extensive physical therapy and without regression. He can roll and support his head and trunk for short periods, although cannot sit unsupported or crawl. He tolerates 30-45 minutes of upright weight-bearing daily using a standing aid and demonstrates slow gait/locomotion while supported in a gait trainer. His axial tone is hypotonic with variable spasticity of his limbs with intermittent guarded rigidity. He has been treated with botulinum toxin IM injections, which caused unacceptable side effects, and is currently trialing Carbidopa/Levodopa (Sinemet). He has a 15° thoracic rotoscoliosis deformity of his spine and severe left hip dysplasia, with corrective surgical intervention is planned for the latter at around five and a half years of age. He can sometimes reach for toys, recognizes a few words (“book”) and uses a gaze tracking device to make choices between two or three options on a screen, but his productive speech is limited to babbles and consonant sounds without purposeful phonation of words.

There have been longstanding feeding difficulties and per oral intake consists of thickened liquids from a “sippy cup” and pureed diet via flat-plastic spoon. Steady state and flash visual evoked potential (SSVEP and FVEP) demonstrate mild cortical vision impairment and ophthalmoscopy demonstrates normal appearance of optic discs bilaterally. He also has hyperopia with astigmatism for which he wears corrective lens. There have been no concerns regarding his hearing. He was born with left unilateral cryptorchidism and has been affected by chronic constipation. Examination findings demonstrated central hypotonia with peripheral spasticity and brisk reflexes without ankle clonus.

MRI (**Fig. 2E-H)** performed on day two revealed diffuse severe pachygyria with a “posterior more severe than anterior” (P>A) gradient, dysmorphic basal ganglia, absent corpus callosum and enlarged posterior fossa or "mega cisterna magna" (**Fig. 2E-H**)

Diagnostic trio exome was performed at GeneDx (U.S.A) using a proprietary targeting system and a custom developed analysis tool (Xome analyzer), with raw data later reanalyzed in Exeter for robustness using the same pipeline as for Family 1. This identified no plausible variants in known disease genes but did reveal novel, *in trans* compound heterozygous *CAMSAP1* variants in exon 11/17: a paternally inherited Chr9(GRCh38):g.135822954dupA NM_015447.4:c.1707dupT; p.(Thr570TyrFs) variant, and a maternal Chr9(GRCh38):g.135821531G>A NM_015447.4:c.3130 C>T; p.(Gln1044*) variant also in exon 11.

Family 3, II:1 is the only child of North American parents of Northern European origin, born at 41 weeks gestation with a normal birthweight (3.27kg). Abnormal movements were noticed in early infancy and diagnosed as infantile spasms at 5 months of age. Spasms continues and additional seizure semiologies were noted over time including focal episodes of arm posturing and head turning with chaotic eye movements, asymmetric tonic seizures, asymmetric epileptic spasms and occasional epileptic status. Seizures eventually responded to a combination of vigabatrin, clobazam, levetiracetam and zonisamide, following unsuccessful trials of steroids and topiramate. Cannabidiol was also trialed with unclear benefit. Ketogenic diet was not

attempted due to chronic intolerance of other formulas. EEG showed hypsarrhythmia. Microcephaly was noted at an early age with severe delay to neurodevelopment subsequently apparent without evidence of neurodevelopmental regression – at age one there was very poor trunk/head control and intermittent extensor arm posturing, but no purposeful movements and no communication. At 1 year 1 month his growth parameters were: Height 66cm (-4.2 SDS), weight 7.7kg (-14.5 SDS), OFC 42cm (-5.0 SDS).

At 1 year 8 months he has started turning his head purposefully, occasionally reaching for and picking up objects with his hands and scooting in crawling position. He has been diagnosed with cortical visual impairment with poor eye tracking and deprivation nystagmus. He was affected by severe feeding difficulties and there were concerns about aspiration risk requiring nasogastric feeding with a parenteral gastrostomy (PEG) placed at 15 months of age. On examination he has decreased central and peripheral tone with symmetrical, normal deep tendon reflexes and absent Babinski sign.

MRI neuroimaging (**Fig. 2I-L**) revealed pachygyria with thicker cerebral mantle anteriorly, enlarged 3rd ventricle and dysmorphic basal ganglia and thalami with internal capsule not seen.

Proband-only exome was performed at GeneDx (U.S.A), identified compound heterozygous predicted loss-of-function variants in *CAMSAP1,* including the same frameshift variant identified in Family 1 [Chr9(GRCh38):g.135821923_135821944del NM_015447.4:c.2717_2738del; p.(Gln906Leufs*7)] and a novel nonsense variant Chr9(GRCh38):g.135822023G>A NM_015447.4:c.2638C>T; p.(Gln880*). These were proven to be *in trans* using the short-read exome sequencing data and parental segregation was not required. A previously reported pathogenic heterozygous variant in *POLG* [NM_002693.2:c.202C>T; p.(Gln68*), inheritance unknown] was also identified. This variant has been published twice previously, in both cases associated with mitochondrial DNA depletion syndrome 4A (MIM: 203700), also known as Alpers–Huttenlocher syndrome, when *in trans* with another pathogenic *POLG* variant and with transmitting parents unaffected.[1; 2] Since no second pathogenic *POLG* variant could be identified in this individual this is unlikely to be the cause of their neurodevelopmental disorder.

Family 4, II:1 is a male child of related Turkish parents. He was born at full term weighing 3.26kg (-0.3 SDS), length 49cm (-0.6 SDS) and with a head circumference of 32 cm (-2.5 SDS). He presented with severe developmental delay, central hypotonia, limb spasticity, epilepsy and relative microcephaly. Seizures were first observed at 5 months of age as infantile spasms then progressing to other semiology, occurring most frequently on waking and in the early morning and requiring Sodium Valproate, Vigabatrin, Clobazam for control. EEG showed a burst suppression pattern.

At 5 years 6 months he has no head control or other gross motor development, no fine motor development, cortical visual impairment and an unsafe swallow to both liquids and solids. He makes sounds, but these are without clear meaning and has no communicative language. Limb movements are dyskinetic with varying spasticity of his limbs and intermittent guarded rigidity. Central tone is severely reduced, with peripheral spasticity and hyperactive deep tendon reflexes. He has a prominent and wide nasal root, relatively large ears, an open mouth with high arched palate and left sided unilateral ptosis.

At 6 years 5 months growth parameters were: Height 125cm (+1.3 SDS), weight 25kg (+1.0 SDS) OFC 53cm (-0.2 SDS).

Brain MRI (**Fig. 2M-P**) revealed diffuse lissencephaly, dysmorphic basal ganglia, a thin corpus callosum and an enlarged posterior fossa or "mega cisterna magna".

Trio exome sequencing at Baylor College of Medicine, using previously described methods),[3] identified a novel homozygous candidate nonsense variant, Chr9(GRCh38):g.135822830T>A NM_015447.4:c.1831A>T; p.(Lys611*), located within a 4.2Mb region of homozygosity.

Family 5, II:1 was the adopted child of North American parents, only limited information was available regarding her biological parents. She was born at 41+1 weeks gestation following a high-risk pregnancy with a birthweight within the normal range, and head circumference of 31.8cm (-2.2 SDS). Neurodevelopment was reported as severely delayed, rolling first at 3y10m, no speech at 4y9m and dependent for all activities of daily living (Gross Motor Function Classification System (GMFCS) level V. Dystonia, presenting as neck extension and back arching, was diagnosed at 11m of age and managed with diazepam, gabapentin, and baclofen. Seizures semiologies include complex partial seizures involving the right size and unresponsive episodes with associated eye rolling, likely generalized seizures. These were associated with EEG findings of multifocal epileptiform discharges suggesting electroclinical seizures that appeared to lateralize to either hemisphere and have been treated with vigabatrin and diazepam for extended seizures. Neurological examination revealed central hypotonia with bilateral lower extremity spasticity, and investigations were consistent with cortical visual impairment. Her hearing was normal when assessed at 11m of age. At 4 years and 9 months she was no longer able to roll her growth parameters were: Height 106.5cm (-0.1 SDS), weight 17.3kg (-0.2 SDS) and OFC 42.5cm (-7.1 SDS). During her 5th year of life she developed episodes of "emesis with dark fluid", the family opted for hospice care after an extensive evaluation for her symptoms and she died at 5.5 years of age.

Brain MRI findings (**Fig. S2E-F**) included holohemispheric bilateral lissencephaly and grey matter band heterotopia with notable white matter volume loss, a prominent cisterna magna and diffusely small brainstem with decreased volume of the dorsal pons.

Proband only exome sequencing, performed at Cincinnati Children's Hospital Medical Center and analyzed using VarSeq 2.2.3 from Golden Helix identified a homozygous *CAMSAP1* variant [Chr9:g.135818055G>C NM _015447.3: c.4193C>G p.(Ser1398*).

Supplemental Figures

Figure S1: Facial features of individuals with *CAMSAP1*-related disorder

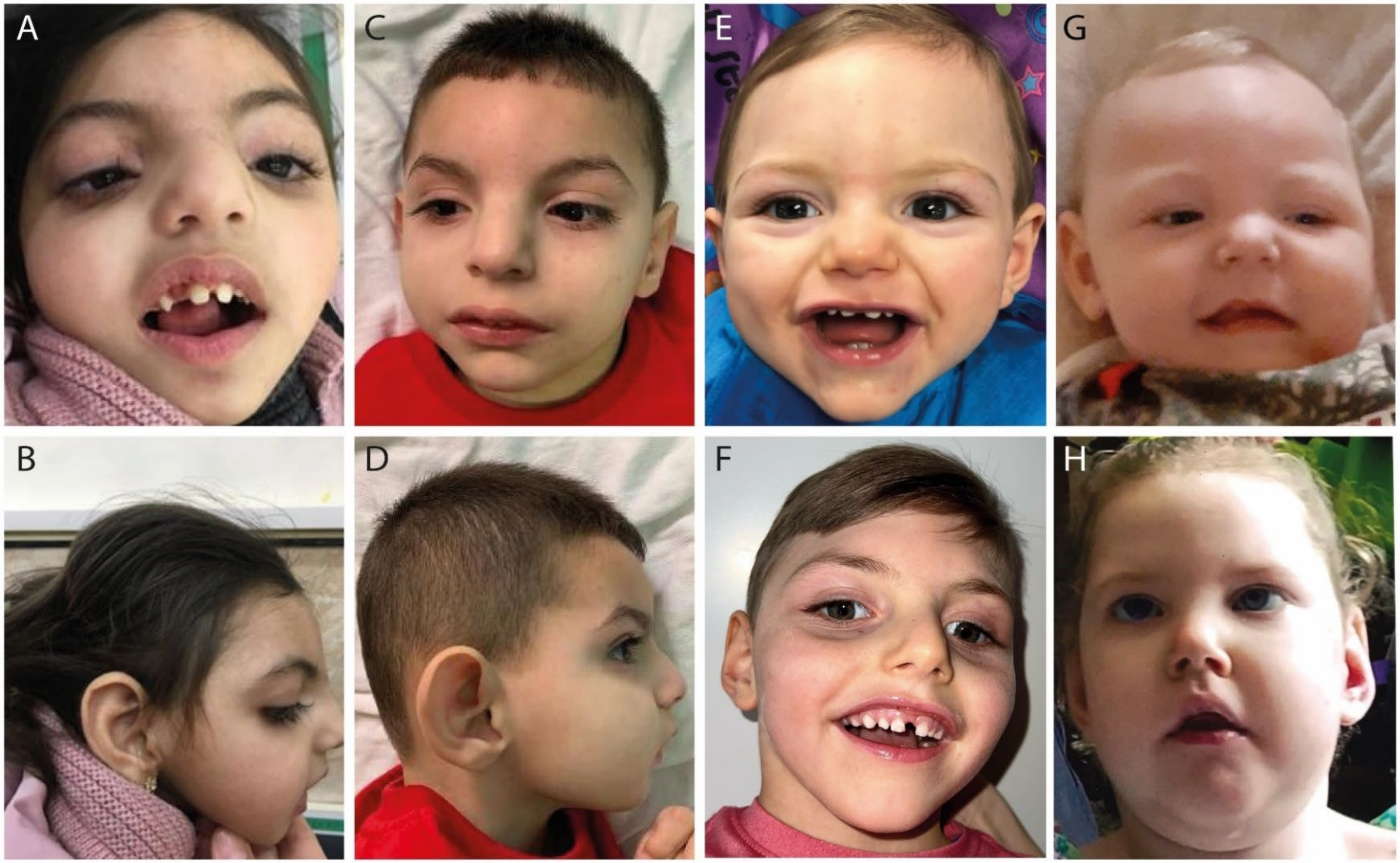

Clinical photographs demonstrating the cardinal features of the *CAMSAP1*-related disorder including microcephaly, large ears, prominent metopic suture, wide nasal bridge and pronounced cupids bow. **(A,B)** Family 1, Individual IV:10; **(C,D)** Family 1, Individual IV:11; **(E,F)** Family 2, Individual II:1; **(G)** Family 3, Individual II:1; **(H)** Family 5, Individual II:1.

Figure S2: Additional MRI brain images from individuals with *CAMSAP1*-related disorder

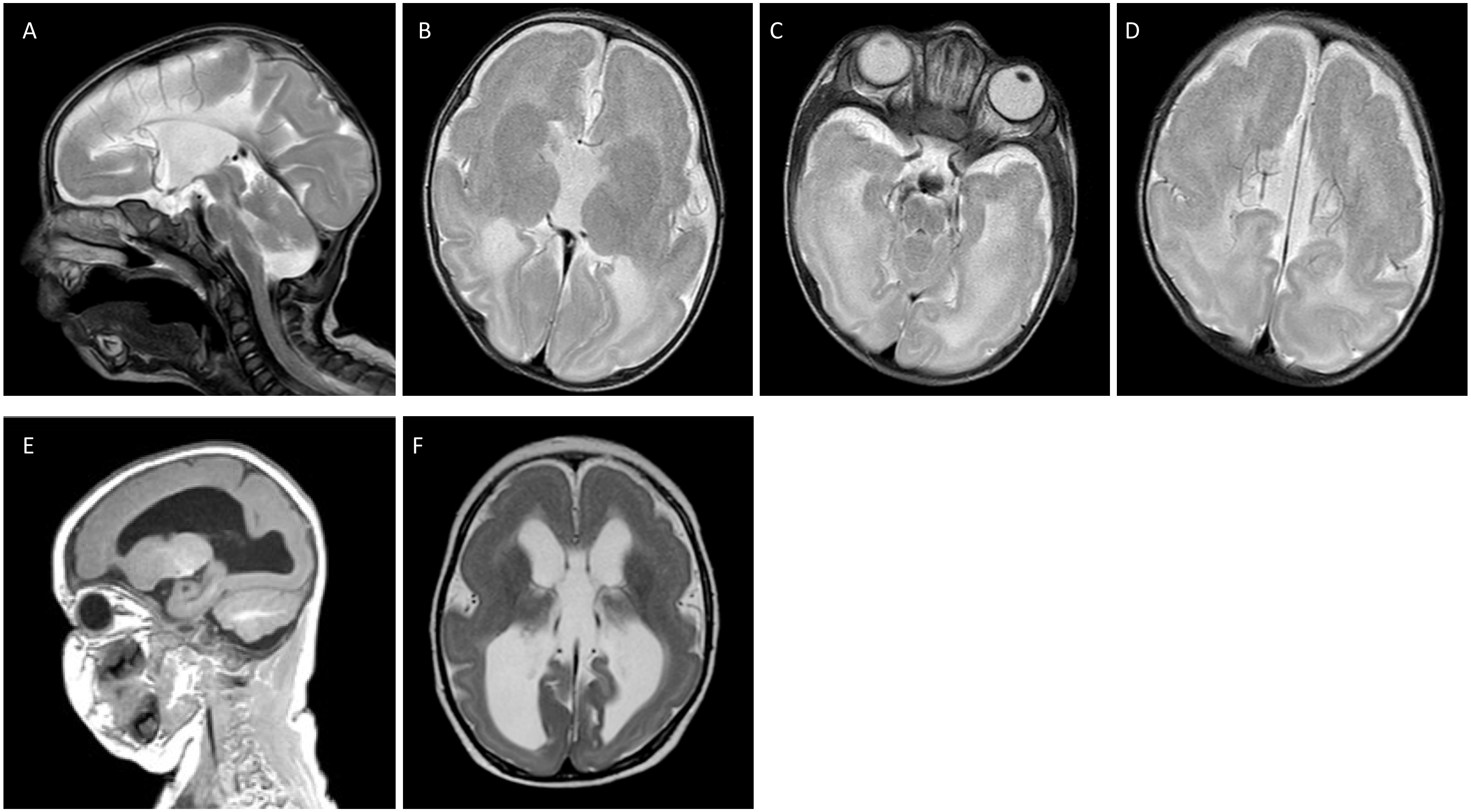

Row 1 (**A-D**) **Family 1, IV-11** aged 4 months, showing agenesis of the corpus callosum, dysmorphic basal ganglia, posterior-more-severe-than-anterior gradient pachygyria and cerebellar hypoplasia.

Row 2 (**E-F**) **Family 5, II-1.** In this case full imaging was not available for re-review, but report and static images were reviewed. There is holohemispheric bilateral lissencephaly with notable white matter volume loss and a prominent cisterna magna.

Figure S3: Homozygous regions greater than 1Mb shared between individuals IV:10 and V:1, generated using AutoMap[4]

Chr	Start	End	Mb
1	57,383,315	59,042,181	1.7
2	52,920,931	68,274,510	15.4
4	142,651,159	145,573,954	2.9
6	163,545,674	170,892,919	7.3
7	97,488,146	99,023,246	1.5
9	133,061,603	141,071,475	8.0

Chr	Start	End	Mb
12	51,585,601	52,680,008	1.1
16	58,537,897	62,851,413	4.3
20	853,502	1,961,134	1.1
21	39,764,437	41,029,831	1.3
22	27,146,768	28,293,980	1.1

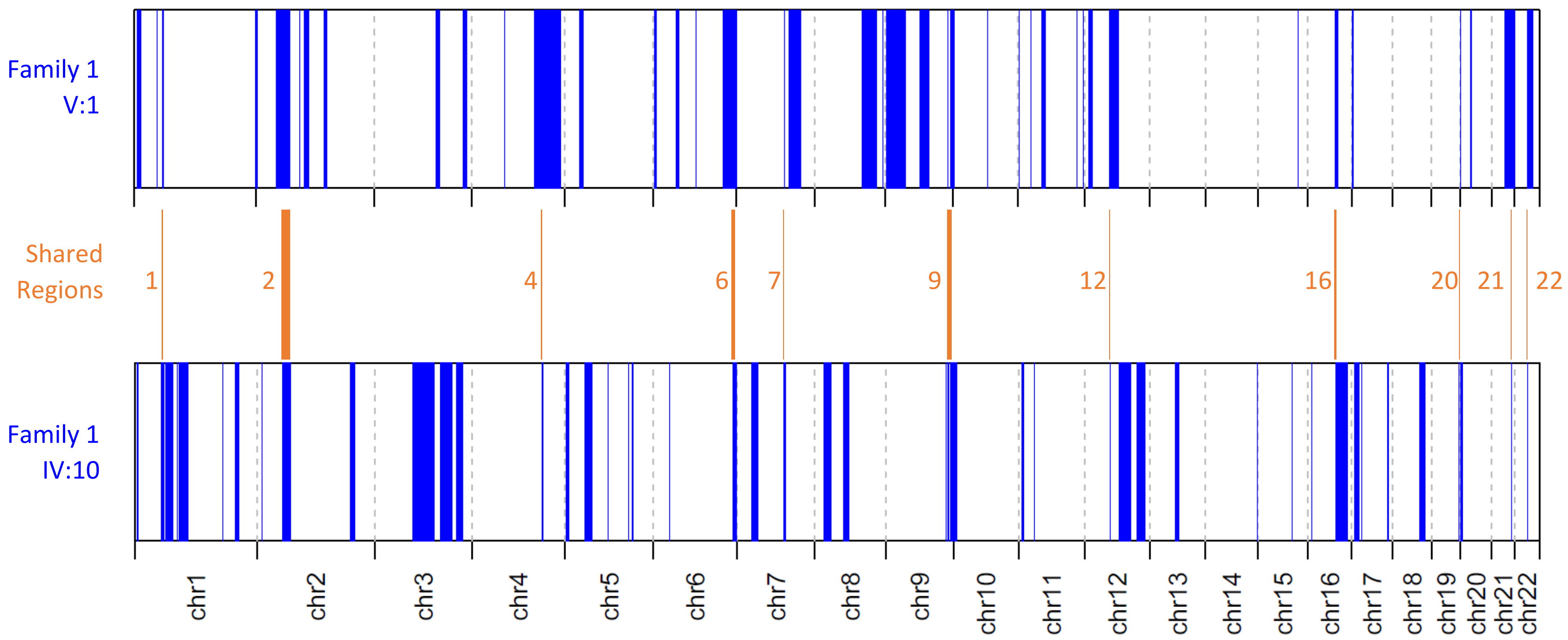

Figure S4: *CAMSAP1* sequencing chromatograms from Family 1

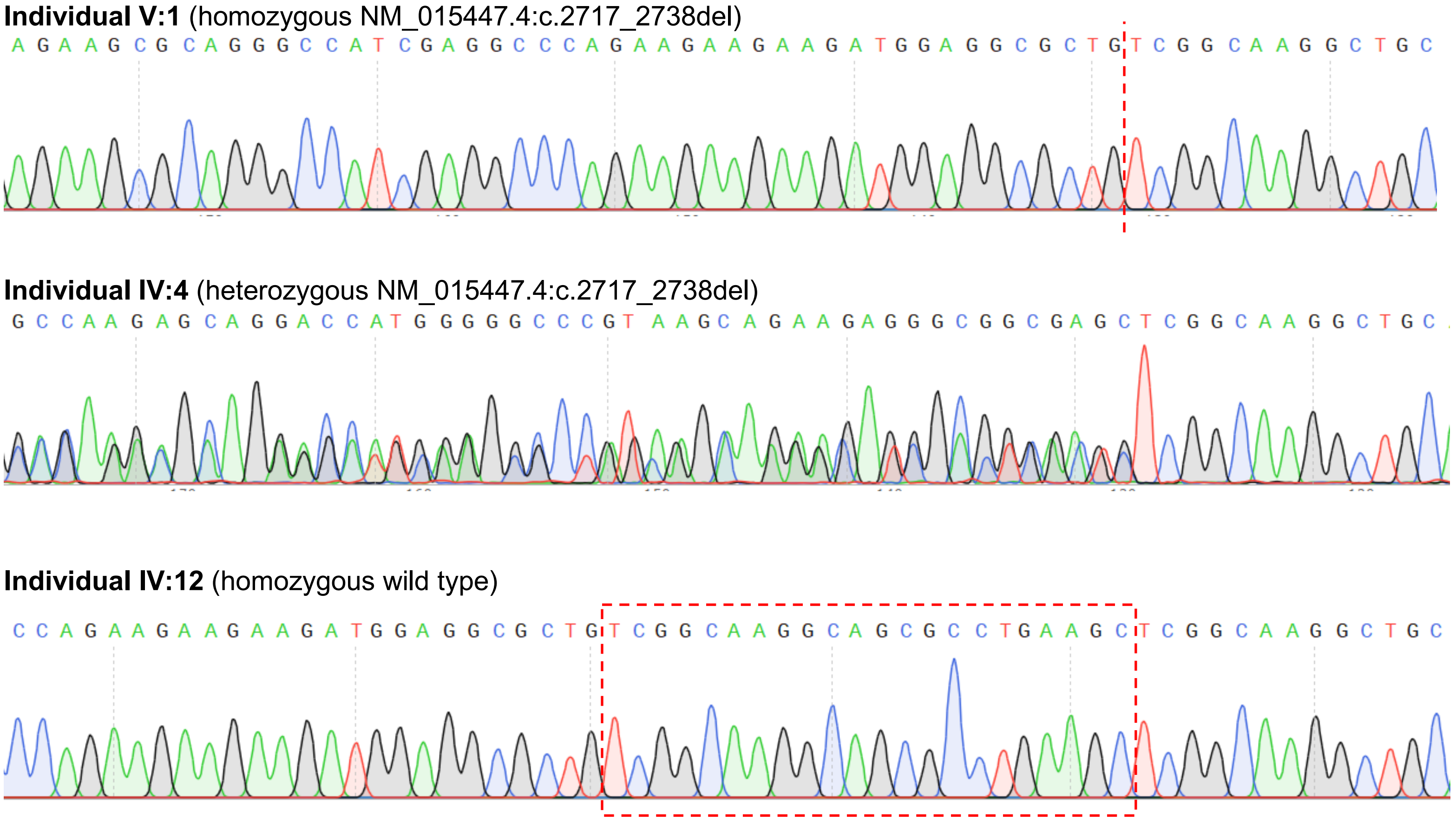

The position of the deletion is shown with a dotted red line on the chromatogram from the homozygous c.2717_2738del individual (*top*)
The 22 deleted based are shown with a red box on the chromatograms from the homozygous wild-type individual (*bottom*).

Figure S5: Recurrent *CAMSAP1* deletion may result from homologous recombination

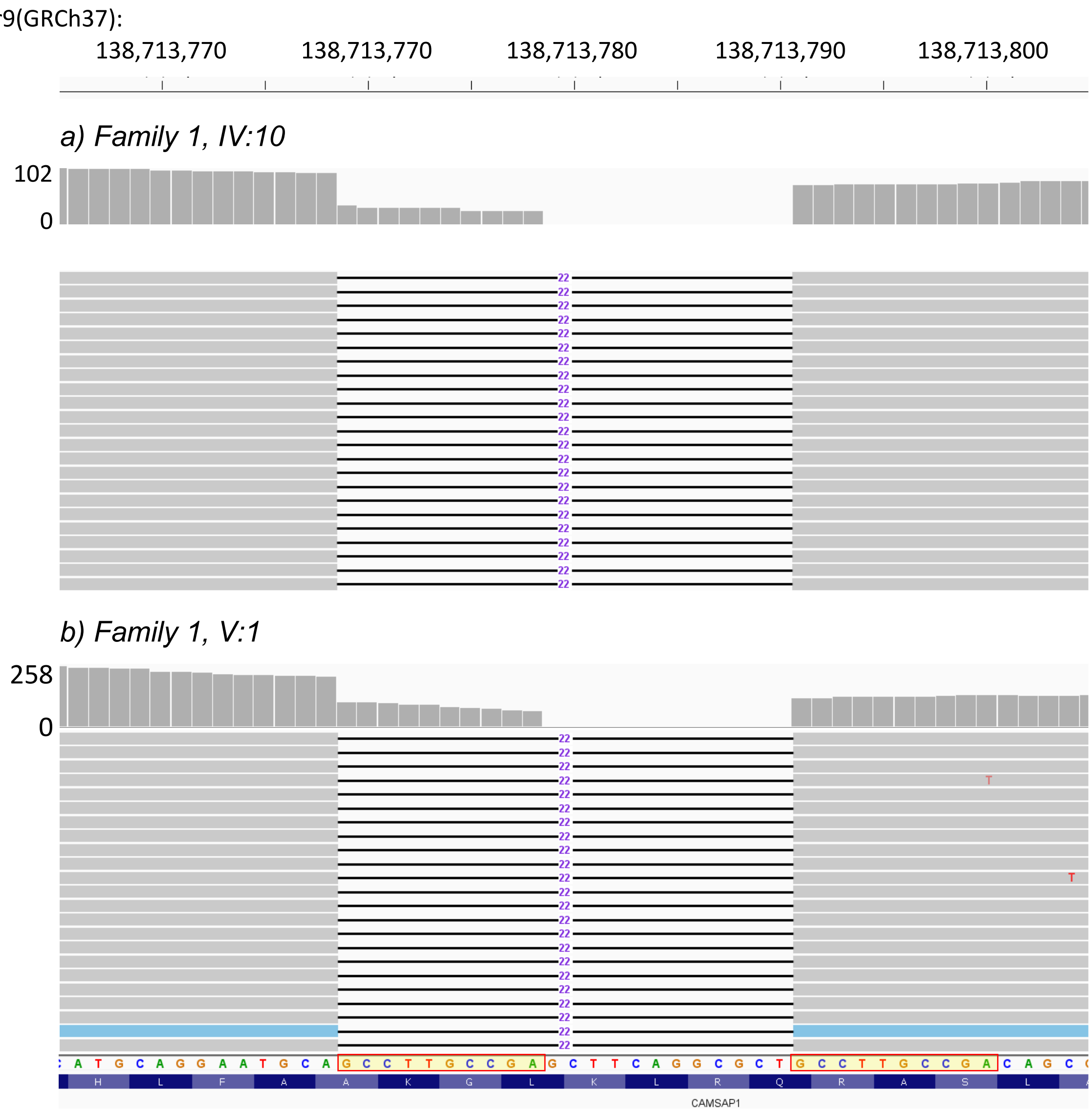

Alignment of reads generated through exome sequencing for Family 1, IV:10 and Family 1, V:1, visualized using the Integrative Genomics Viewer (version 2.9.2, Broad Institute). Position on Chromosome 9 (GRCh37) is shown at the top. Read depth is shown above a number of representative reads. The reference sequence is shown below the 22-base-pair deletion NM_015447.4:c.2717_2738del p.(Gln906Leufs*7) is shown with the homologous sequence GCCTTGCCGA highlighted in red and yellow. Recombination of these adjacent regions may be an explanation for the recurrent nature of this variant.

Figure S6: *Camsap1* genotyping and Mendelian survival

A: Conclusive genotyping example for *Camsap1* homozygous (null/null), control (wt/wt), and heterozygous (wt/null) animals. The null allele is *Camsap1*$^{em1(IMPC)J}$.

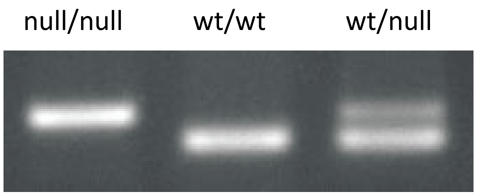

Mendelian survival tables for mice

B: **E14-E18.5:** Embryonic survival follows Mendelian ratios.

	Total	wt/wt	wt/null	null/null
Expected		12	24	12
Observed	48	9	28	11
% of total surviving	100%	19%	58%	23%

p=0.47

C: **P0-P1:** Approximately one-third of expected homozygous null animals survive birth.

	Total	wt/wt	wt/null	null/null
Expected		9	18	9
Observed	36	16	17	3
% of total surviving	100%	44%	47%	8%

p=0.087

D: P21: Homozygous null animals do not survive to weaning

	Total	wt/wt	wt/null	null/null
Expected		9.25	18.5	9.25
Observed	37	10	27	0
% of total surviving	100%	26%	69%	0%

p=0.001

Figure S7: Postnatal mutant mice do not survive in normal ratios but exhibit no skeletal abnormalities

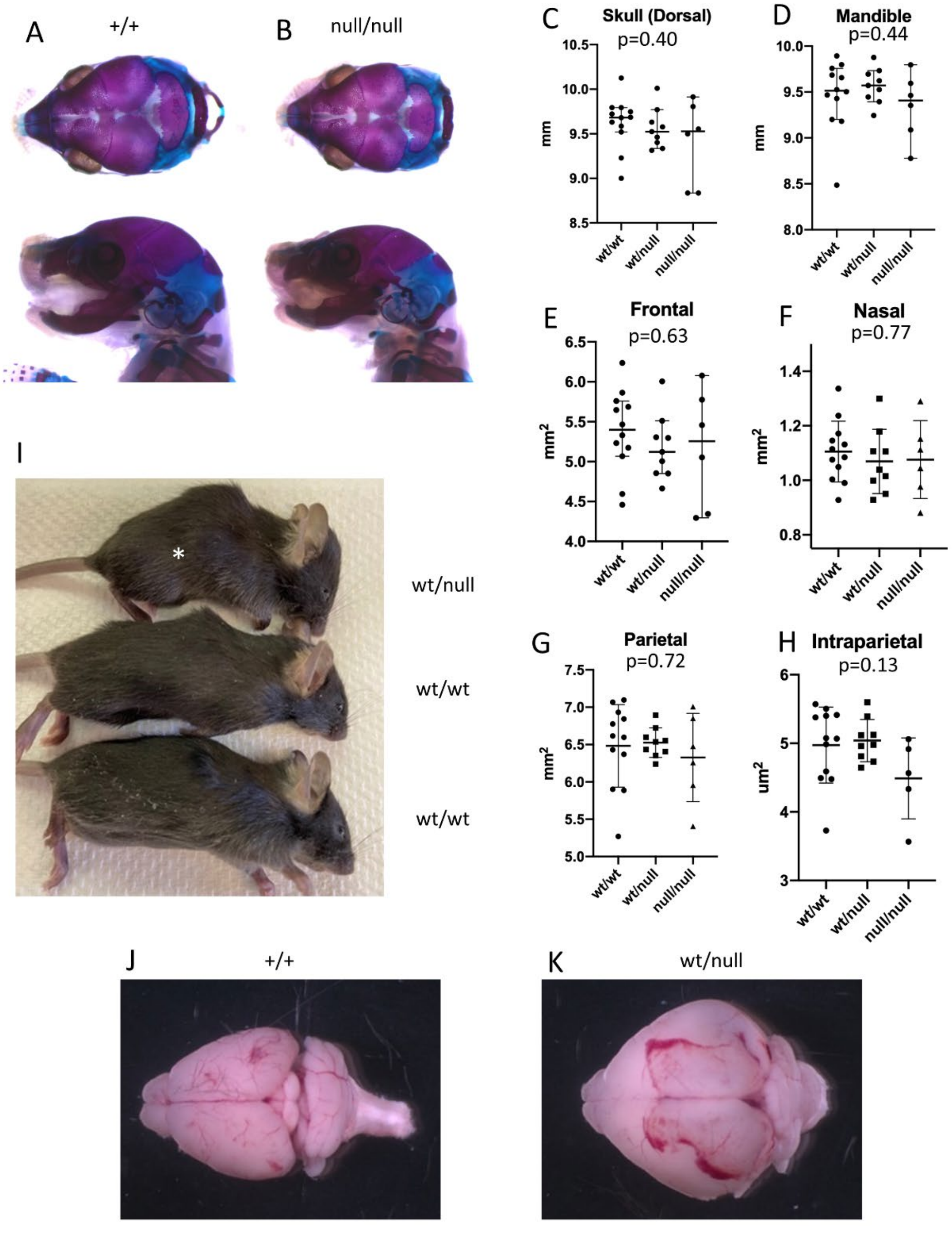

A,B - P0 skeletal preparation example images exhibiting no evident abnormalities in homozygous null animals. **C-H** - Quantification of bone length measurements from skeletal preparations (n=6-12 animals per genotype as shown in plots, Mean +/- SEM). **I** - P21 littermates, *Camsap1*$^{null/wt}$ individual with hydrocephaly marked with asterisk. **J-K** - Brains dissected from littermate control and hydrocephalic *Camsap1*$^{null/wt}$ individual. The null allele is *Camsap1*$^{em1(IMPC)J}$.

Figure S8: Embryonic *Camsap1* null mice exhibit no morphological phenotypes

Whole-mount control and *Camsap1*$^{null/null}$ embryos at stages E14.5 (**A-B**) and E16.5 (**C-D**). Hematoxylin and eosin-stained histological images of control and *Camsap1*$^{null/null}$ embryos at E14.5 (**E-F**) and E16.5 (**G-H**). No gross morphological or histological abnormalities were noted. The null allele is *Camsap1*$^{em1(IMPC)J}$.

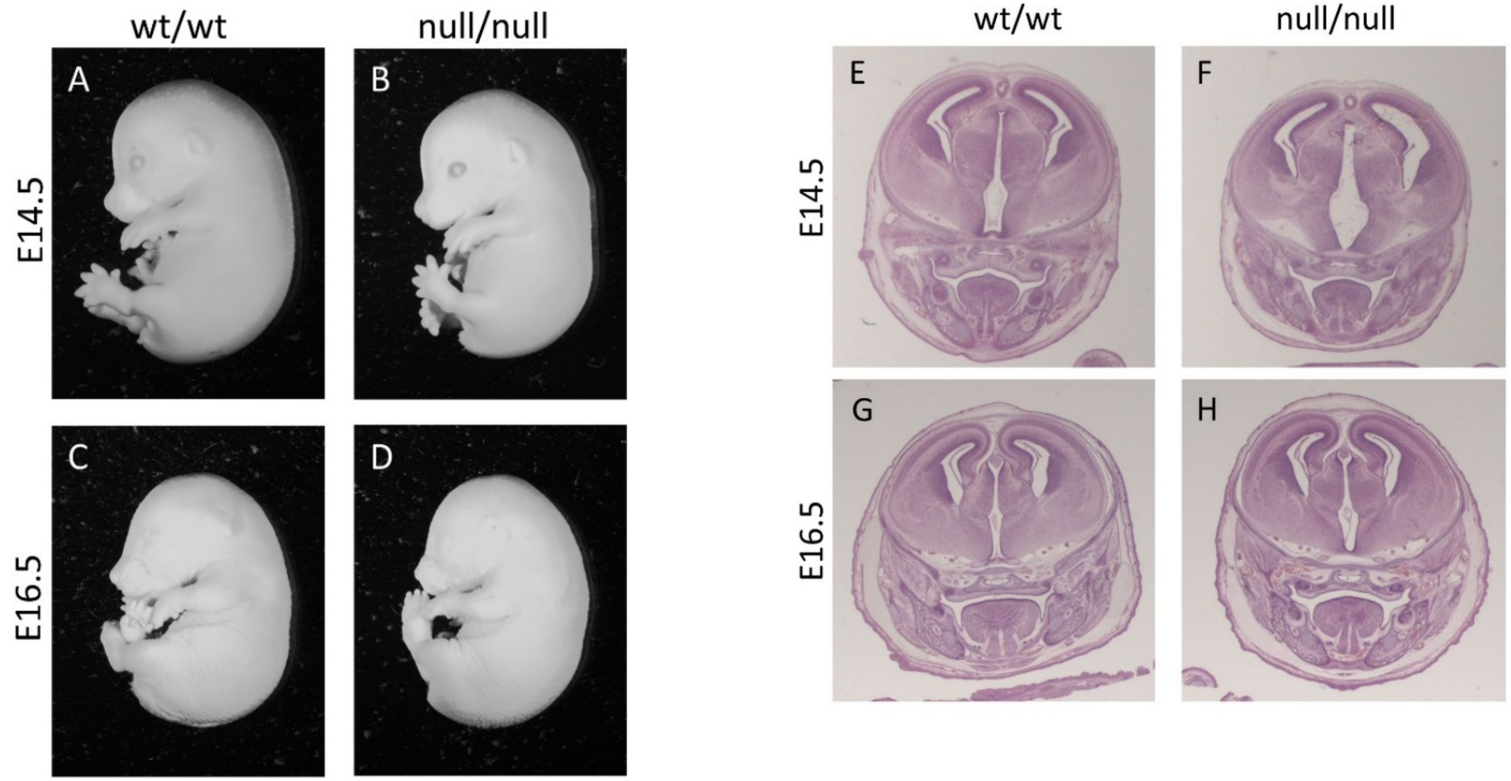

Whole-mount control and *Camsap1*$^{null/null}$ embryos at stages E14.5 (**A-B**) and E18.5 (**C-D**). Hematoxylin and eosin-stained histological images of control and *Camsap1*$^{null/null}$ embryos at E14.5 (**E-F**) and E16.5 (**G-H**). No gross morphological or histological abnormalities were noted. The null allele is *Camsap1*$^{em1(IMPC)J}$.

Figure S9: Camsap1 target region for RNA scope probe (ACDBio Cat# 866521)

Species* :	Mouse
Species (common):	House Mouse
Entrez Gene ID :	227634
Gene Alias :	9530003A05Rik
Accession No:	NM_001276359.1
Target Region :	6606 - 7920
No. of Pairs :	20

Target region as stated above:

acagt gtcccacatc ttctggcttc agggcagctg cgcatgactt ttgggttacc
6661 taggaattta tttttcatga agagttggaa gacctggtgt agagcaggaa gttgcatttc
6721 tgctcgactt ttaatacggt gacttggcat gtggcctgtc ctgaaggctg tctcagaacg
6781 cagggtctgc atgtgaagta cagtgccagt gtctcaaggc cgggctgtgg aggccatgtg
6841 gcttgatggc tcagaagcac tcgtgccttc tgtgttccat gacacagaag tagttttgat
6901 ttttttttta attaggaagt ttccggtaca ggattttgtg gtggtgatct ggagcctcca
6961 gggtgtgggt acactgtctt gaccaactgt gctgggaagg ctactcagct tttcgggcag
7021 aaagtgccaa gaaattgaat acatgacgac tcatgcagct cattccttag taacaacaga
7081 cccccccgag gagcccaggt cggttggcat taaaaatatt tctcagccgg gcggtggtgg
7141 cgcacgcctt taatccaagc acttgggagg cagagacagg cggatttctg agtttgaggc
7201 cagcctgatc tacaaagtga gttccaggac agccaggact acacagagaa accctgtctc
7261 gaaaaaataa aaaaaaaaaa tctcagaact agatgtgcag gtctacatgt ctgtgtcgat
7321 gtgggatgta cctgtgtgtg cgtatatgag tatgtgcaca caaacatgtg catatggcct
7381 gtgtacacgt gtgtatgcat gccaaagata gcgtttccag tgtgaccttt tgacctggta
7441 ttctcggtgt gttcctgcaa tacaatctcc aatcagactt ttaaggccca gactgcttca
7501 ggcaacattg aaaagtggca taatacaaaa tttacttcta gattgtttga aactatggtt
7561 gttacttgga agaagaaagt gttaaaagtc catttttctt gttgaaagtt aatcaacttg
7621 agtaaacctt tatagattgg tgctggttta cctagtgaaa tggctttgat cttggttacc
7681 tgagcctatt ggtgatttca ttctatctgt gtccagtacc acatgtgtaa agccagttct
7741 aactcctgtt ttgtgactat ggccaagtca caagccccaa ctgggaacga gccatgccca
7801 gcatcctctt gtttctcact gattccttgg caataaactt gtaccttgct gactcagggt
7861 cttcctggtg tctgaggtgc actctggagg tgcttttggt atctggtatc actggtgttc

Supplemental Tables

Table S1: Pathogenic *CAMSAP1* variants identified in this study with their frequency in population databases

CAMSAP1 variant (NM_015447.4)	Population	Chr9(GRCh38): g.	Chr9(GRCh37): g.	Nucleotide change	Exon	gnomAD v2.1.1	gnomAD v3.1.1
**c.1707dupT p.(Thr570Tyrfs*17)**	White European	135,822,954	138,714,800	insA	11/17	absent	absent
**c.1831A>T p.(Lys611*)**	Turkey	135,822,830	138,714,676	T>A	11/17	absent	absent
**c.2638C>T p.(Gln880*)**	White European	135,822,023	138,713,869	G>A	11/17	absent	absent
**c.2717_2738del p.(Gln906Leufs*7)**	Arab / White European	135,821,923_ 135,821,944	138,713,769_ 138,713,790	AGCCTTGCCGAGCTTCAGGCGCT > A	11/17	1 het	absent
**c.3130 C>T p.(Gln1044*)**	North America	135,821,531	138,713,377	G>A	11/17	absent	absent
**c.4193C>G p.(Ser1398*)**	North America	135,818,055	138,709,901	G>C	14/17	absent	absent

Abbreviations: het, heterozygous individual

Table S2: Other variants identified through exome sequencing

Individual	Variant	Zygosity, Inheritance	Gene	OMIM phenotype	Gene expression	gnomAD v2.1.1	ClinVar	SIFT	Polyphen 2	Interpretation
Family 1 V:1, IV:10 (shared)	NM_024757.4:c.623C>T; p.(Pro208Leu)	Homozygous	*EHMT1*	(AD) Kleefstra Syndrome 1; 610253	Widespread	4 het	-	0.001	0.998	Phenotype absent from parents
	NM_153710.4:c.1819G>A; p.(Asp607Asn)	Homozygous	*STKLD1*	-	Testes only	10 het	-	0.257	0.855	No phenotype, Expression only in testes
	NM_004269.3:c.107A>G; p.(Lys36Arg)	Homozygous	*MED27*	-	Esophagus, v low in brain	27 het	-	0.240	0.899	No phenotype, Low brain expression
	NM_020695.3:c.3547G>A; p.(Asp1183Asn)	Heterozygous	*REXO1*	-	Widespread	3 het	-	0.141	0.001	No phenotype, Predicted benign
	NM_020695.3:c.1493G>A; p.(Arg498His)	Heterozygous	*REXO1*	-	Widespread	13 het	-	0.022	0.955	No phenotype, Second REXO1 variant predicted benign
	NM_016252.3:c.11032A>G; p.(Ile3678Val)	Heterozygous	*BIRC6*	-	Widespread	174 het	-	0.353	0	No phenotype, predicted benign
	NM_016252.3:c.14294A>G; p.Glu4765Gly)	Heterozygous	*BIRC6*	-	Widespread	132 het	-	0.000	0.96	No phenotype, Second BIRC6 variant predicted benign
Family 2 II:1	NM_013358.2:c.592A>G; p.(Lys198Glu)	Heterozygous *de novo*	*PADI1*	-	Widespread	absent	-	0.624	0.844	No phenotype
	NM_001369.2:c.7246C>T; p.(Arg2416Cys)	Heterozygous Paternal	*DNAH5*	Ciliary dyskinesia, primary, 3, with or without situs inversus	Low in brain	2 hets	-	0.000	0.055	Phenotype absent, 2nd DNAH5 variant predicted benign
	NM_001369.2:c.4687G>A; p.(Gly1563Ser)	Heterozygous Maternal	*DNAH5*	Ciliary dyskinesia, primary, 3, with or without situs inversus	Low in brain	24 hets	Conflicting	0.54	0.002	Phenotype absent, Predicted benign
	NM_002850.3:c.1828C>G; p.(Arg610Gly)	Heterozygous Maternal	*PTPRS*	-	Widespread	1 het	-	0.36	0.081	No phenotype, Predicted benign
	NM_002850.3:c.1438G>T; p.(Val480Leu)	Heterozygous Paternal	*PTPRS*	-	Widespread	2 hets	-	0.000	0.969	No phenotype, Second PTPRS variant predicted benign
Family 3 II:1*	NM_002693.2:c.202C>T; p.(Gln68*)	Heterozygous	*POLG*	Mitochondrial DNA depletion syndrome 4A; 203700, 4B; 613622 & others	Widespread	absent	Pathogenic	-	-	Mitochondrial phenotypes are recessive, no 2nd variant
Family 4 II:1	NM_004817.4:c.2065C>T; p.(Arg689Cys)	Homozygous	*TJP2*	Cholestasis, progressive familial intrahepatic 4; 615878	Widespread	1 het	-	0.001	1	Phenotype absent in the proband
	NM_001100112.2:c.2654C>T; p.(Thr885Met)	Homozygous	*MYH2*	Proximal myopathy and ophthalmoplegia; 605637	Low in brain, high in muscle	16 het	-	0.029	0.001	Phenotype absent in the proband
	NM_014733.6:c.1670C>G; p.(Ser557Cyst)	Heterozygous *de novo*	*ZFYVE16*	-	Widespread	17 het	-	0.037	0.995	No phenotype
Family 5 II:1	NM_001099439.1:c.712G>A; p.Gly238Arg	Heterozygous	*EPHA10*	-	Low expression in cortex	3 hets for same aa	-	0.000	-	No phenotype, Low brain expression
	NM_001134479.1:c.442T>C; p.Phe148Leu	Heterozygous	*LRRC8D*	-	Widespread	absent	-	0.003	0.975	No phenotype

Individual	Variant	Zygosity, Inheritance	Gene	OMIM phenotype	Gene expression	gnomAD v2.1.1	ClinVar	SIFT	Polyphen 2	Interpretation
Family 5 II:1	NM_001531.2:c.602_603delCA; p.Thr201Argfs*47	Heterozygous	*MR1*	-	Widespread	1, multiple fs* in earlier aa	-	-	-	No phenotype
	NM_018263.4:c.1952G>C; p.Arg651Thr	Heterozygous	*ASXL2*	Shashi-Pena syndrome; 617190	Widespread	absent	-	0.000	0.942	Does not match established phenotypes
Family 5 II:1	NM_001277115.1:c.2966G>A; p.Arg989Gln	Heterozygous	*DNAH11*	Ciliary dyskinesia, primary, 7; 611884	Low expression in cortex	1 het for same amino acid	Conflicting	0.004	0.994	Does not match established phenotypes
	NM_006955.2:c.1910A>G; p.Tyr637Cys	Heterozygous	*ZNF33B*	-	Widespread	1 het	-	0.001	0.991	No phenotype
	NM_019006.3:c.579C>G; p.Ile193Met	Heterozygous	*ZFAND6*	-	Widespread	absent	-	0.002	0.991	No phenotype
	NM_018146.3:c.175delC; p.Arg59Alafs*46	Heterozygous	*MRM3*	-	Widespread	2 het for same fs*	-	-	-	No phenotype
	NM_030962.3:c.191C>T; p.Thr64Met	Homozygous	*SBF2*	Charcot-Marie-Tooth disease, type 4b2; 604563	Widespread	absent	-	0.011	0.996	Does not match established phenotypes
	NM_001039958.1: c.546_558delAGGGCAGGGGCAG; p.Gln184Argfs	Homozygous	*MESP2*	Spondylocostal dysostosis 2, autosomal recessive; 608681	Low expression in cortex	absent	-	-	-	Does not match established phenotypes
	NM_001145402.1:c.2344delG; p.Glu782Argfs*2	Homozygous	*FAM71E2*	-	Low expression in cortex	absent	-	-	-	No phenotype, Low brain expression
	NM_001145402.1:c.2333delA; p.Gln778Argfs*6	Homozygous	*FAM71E2*	-	Low expression in cortex	absent	-	-	-	No phenotype, Low brain expression
	NM_152503.5:c.93-1_93insCTTATAGACAGGGCC CCGCGGCCGGCACT; p.Asn31Lysfs*10,	Homozygous	*MROH8, RPN2*	-	Widespread	absent	-	-	-	No phenotype
	NM_032796.3:c.455G>A; p.Arg152His	Homozygous	*SYAP1*	-	Widespread	1 het	-	0.000	0.994	No phenotype

Abbreviations: aa = amino acid, fs = frameshift variant, het = heterozygous individual, hom = homozygous individual, *A full variant list was not available for Family 3, II:1. A low SIFT score suggests pathogenicity as does a high Polyphen2 score.

Table S3: Antibodies used in immunocytochemistry experiments.

Antibody Name	Concentration	Source
Cleaved Caspase-3	1:1000	Cell Signaling
Pax6	1:500	MBL
PHH3	1:500	Sigma
TUJ1	1:500	Abcam

Supplemental Methods

Clinical and genetic methods

DNA was extracted from blood/buccal samples using standard techniques. Exome sequencing was performed using DNA from individuals V:1 and IV:10 (Family 1) using either Agilent SureSelect Whole Exome v6 (Agilent Technologies, Santa Clara, CA) or Twist Human Core Exome Kit (Twist Bioscience, San Francisco, CA) exon targeting respectively. Reads were aligned (BWA-MEM v0.7.17), mate-pairs fixed and duplicates removed (Picard v2.15.0), InDel realignment/base quality recalibration (GATK v3.7.0), single-nucleotide variant (SNV) / InDel detection (GATK HaplotypeCaller), annotation (Alamut v1.8), and read depth was determined for the whole exome through our in-house pipeline. This conforms to GATK best practices. Variants were filtered based on call quality, segregation with disease, impact on gene function and allele frequency in population databases. Homozygous or compound heterozygous variants present in exons or adjacent intronic regions were evaluated and assessed for clinical correlation with phenotype.

Diagnostic exome was performed for Individual II:1 (Family 2; trio of both parents and proband) and Individual II:1 (Family 3; proband only) at GeneDx (U.S.A) using a proprietary targeting system and a custom developed analysis tool (Xome analyzer). Raw data from Individual II:1 (Family 2) was also re-analyzed using the same bioinformatic pipeline and filtering strategy as in Family 1, with the inclusion of the analysis of *de novo* variants. Trio exome was performed for Individual II:1 (Family 4) at Baylor College of Medicine as described.[5] Individual II:1 (Family 5) was analyzed using - VarSeq 2.2.3 from Golden Helix. Protein coding variants with plausible variant allele fraction (VAF - 0.3-0.7 for heterozygous variants) were selected and filtered by frequency (MAF < 0.0001, <2 individuals in gnomAD for heterozygous variants) and CADD score prediction (>25).

iPSC culture

Human induced pluripotent stem cells (iPSCs) from a control line (iPSC72.3) and from an affected individual (Family 2, II:1) were cultured in MTeSR media (STEMCELL) in Nunc plates (Fisher) on a matrix of Matrigel (CCHMC PSCF) dissolved in DMEM/F12. One clone of each line was received from the CCHMC PSCF and the Genome Engineering & Stem Cell Center, Department of Genetics, School of Medicine, Washington University in Saint Louis respectively. Cells were passaged every 7 days using Gentle Cell Dissociation Reagent (GCDR, STEMCELL) and fed daily. The STEMdiff™ SMADi Neural Induction Kit (STEMCELL) was used to generate neural rosettes from high-quality iPSC colonies. Briefly, iPSCs were dissociated into single cells and plated into an Aggrewell 800 well at 10,000 cells per well, forming embryoid bodies. These were fed daily, then replated on day 5 by filtering through a 37μM reversible strainer into a 24-well plate containing coverslips coated in Matrigel/DMEM/F12. On day 8, percent neural induction was visually estimated for each well and confirmed to be 75% or above for neural rosettes which were harvested on day 8 or day 11.

Immunocytochemistry

Cells were plated onto coverslips in a 24-well tissue culture plate and fixed in 4% paraformaldehyde for 15 minutes. To permeabilize cell membranes, coverslips were immersed in 0.1% Triton-X 100 for 5 minutes prior to blocking. Coverslips were blocked in 4% NGS for 30 mins before addition of primary antibodi(es) at 4°C overnight. Secondary antibody was applied for 1 hour, then coverslips were co-stained with DAPI for 15 min. They were sealed to glass slides with ProLong Gold Antifade Mountant. Images were acquired on a Nikon C2 Confocal Microscope. Tuj1+ (n=2), PHH3+ (n=3), and CC3+ (n=3) cells were quantified using the Brightspot Detection automated measurement function in NIS-Elements AR software. Three images were captured per coverslip per experiment, with n=2 or n=3 experimental replicates as listed above. The averages of each set of three images are shown in **Fig. 3**. Antibodies and concentrations for immunocytochemistry are listed in **Table S3**.

RNAScope

Wild-type mouse embryos maintained on a CD1 genetic background were dissected at ages E10.5, E14.5, E18.5, P7, and P27 and fixed in formalin for 16-24 h; brains were sub-dissected for ages E18.5-P27. The tissue was washed in PBS, then dehydrated and paraffin embedded by the Cincinnati Children's Hospital Medical Center (CCHMC) Pathology Core. Paraffin blocks were sectioned at 5 um, placed on SuperFrost slides, and baked at 60°C for 1 hour. Target retrieval steps outlined in the manual assay protocol were followed based on recommendations for brain tissue, then slides were dried at room temperature overnight. Hybridization and amplification steps were performed using the HybEZ oven set at 40C. Manual assay protocol from ACDBio was performed using RNAscope Multiplex Fluorescent Reagent Kit V2 (323100), TSA Cyanine 3 Fluorophores (NEL744001KT) at 1:750, and Camsap1 probe made to order by ACDBio (Cat# 866521) (**Fig. S9**).

Mouse husbandry

All animals were maintained through a protocol approved by the Cincinnati Children's Hospital Medical Center IACUC committee (IACUC2019-0068). C57BL/6NJ-*Camsap1*$^{em1(IMPC)J}$ / Mmjax mice (Jackson Labs, MMRRC Stock No. 65662-JAX) were housed in a vivarium with a 12-h light cycle with food and water *ad libitum*. Mice were maintained by intercross from the stock commercially obtained for up to three generations. Mice for dissection were euthanized with isoflurane and cervical dislocation. Whole-brain and skeletal images were taken on a Zeiss Discovery V8 microscope.

Histology

Embryos were dissected, fixed in Bouin's fixative for 48h, washed in 70% ethanol, and dehydrated and paraffin embedded by the CCHMC Pathology Core. Blocks were sectioned by microtome at 10um, then sections were placed on SuperFrost slides, baked >1 hour, and stained with hematoxylin and eosin using standard methods.

Skeletal Preparations

Pups were collected at P0-P1 and frozen. Skin and fat were removed from the embryos prior to fixation in 95% ethanol for 2-5d. The skeletons were stained with Alizarin red and Alcian blue and cleared with potassium hydroxide using standard procedures. Bone measurements were taken with Zen software and assessed for statistical significance with one-way ANOVA (n=6-12 animals per genotype).

Study approvals

Studies were conducted in accordance the declaration of Helsinki. Written informed consent was received from participants prior to inclusion in the study.

- Palestinian Health Research Council - PHRC/HC/518/19
- Cincinnati Children's Hospital Medical Center - 2014-3789
- Baylor College of Medicine - H-29697

Supplemental References

1. Wong, L.J., Naviaux, R.K., Brunetti-Pierri, N., Zhang, Q., Schmitt, E.S., Truong, C., Milone, M., Cohen, B.H., Wical, B., Ganesh, J., et al. (2008). Molecular and clinical genetics of mitochondrial diseases due to POLG mutations. Hum Mutat 29, E150-172.
2. Saneto, R.P., Lee, I.C., Koenig, M.K., Bao, X., Weng, S.W., Naviaux, R.K., and Wong, L.J. (2010). POLG DNA testing as an emerging standard of care before instituting valproic acid therapy for pediatric seizure disorders. Seizure 19, 140-146.
3. Pehlivan, D., Bayram, Y., Gunes, N., Coban Akdemir, Z., Shukla, A., Bierhals, T., Tabakci, B., Sahin, Y., Gezdirici, A., Fatih, J.M., et al. (2019). The Genomics of Arthrogryposis, a Complex Trait: Candidate Genes and Further Evidence for Oligogenic Inheritance. The American Journal of Human Genetics 105, 132-150.
4. Quinodoz, M., Peter, V.G., Bedoni, N., Royer Bertrand, B., Cisarova, K., Salmaninejad, A., Sepahi, N., Rodrigues, R., Piran, M., Mojarrad, M., et al. (2021). AutoMap is a high performance homozygosity mapping tool using next-generation sequencing data. Nature Communications 12, 518.
5. Mitani, T., Isikay, S., Gezdirici, A., Gulec, E.Y., Punetha, J., Fatih, J.M., Herman, I., Akay, G., Du, H., Calame, D.G., et al. (2021). High prevalence of multilocus pathogenic variation in neurodevelopmental disorders in the Turkish population. Am J Hum Genet 108, 1981-2005.
