# Supplementary material for: Bi-allelic CAMSAP1 variants cause a clinically recognizable neuronal migration disorder
Source: Am J Hum Genet. 2022 Oct 24;109(11):2068–79. doi: 10.1016/j.ajhg.2022.09.012 (PMC9674946; doi:10.1016/j.ajhg.2022.09.012)
Supplement: Document S2. Article plus supplemental information [file mmc2.pdf]

Bi-allelic *CAMSAP1* variants cause a clinically recognizable neuronal migration disorder

Authors

Reham Khalaf-Nazzal, James Fasham, Katherine A. Inskeep, ..., Rolf W. Stottmann, Andrew H. Crosby, Emma L. Baple

Correspondence

e.baple@exeter.ac.uk

We describe bi-allelic variants in *CAMSAP1*, which encodes a molecule crucially important for minus-end microtubule stabilization, as a cause of a clinically recognizable, syndromic neuronal migration disorder with similarities to the "tubulinopathies." *Camsap1*$^{-/-}$ mice displayed increased perinatal mortality, and proband-derived neural cell rosette lineages showed decreased cell proliferation and differentiation.

Khalaf-Nazzal et al., 2022, The American Journal of Human Genetics *109*, 2068–2079
November 3, 2022 © 2022 The Authors.
https://doi.org/10.1016/j.ajhg.2022.09.012

REPORT

Bi-allelic *CAMSAP1* variants cause a clinically recognizable neuronal migration disorder

Reham Khalaf-Nazzal,[1,26] James Fasham,[2,3,26] Katherine A. Inskeep,[4,5,26] Lauren E. Blizzard,[6] Joseph S. Leslie,[2] Matthew N. Wakeling,[2] Nishanka Ubeyratna,[2] Tadahiro Mitani,[8] Jennifer L. Griffith,[9] Wisam Baker,[10] Fida' Al-Hijawi,[11] Karen C. Keough,[12,13] Alper Gezdirici,[14] Loren Pena,[6,7] Christine G. Spaeth,[6,7] Peter D. Turnpenny,[2,3] Joseph R. Walsh,[15] Randall Ray,[16] Amber Neilson,[17] Evguenia Kouranova,[17] Xiaoxia Cui,[17] David T. Curiel,[18,19,20] Davut Pehlivan,[8,21,24] Zeynep Coban Akdemir,[8] Jennifer E. Posey,[8] James R. Lupski,[8,22,23,24] William B. Dobyns,[25] Rolf W. Stottmann,[4,5,6,7,27] Andrew H. Crosby,[2,27] and Emma L. Baple[2,3,27,*]

Summary

Non-centrosomal microtubules are essential cytoskeletal filaments that are important for neurite formation, axonal transport, and neuronal migration. They require stabilization by microtubule minus-end-targeting proteins including the CAMSAP family of molecules. Using exome sequencing on samples from five unrelated families, we show that bi-allelic *CAMSAP1* loss-of-function variants cause a clinically recognizable, syndromic neuronal migration disorder. The cardinal clinical features of the syndrome include a characteristic craniofacial appearance, primary microcephaly, severe neurodevelopmental delay, cortical visual impairment, and seizures. The neuroradiological phenotype comprises a highly recognizable combination of classic lissencephaly with a posterior more severe than anterior gradient similar to *PAFAH1B1(LIS1)*-related lissencephaly and severe hypoplasia or absence of the corpus callosum; dysplasia of the basal ganglia, hippocampus, and midbrain; and cerebellar hypodysplasia, similar to the tubulinopathies, a group of monogenic tubulin-associated disorders of cortical dysgenesis. Neural cell rosette lineages derived from affected individuals displayed findings consistent with these phenotypes, including abnormal morphology, decreased cell proliferation, and neuronal differentiation. *Camsap1*-null mice displayed increased perinatal mortality, and RNAScope studies identified high expression levels in the brain throughout neurogenesis and in facial structures, consistent with the mouse and human neurodevelopmental and craniofacial phenotypes. Together our findings confirm a fundamental role of CAMSAP1 in neuronal migration and brain development and define bi-allelic variants as a cause of a clinically distinct neurodevelopmental disorder in humans and mice.

Neuronal migration disorders arise from defects in the locomotion of neurons in the prenatal developing brain, resulting in early onset developmental impairment and seizures.[1,2] These conditions are characterized by neuroradiological and histopathological abnormalities of cortical layering, absence of normal folding (lissencephaly), agenesis of the corpus callosum, and hypo/dysgenesis of the cerebellum. A number of monogenic causes have been described, almost all of which impact the formation or functioning of microtubules.[3,4] Owing to the severity of the neurological phenotype, most cases are sporadic, resulting from *de novo* heterozygous loss-of-function variants. In published cohorts, heterozygous pathogenic variants in *PAFAH1B1* (MIM: 601545) (platelet-activating factor acetylhydrolase, isoform 1b, alpha subunit, formerly *LIS1*), which interacts with the microtubule motor

[1]Biomedical Sciences Department, Faculty of Medicine, Arab American University of Palestine, Jenin P227, Palestine; [2]Department of Clinical and Biomedical Science, University of Exeter Faculty of Health and Life Science, RILD building, Barrack Road, Exeter EX2 5DW, UK; [3]Peninsula Clinical Genetics Service, Royal Devon University Healthcare NHS Foundation Trust (Heavitree Hospital), Gladstone Road, Exeter EX1 2ED, UK; [4]Division of Developmental Biology, Cincinnati Children's Hospital Medical Center, 3333 Burnet Avenue, MLC 7016, Cincinnati, OH 45229, USA; [5]Institute for Genomic Medicine at Nationwide Children's Hospital, The Ohio State University College of Medicine, Columbus, OH 43205, USA; [6]Division of Human Genetics, Cincinnati Children's Hospital Medical Center, 3333 Burnet Avenue, MLC 7016, Cincinnati, OH 45229, USA; [7]Department of Pediatrics, University of Cincinnati College of Medicine, Cincinnati, OH 45229, USA; [8]Department of Molecular and Human Genetics, Baylor College of Medicine, Houston, TX 77030, USA; [9]Department of Neurology, Washington University School of Medicine, St. Louis, MO 63110, USA; [10]Paediatrics Department, Dr. Khalil Suleiman Government Hospital, Jenin, Palestine; [11]Paediatrics Community Outpatient Clinics, Palestinian Ministry of Health, Jenin, Palestine; [12]Department of Pediatrics, Dell Medical School, 1400 Barbara Jordan Boulevard, Austin, TX 78723, USA; [13]Child Neurology Consultants of Austin, 7940 Shoal Creek Boulevard, Suite 100, Austin, TX 78757, USA; [14]Department of Medical Genetics, Başakşehir Çam and Sakura City Hospital, 34480 Istanbul, Turkey; [15]Department of Neurological Surgery, School of Medicine, Washington University in Saint Louis, St. Louis, MO 63110, USA; [16]Departments of Pediatrics and Medical Genetics, Boston Children's Hospital and Harvard Medical School, Boston, MA 02115, USA; [17]Genome Engineering & Stem Cell Center, Department of Genetics, School of Medicine, Washington University in Saint Louis, St. Louis, MO 63110, USA; [18]Department of Biomedical Engineering, McKelvey School of Engineering, Washington University in Saint Louis, St. Louis, MO 63130, USA; [19]Division of Cancer Biology, Department of Radiation Oncology, School of Medicine, Washington University in Saint Louis, St. Louis, MO 63110, USA; [20]Biologic Therapeutics Center, Department of Radiation Oncology, School of Medicine, Washington University in Saint Louis, St. Louis, MO 63110, USA; [21]Division of Neurology and Developmental Neuroscience, Department of Pediatrics, Baylor College of Medicine, Houston, TX 77030, USA; [22]Human Genome Sequencing Center, Baylor College of Medicine, Houston, TX 77030, USA; [23]Department of Pediatrics, Baylor College of Medicine, Houston, TX 77030, USA; [24]Texas Children's Hospital, Houston, TX 77030, USA; [25]Departments of Pediatrics and Genetics, University of Minnesota, Minneapolis, MN, USA
[26]These authors contributed equally
[27]These authors contributed equally
*Correspondence: e.baple@exeter.ac.uk
https://doi.org/10.1016/j.ajhg.2022.09.012.
© 2022 The Authors. This is an open access article under the CC BY license (http://creativecommons.org/licenses/by/4.0/).

cytoplasmic dynein,[5] account for more than a third of affected individuals,[6] and hemizygous or heterozygous pathogenic variants in doublecortin (*DCX*), important for microtubule stabilization and inhibition of neurite outgrowth,[7] a further quarter.[6] Variants in several other microtubule-interacting molecules including dynein, cytoplasmic 1, heavy chain 1 (*DYNC1H1*) as well as the alpha (*TUBA1A*) (MIM: 611603), beta (*TUBB2A*, *TUBB2B*, *TUBB3*, *TUBB4A*, *TUBB*) (MIM: 615763, 610031, 614039, 612438, 615771), and gamma-tubulin (*TUBG1*) (MIM: 615412) subunits are also well-recognized genetic causes of neuronal migration and brain malformation disorders.[4,8] Those conditions caused by pathogenic variants in tubulin subunits are collectively termed the "tubulinopathies"[9] and display distinctive neuroradiological features, which in addition to the cortical layering abnormalities, include hypoplasia/aplasia of the corpus callosum, hypoplasia of the oculomotor and optic nerves, cerebellar hypodysplasia (including foliar dysplasia), and dysmorphism of the basal ganglia and hind-brain structures.[10]

The CAMSAP (calmodulin-regulated spectrin-associated protein) family contains three human proteins (CAMSAP1, CAMSAP2 [CAMSAP1L1/KIAA1078] and CAMSAP3 [KIAA1543]) essential for the formation and maintenance of the minus end of non-centrosomal microtubules.[11] At least one orthologue exists in all eumetazoans, including the well-described *Patronin* in *Drosophila*, indicating a fundamental requirement for at least one CAMSAP molecule in mitotic processes.[12,13] CAMSAP proteins are defined by a highly conserved "CKK" (CAMSAP1, KIAA1078/CAMSAP2, KIAA1543/CAMSAP3) domain at the C terminus[12] with an additional 5′ calponin homology (CH) domain and intermediate coiled-coil (CC) motif also invariably present.[14] The CKK domain, predicted to adopt a β-barrel conformation with a single invariant tryptophan residue within its core, directs each CAMSAP protein to the microtubule minus end. CAMSAP1 binds transiently to the outermost microtubule ends,[15] stabilizing them without affecting tubulin incorporation rate *in vitro*.[11,16,17] Conversely, CAMSAP2 and CAMSAP3 remain bound to and decorate the microtubule lattice, exerting a stabilizing effect and reducing tubulin incorporation rates.[11,17,18] A recently described *Camsap1* knockout mouse model (*Camsap1*$^{-/-}$) manifested preweaning lethality with epileptic seizures and abnormal cortical lamination, highly suggestive of impaired neuronal migration.[19] Neurons from *Camsap1*$^{-/-}$ mice displayed abnormal polarization resulting in a multi-axon phenotype. To date however, no human monogenic disorder has been associated with any of the *CAMSAP* genes. Here, we define bi-allelic *CAMSAP1* (MIM: 613774) variants as a cause of a clinically and radiologically distinct syndromic neuronal migration disorder.

We initially identified an extended Palestinian kinship comprising of two interlinking nuclear families with three children aged between 4 months and 3 years 9 months (family 1, V:1, 1V:10, and IV:11, pedigree is shown in Figure 1), affected by a syndromic neuronal migration disorder (recruited with informed consent and Palestinian Health Research Council PHRC/51819 ethical approval). The three children presented with severe primary microcephaly (−4.8 to −6.4 SDS [standard deviation score]), profound global developmental impairment, and craniofacial dysmorphism including large ears, high palate, metopic ridging, and a flat wide nasal bridge (Figure S1). Neurological examination findings included central hypotonia and peripheral hypertonia with brisk reflexes and positive Babinski sign bilaterally. All three affected individuals developed seizures at an early age, which have been refractory to treatment, and the two older children (V:1 and IV:10) have a diagnosis of cortical visual impairment. MRI neuroimaging findings in all three children were consistent and include agyria/severe pachygyria with a posterior greater than anterior gradient, dysmorphic basal ganglia, and absent corpus callosum (Figures 2A–2D for family 1, V:1, Figures S2A–S2D for family 1, IV:11).

To define the genetic cause of disease, exome sequencing was undertaken with DNA from individuals IV:10 and V:1 (Illumina HiSeq and Twist Human Core Exome Kit), assuming homozygosity for a founder variant, although also considering other inheritance mechanisms. SNVs and indels were detected with GATK HaplotypeCaller and annotated with Alamut batch (v.1.8). Copy-number variants were detected with both SavvyCNV[20] and ExomeDepth (https://github.com/vplagnol/ExomeDepth). Variants failing quality filters or present at a frequency of >0.1% or with >1 homozygous individual in gnomAD (v.2.1.1 or v.3.1.1) or in our in-house database were excluded. Homozygous and compound heterozygous variants common to both affected individuals and present in exons or within ±6 nucleotides in the intron were then evaluated (a full description of the exome bioinformatic pipeline is included in the supplemental methods). A single standout candidate cause of the phenotype was identified, a homozygous variant common to both individuals, Chr9(GRCh38): g.135821923_135821944del (GenBank: NM_015447.4) (*CAMSAP1*) (c.2717_2738del; p.Gln906Leufs*7), located within a ~8Mb region of homozygosity shared between IV:10 and V:1 [Chr9(GRCh38): g.130299324 to the 9q terminus] (Figure S3). The variant was confirmed by dideoxy sequencing (Figure S4) and found to cosegregate as expected for an autosomal recessive disease in the third affected child (IV:11), parents, and three unaffected siblings. The variant, located in exon 11/17, is predicted to cause a frameshift and premature termination, most likely resulting in nonsense-mediated decay and bi-allelic loss of function.

Through international collaboration (including Gene Matcher),[21] we identified four additional affected children from four unrelated families, in whom exome sequencing undertaken on Illumina platforms identified bi-allelic predicted loss-of-function *CAMSAP1* variants. These individuals (aged 1–6 years) presented with clinical and neuroradiological features overlapping those of the Palestinian children. Clinical data were obtained with informed

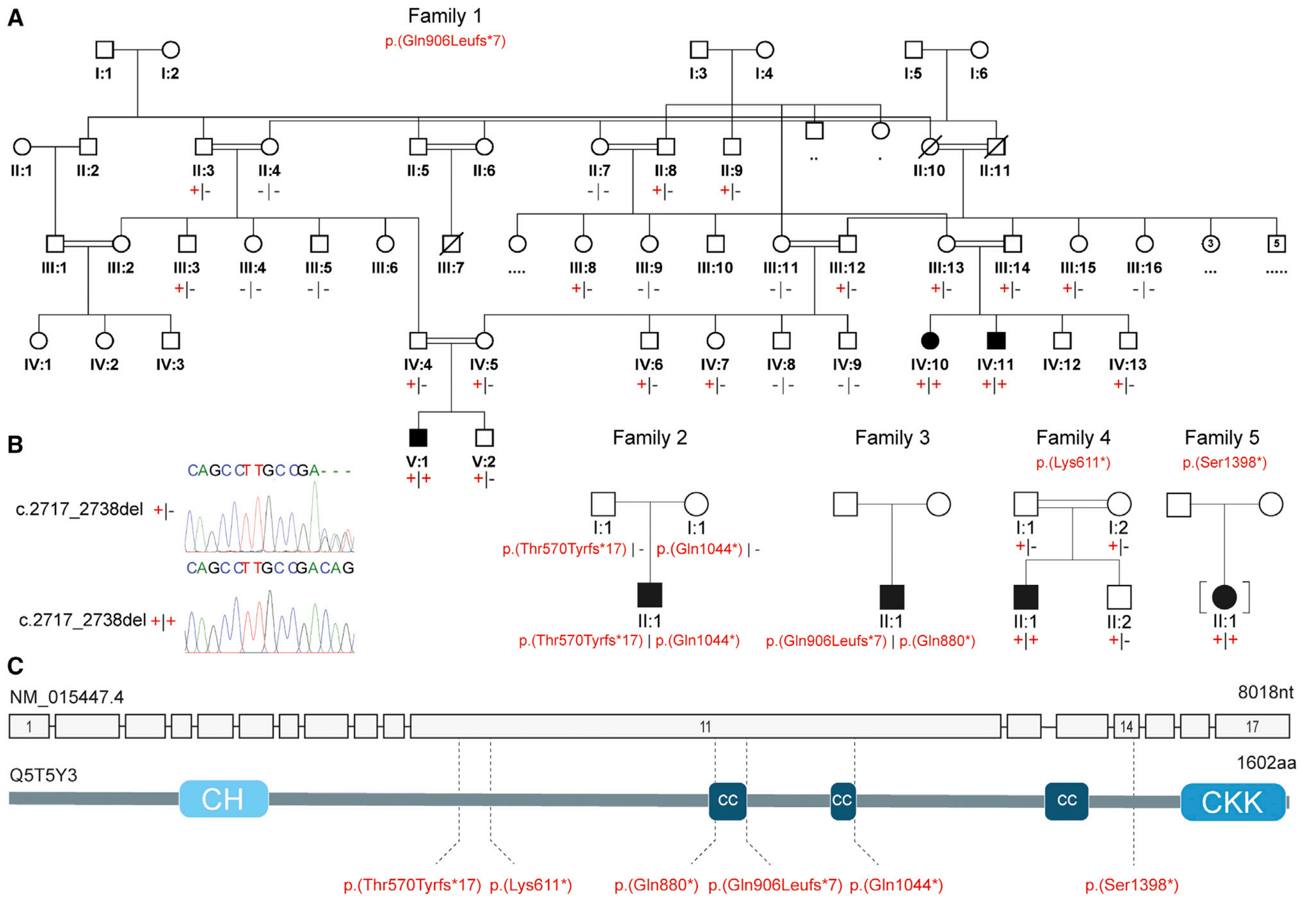

Figure 1. Family pedigrees and bi-allelic *CAMSAP1* variants associated with a syndromic neuronal migration disorder
(A) Simplified pedigrees of families in the study showing cosegregation of the variants identified ("–" wild-type allele; "+" familial variant), with reference to transcript GenBank: NM_015447.4.
(B) Chromatogram for the c.2717_2738 deletion is shown with heterozygous (top) and homozygous variant (bottom) individuals shown.
(C) Intron/exon genomic organization of *CAMSAP1* (top) and protein domain architecture of CAMSAP1 (bottom) illustrating the calponin homology (CH), coiled coil (CC), and calmodulin-regulated spectrin-associated (CKK [CAMSAP1, KIAA1078/CAMSAP2, KIAA1543/CAMSAP3] domain) domains alongside the location of each of the identified pathogenic variants (dotted line).

consent by local clinicians using a standardized proforma. Clinical findings on the seven affected individuals are summarized in Table 1. The pedigrees and clinical photos depicting morphological features are shown in Figures 1 and S1. Detailed clinical descriptions of all individuals are provided in the supplemental note (case reports). Retrospective analysis of neuroimaging findings (5/5 where complete data were available) was undertaken by author W.B.D. and is shown in Figures 2 and S2. The *CAMSAP1* variants and exome variants identified in each family in this study are listed in Tables S1 and S2, respectively.

Family 2, II:1 is the eldest child of unaffected, unrelated North American parents of North European ancestry. Antenatal brain imaging showed lissencephaly, agenesis of the corpus callosum (aCC), and a small cerebellum. He was hypotonic at birth and exhibited early feeding difficulties. From 4 months, he was affected by infantile spasms and tonic seizures and his EEG showed a modified hypsarrhythmia pattern. At age 5 years, he has severe global developmental delay (GDD), is unable to sit unsupported, and is non-verbal but uses a gaze tracking device to indicate his needs. Neurological findings include axial hypotonia, peripheral hypertonia, and cortical visual impairment. He has a similar facial gestalt (prominent metopic suture, wide nasal bridge, and prominent cupids bow) to the affected Palestinian children (Figures S1E and S1F). MRI revealed diffuse severe pachygyria with a "posterior more severe than anterior" (P > A) gradient, dysmorphic basal ganglia, absent corpus callosum, and enlarged posterior fossa or "mega cisterna magna" (Figures 2E–2H). He underwent diagnostic trio-exome sequencing through GeneDx (USA), which identified *in trans* compound heterozygous predicted loss-of-function *CAMSAP1* variants [Chr9(GRCh38): g.135822954dupA (GenBank: NM_015447.4) (c.1707dupT; p.Thr570Tyrfs*17) and Chr9(GRCh38): g.135821531G>A (GenBank: NM_015447.4) (c.3130C>T; p.Gln1044*)]. To exclude other potential causes of disease, the exome data were then reanalyzed with the same bioinformatic pipeline and filtering strategy applied to the exome data from family 1 (variant list – Table S2).

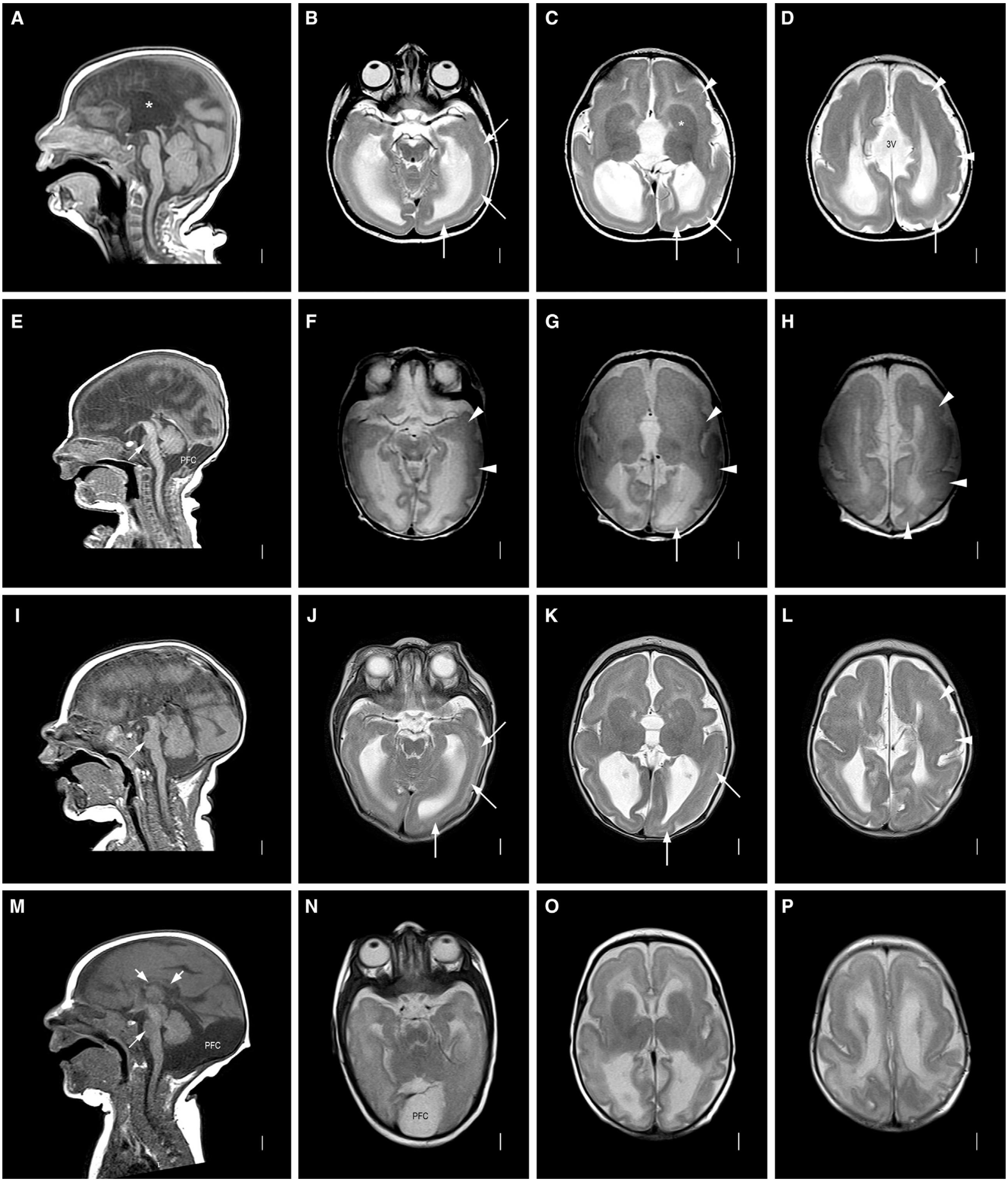

Figure 2. Neuroradiology of affected individuals
Neuroimaging in four individuals with the *CAMSAP1*-related neuronal migration disorder (for further imaging see Figure S2): row 1 (A-D) is family 1, V:1 aged 7 months; row 2 (E-H) is family 2, II:1 aged 2 days; row 3 (I-L) is family 3, II:1 aged 3 months; row 4 (M-P) is family 4, II:1 aged 3 months. T1-weighted midline sagittal images show absent (* in A, also E and I) or short and thin (short white arrows in M) corpus callosum and small base of the pons (thin white arrow in E, I, and M). Enlarged posterior fossa or "mega cisterna magna" (PFC in E and M) was seen in 2/4 subjects. T2-weighted axial images show posterior-more-severe-than-anterior gradient with areas of agyria or severe pachygyria with prominent cell sparse zones and reduced thickness of the cerebral mantle/wall in posterior regions (white arrows in B–D, G, J, and K) and areas of less severe pachygyria with thicker cerebral mantle/wall in anterior regions (white arrowheads in C, D, F–H, and L). The gradient in family 4, II:1 (N–P) was less clear with lower resolution images. The boundaries of the basal ganglia and thalami were difficult to see, and the internal capsules are not seen (* in C, also in G, K, and O). The third ventricle was enlarged in all and dramatically enlarged into a midline interhemispheric cyst in family 1, V:1 (3V in D).

Table 1. Summary of clinical and neurological features of individuals with *CAMSAP1*-related neuronal migration disorder

Individual	Family 1, V:1	Family 1, IV:10	Family 1, IV:11	Family 2, II:1	Family 3, II:1	Family 4, II:1	Family 5, II:1
CAMSAP1 variants (GenBank: NM_015447.4)	homozygous c.2717_2738del (p.Gln906Leufs*7)	homozygous c.2717_2738del (p.Gln906Leufs*7)	homozygous c.2717_2738del (p.Gln906Leufs*7)	c.1707dupT (p.Thr570Tyrfs*17); c.3130C>T (p.Gln1044*)	c.2717_2738del (p.Gln906Leufs*7); c.2638C>T (p.Gln880*)	homozygous c.1831A>T (p.Lys611*)	homozygous c.4193C>G (p.Ser1398*)
Region	Palestine	Palestine	Palestine	North America	North America	Turkey	North America
Sex	M	F	M	M	M	M	F
**Growth**							
Measurement age (y)	3 y	3.8 y	0.3 y	3.6 y	1.1 y	6.4 y	4.8 y
Birth OFC cm (SDS)	microcephaly	microcephaly (1 m)	microcephaly	microcephaly	microcephaly (4 m)	32 (−2.5)	31.8 (−2.2)
Height cm (SDS)	NK	NK	NK	99.1 cm (−0.2)	66 cm (−4.2)	125 cm (+1.3)	106.5 cm (−0.1)
Weight kg (SDS)	NK	NK	NK	13.5 kg (−1.4)	7.7 kg (−14.5)	25 kg (+1.0)	17.3 kg (−0.2)
OFC cm (SDS)	42.7 cm (−4.8)	40.7 cm (−6.4)	microcephaly	45 cm (−4.8)	42 cm (−5.0)	53 cm (−0.2)	42.5 cm (−7.1) OFC at 3.8 y
**Neurology**							
Age at assessment (y)	3 y	3.8 y	0.3 y	5 y	1.7 y	5.5 y	4.8 y
Global dev. delay	profound	profound	profound	severe	severe	severe	severe
Central tone	↓	↓	↓	↓	↓	↓ (severe)	↓
Peripheral tone	↑	↑	NK	↑	↓	↑	↑
Deep tendon reflexes	+++	+++	NK	++; brisk	++	+++	+++
Plantar reflexes	↑	↑	NK	NK	absent	NK	NK
Seizures/age of onset	from 1 m	–	from 2 m	from 4 to 5 m	from 5 m	from 5 m	from 2 m
EEG findings	NK	NK	NK	modified hypsarrhythmia	hypsarrhythmia	burst suppression	multifocal epileptiform discharges
Cortical visual impairment	+	+	NK	+	+	+	+
Feeding difficulties	+	+	+	+	gastrostomy	+	gastrostomy
**Facial features**							
Prominent metopic suture	+	+	+	+	+	–	–
Wide nasal bridge	+	+	+	+	+	+	+
Pronounced cupids bow	+	+	+	+	+	–	+
Large prominent ears	+	+	+	+	+	+	–
High arched palate	+	+	NK	–	NK	+	+

(*Continued on next page*)

Table 1. *Continued*

Individual	Family 1, V:1	Family 1, IV:10	Family 1, IV:11	Family 2, II:1	Family 3, II:1	Family 4, II:1	Family 5, II:1
**Neuroimaging**							
Lissencephaly/pachygyria	+	+	+	+	+	+	+
ACC/severe HCC	+	+	+	+	+	+	+
Dysplastic basal ganglia	+	+	+	+	+	+	+
Enlarged posterior fossa	–	–	–	+	–	+	–
Cerebellar hypoplasia	mild	mild	+	+	+	+	mild
Other clinical features	hyperopia with astigmatism, clinodactyly	clinodactyly	none noted	cryptorchidism, scoliosis	none noted	cryptorchidism, femoral hernia	deceased age 5.5 y

Abbreviations: +, feature is present; –, feature is absent; ↑, increased; ↓, decreased; +++, hyperactive; aCC, agenesis of the corpus callosum; cm, centimeters; dev., developmental; DWS, Dandy-Walker syndrome; F, female; hCC, hypogenesis of the corpus callosum; m, months; M, male; NK, not known; OFC, occipitofrontal circumference; SDS, standard deviation score; y, years.

Family 3, II:1, a 20-month-old male child born to North American parents of Northern European ancestry, was noted to be microcephalic and display abnormal movements in early infancy. His EEG showed hypsarrhythmia and he was diagnosed with infantile spasms at 5 months of age. His seizures have progressed, requiring polytherapy for effective control. He has severe GDD (crawling, nonverbal, reaching for objects), cortical visual impairment, and generalized hypotonia. He has a history of feeding difficulties requiring nasogastric feeding and parenteral gastrostomy (PEG) placement for an unsafe swallow. His craniofacial features are similar to those of the other affected children. Brain MRI (Figures 2I–2L) revealed pachygyria with thicker cerebral mantle anteriorly, enlarged 3rd ventricle, and dysmorphic basal ganglia and thalami with internal capsule not seen. Singleton diagnostic exome sequencing undertaken through GeneDx (USA) identified compound heterozygous *in trans* predicted loss-of-function variants in *CAMSAP1*, including the same frameshift variant identified in family 1 [Chr9(GRCh38): g.135821923_135821944del (GenBank: NM_015447.4) (c.2717_2738del; p.Gln906Leufs*7)] and a nonsense variant, Chr9(GRCh38): g.135822023G>A (GenBank: NM_015447.4) (c.2638C>T; p.Gln880*).

Family 4, II:1 is a six-year-old child of related Turkish parents, investigated as part of a large cohort study to define candidate genetic causes of neurodevelopmental disorder.[22] He was found to be microcephalic at birth (−2.5 SDS) and was diagnosed with infantile spasms aged 5 months, before going on to develop multiple other seizure types. His EEG showed a burst-suppression pattern. He has mild craniofacial dysmorphism, profound GDD, central hypotonia, limb spasticity, epilepsy, and cortical visual impairment. Limb movements are described as dyskinetic with varying spasticity of his limbs and intermittent guarded rigidity. MRI revealed diffuse lissencephaly, dysmorphic basal ganglia, a thin corpus callosum and enlarged posterior fossa or "mega cisterna magna" (Figures 2M–2P). Trio-exome sequencing performed at Baylor College of Medicine (USA), as previously described,[22] identified a candidate homozygous nonsense variant, Chr9(GRCh38): g.135822830T>A (GenBank: NM_015447.4) (c.1831A>T; p.Lys611*), located within a 4.2 Mb region of homozygosity.

Family 5, II:1 is a 4-year-old adopted child, with profound GDD, generalized seizures and severe microcephaly (−7.1 SDS). Neurological examination revealed central hypotonia with bilateral lower limb spasticity and dystonic movements, with craniofacial features including a wide nasal bridge and pronounced cupids bow (Figure S1H). EEG findings of multifocal epileptiform discharges were consistent with electroclinical seizures that appeared to lateralize to either hemisphere. MRI findings include holohemispheric bilateral lissencephaly and grey matter band heterotopia with notable white matter volume loss, a prominent cisterna magna, and diffusely small brainstem with decreased volume of the dorsal pons (Figures S2E

and S2F). Proband-only exome sequencing performed at Cincinnati Children's Hospital Medical Center (CCHMC) with previously described methodology[23] identified a homozygous *CAMSAP1* variant [Chr9: g.135818055G>C (GenBank: NM _015447.4) (c.4193C>G; p.Ser1398*)].

All the *CAMSAP1* variants identified as part of this study were predicted to result in nonsense mediated mRNA decay and loss of function. The variants were predominantly (5/6) located in the largest exon of the *CAMSAP1* gene (exon 11/17, Figure 1B) and are absent from the genome aggregation database (gnomAD v.2.1.1 and v.3.1.1), with the exception of p.Gln906Leufs*7, present in two unrelated families in this study and one additional heterozygous Finnish individual in gnomAD (Table S1). Further inspection of the genomic architecture surrounding this variant suggests its recurrent nature may result from a homologous recombination event (Figure S5). Furthermore, there are no homozygous loss-of-function *CAMSAP1* variants listed in publicly accessible genomic databases. The exome filtering steps followed in families 3–5 were similar to those described for families 1 and 2 and are detailed in the supplemental methods. In all families, the *CAMSAP1* variants cosegregated as expected for an autosomal recessive trait (Figure 1). In family 3, the closely collocated compound heterozygous variants in *CAMSAP1* (p.Gln906Leufs*7 and p.Gln880*) could be determined to be *in trans* by phasing the short-read exome sequencing data.

We next investigated the functional consequences of the *CAMSAP1* variants in induced pluripotent stem cells (iPSCs). Peripheral blood mononuclear cells (PBMCs) were isolated from whole blood from an affected individual (family 2, II:1), cultured to enrich erythroid progenitor cells, which were transduced with a Sendai viral cocktail that expresses the Yamanaka factors, Klf4, cMyc, Oct4, and Sox2, to obtain iPSCs. The *CAMSAP1* genotype of the iPSC line was confirmed by next-generation sequencing. These iPSCs were used alongside an iPSC wild-type control cell line (iPSC72.3, CCHMC PSCF) to generate neural rosettes: radially organized two-dimensional structures of neural progenitor cells (NPCs) and differentiating neurons. We analyzed the neural rosettes at both 8 days *in vitro*, when they are entirely composed of progenitor cells, and 11 days *in vitro*, when the progenitors begin to produce differentiating neurons along the outer edge of each rosette. The morphology of rosettes derived from the affected child (family 2, II:2) were abnormal, showing large clusters of cells improperly collecting in the center of each rosette (Figures 3A and 3B). We stained rosettes for PAX6, a marker of neuronal progenitors, and TUJ1 which marks differentiated neurons. These data demonstrated significantly fewer differentiated neurons present at 11 days *in vitro* in rosettes from the affected individual (Figures 3C–3H). Additionally, a proliferation defect was evident by the reduced number of pHH3+ cells present at both 8 and 11 days (Figures 3I–3J). Finally, cleaved caspase-3 (CC3) is upregulated in disease-associated rosettes, indicating an increased level of cell death, which may explain the presence of the dense cell clusters previously noted (Figures 3K–3N). Overall, iPSCs and neural rosettes derived from the affected child with the *CAMSAP1*-related neuronal migration disorder show increased apoptosis, and decreased proliferation and differentiation of neuronal progenitor cells, consistent with the neuronal migration defects observed in affected individuals.

Despite the availability of RNA sequencing (RNA-seq) datasets describing general expression of the *CAMSAP* genes in mouse and human tissues including during brain development, no detailed temporospatial expression studies of *CAMSAP1* in the developing embryo have been performed. This is relevant given the craniofacial and specific central nervous system (CNS) abnormalities associated with this condition. We thus performed RNAScope *in situ* RNA hybridization in the developing mouse at several stages from embryonic day 10.5 (E10.5) to adulthood (postnatal day 27). At E10.5, *Camsap1* is highly and ubiquitously expressed in the head, particularly in the brain and throughout the first pharyngeal arch, and neural tube (Figures 4A and 4B). At E14.5 or mid-neurogenesis, expression in the brain becomes slightly more localized to the ganglionic eminences and cortex (Figure 4C) and present (albeit somewhat reduced) in the caudal neural tube (Figure 4D). At late stages of neurogenesis (E18.5), *Camsap1* in the brain is expressed most highly in the cortex, particularly in upper layers of differentiated neurons and the ventricular zone (Figures 4E and 4F). Postnatally, *Camsap1* is particularly evident in upper cortical layers and in the hippocampus (Figures 4G–4J). High embryonic expression in the developing face tissues and cortex from E10.5–18.5 would be consistent with craniofacial abnormalities and CNS malformations such as microcephaly and lissencephaly, which we observe in individuals with *CAMSAP1*-related neuronal migration disorder. Postnatal expression in upper cortical layers correlates with previous mouse and rat expression data indicating that *Camsap1* is expressed in specific populations of neurons and astrocytes.[24]

Although previous studies of *Camsap1* knockout mice were robust,[19] they focused on the laminar organization of the cortex and neuronal polarity and not craniofacial phenotypes. We thus obtained a null allele of *Camsap1* (*Camsap1*$^{em1(IMPC)J}$; hereafter referred to as the "null" allele) in order to investigate the wider effects of CAMSAP1 loss in the mouse. Homozygous null mice were examined at birth (P0) and during weaning (P21) (see Figure S6A for conclusive genotyping), revealing that while we observe normal Mendelian survival of *Camsap1*$^{null/null}$ animals at embryonic stages E14.5–E18.5 (Figure S6B), *Camsap1*$^{null/null}$ animals do not survive in Mendelian ratios postnatally, with only approximately one-third of expected homozygous null animals present at P1 (Figure S6C, n = 3 of an expected 9) and zero of an expected ten at P21 (Figure S6D). We examined surviving *Camsap1*$^{null/null}$ animals, finding no overt morphological or skeletal abnormalities (Figures S7A–S7K).

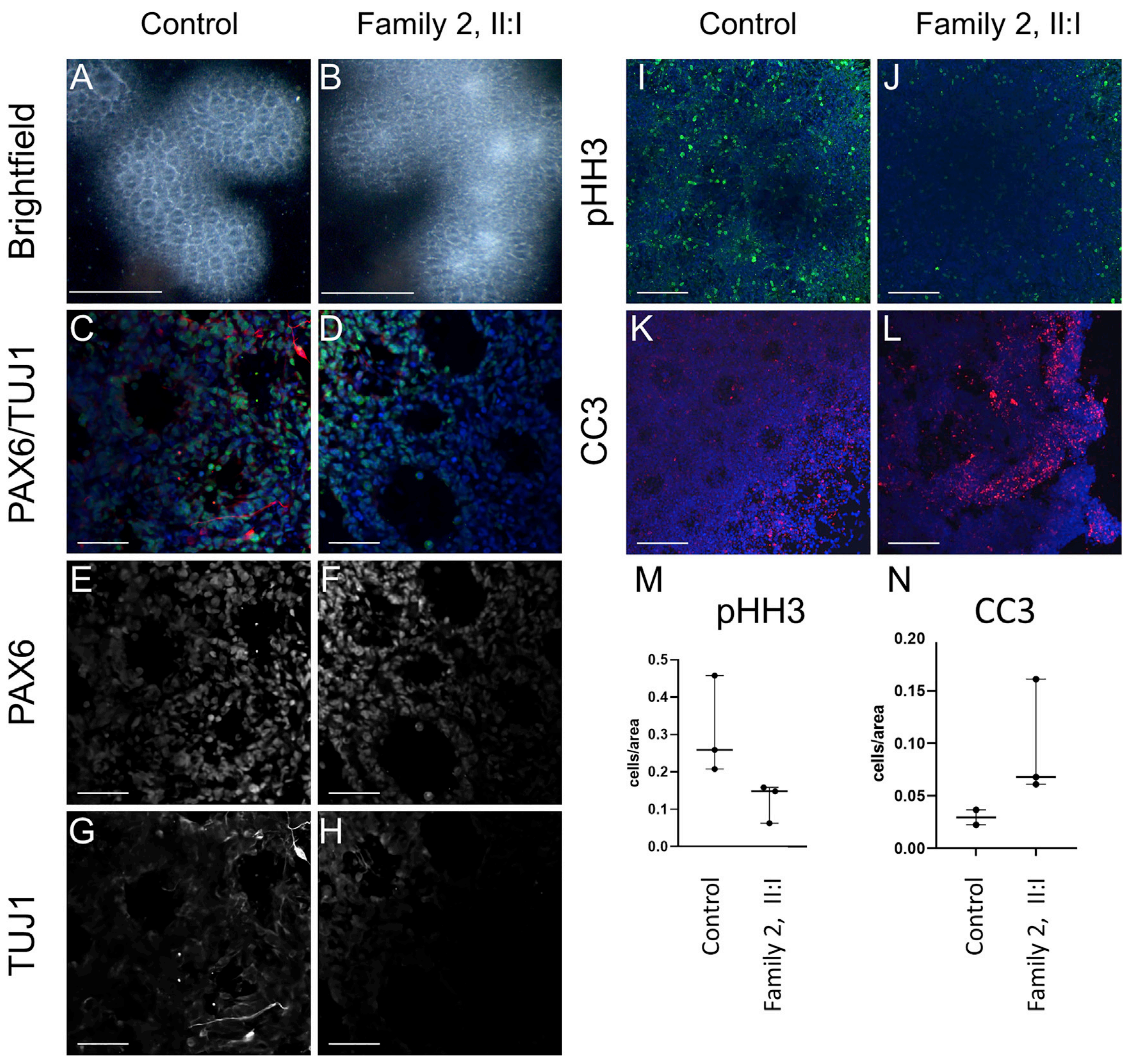

Figure 3. iPSCs from affected individuals display decreased proliferation and differentiation and increased apoptosis of neural progenitor cells

(A and B) Brightfield images showing abnormal clustering of cells in rosettes from an affected individual at 11 days *in vitro*. Control cell rosettes have a clearly visible interior region of reduced cell density as compared to more dense cells from the affected individual. Scale bar, 500 μm.

(C–L) Immunohistochemistry (IHC) analysis highlights molecular features of affected-individual-derived rosettes. Scale bars, 50 μm.

(C–H) IHC for PAX6 (green, a marker of neuronal progenitors), and TUJ1/TUBB3 (red, marks differentiated neurons) demonstrated significantly fewer differentiated neurons at 11 days in rosettes derived from affected individual iPSCs compared to control iPSCs. (E–H) PAX6 (E and F) and TUJ1 (G and H) shown independently.

(I and J) IHC for phosphohistone H3 (pHH3) shows reduced staining suggesting a proliferation defect in rosettes derived from iPSCs obtained from an affected individual. (K and L) IHC for cleaved caspase-3 (CC3) demonstrating increased apoptosis in rosettes derived from iPSCs obtained from an affected individual.

(M and N) Quantification of counts for PHH3+ cells ($n = 3$ images × 3 replicates) and CC3+ cells ($n = 3$ images × 3 replicates). iPSCs from the affected individual were derived from family 2, II:1, one clone of which was received from the Genome Engineering and Stem Cell Center, Department of Genetics, School of Medicine, Washington University in Saint Louis. Control cells are from iPSC line iPSC72.3.

Together our clinical, genetic, murine, and molecular findings define bi-allelic likely loss-of-function variants in *CAMSAP1* as a cause of a recognizable syndromic microcephalic neuronal migration disorder. The cardinal clinical features of the *CAMSAP1*-related neuronal migration disorder in humans include craniofacial dysmorphism comprising of large ears, a prominent metopic suture, wide nasal bridge and pronounced cupids bow (7/7 individuals, Figure S1), primary microcephaly (7/7 individuals, defined as >2 standard deviations below the mean for age and sex; i.e., Z score $= -2$),[25] severe to profound global developmental delay (7/7 individuals), feeding difficulties (7/7), sometimes requiring gastrostomy (2/7), cortical visual impairment (6/6 for which data was available), and seizures (6/7), typically infantile spasms with onset before one year of age, progressing to other generalized seizures refractory to antiepileptic treatment. Neurological findings although variable, include central hypotonia, with

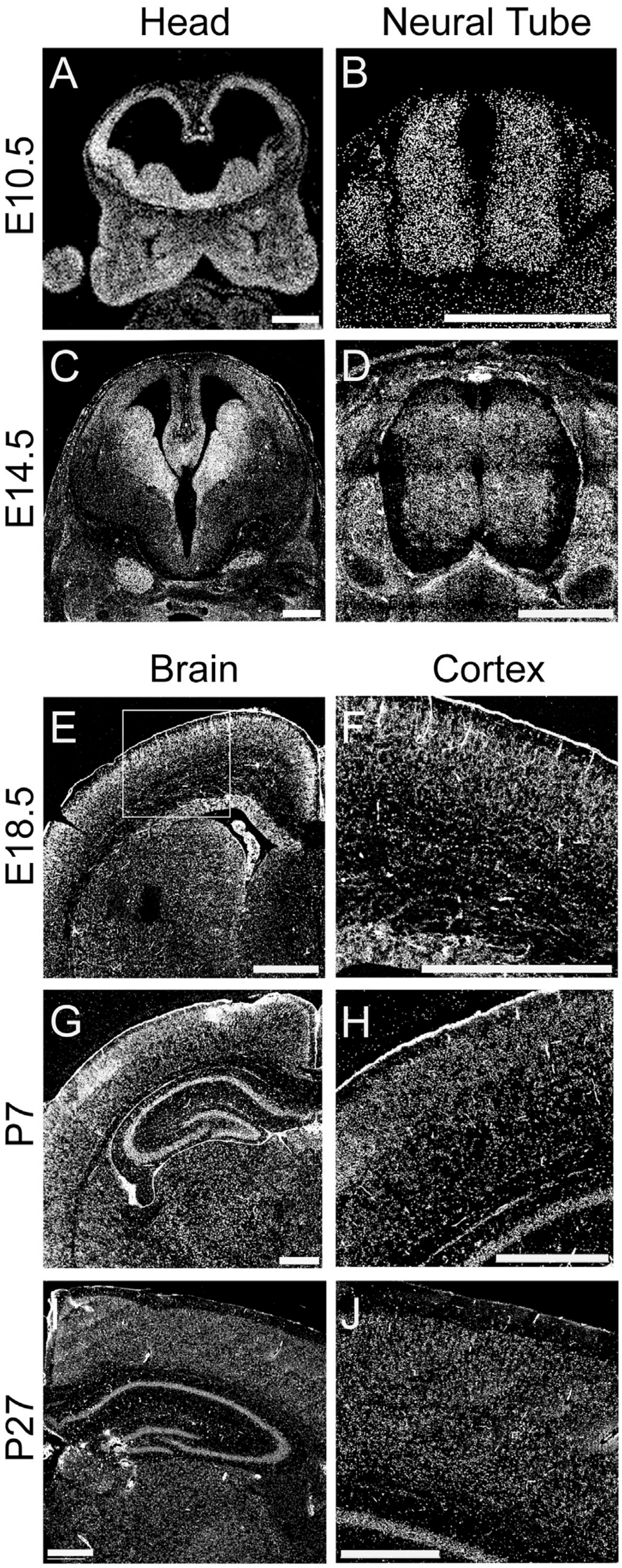

Figure 4. Expression of *Camsap1* in the CNS and developing facial primordia
RNAScope probe for *Camsap1* demonstrate robust mRNA expression at E10.5 (A and B) and E14.5 (C and D) in the developing head (A and C), and neural tube (B and D) with a clear enrichment in the neural tissues (A–J) in addition to the developing pharyngeal arches (A). Expression at E18.5 (E and F), P7 (G and H), and P27 (I and J) remains high in the brain with slightly enriched expression in upper layers (F, H, and J are higher magnification views of E, G, and I, respectively). All scale bars, 500 μm.

peripheral hypertonia, brisk reflexes, and positive Babinski sign.

The neuroradiological abnormalities (Figure 2) are strikingly consistent across all of the affected individuals in this study. Affected individuals display a classic (thick) lissencephaly with P > A gradient. In posterior regions, areas of agyria or severe pachygyria with prominent cell-sparse zones and reduced thickness of the cerebral mantle/wall are seen, with areas of less severe pachygyria and thicker cerebral mantle/wall in anterior regions. Extra-cortical features include dysplasia of the hippocampus (short, globular, and under-rotated), basal ganglia, and thalami, alongside absence of internal capsule, an absent or extremely short and thin corpus callosum, mild-moderate brainstem hypoplasia, small base of the pons, severe underdeveloped frontal horn (likely due to the basal ganglia abnormalities), enlarged third ventricle, mild borderline enlarged tectum, and cerebellar hypoplasia, although the cerebellar folia pattern remains normal (within the limits of resolution). An enlarged posterior fossa or "mega cisterna magna" was also observed in 2/5 affected individuals (Table 1).

These observed abnormalities combine cortical findings characteristic of *PAFAH1B1*(*LIS1*)-related classic lissencephaly with wider brain findings more in keeping with the tubulinopathies. The classical thick lissencephaly appearance with P > A gradient and prominent cell-sparse zone seen in the *CAMSAP1*-related neuronal migration disorder is analogous to the findings seen in *PAFAH1B1*(*LIS1*)-related lissencephaly[3] and thus predictive of a four-layer cortical histopathology. However, the extra-cortical malformations are more in keeping with a severe tubulinopathy disorder, most similar to classical *TUBA1A*-related disease.[3,10,26,27] Notable neuroradiological differences between the *CAMSAP1*-related neuronal migration disorder and the tubulinopathies involve the cerebellar and corpus callosum phenotypes. At the milder end of the tubulinopathies spectrum, cerebellar hypoplasia with an abnormal folia pattern may be the only abnormality present, while folia patterning is apparently preserved in the *CAMSAP1*-related neuronal migration disorder. Conversely, hypogenesis of the corpus callosum is typically seen in the tubulinopathies, whereas agenesis is very rarely described but was universally seen in individuals affected by the *CAMSAP1*-related neuronal migration disorder.[27] Taken together, the *CAMSAP1*-related neuroradiology may best be described as resembling that of an atypical tubulinopathy but with a distinct and unusual pattern of abnormalities (classic lissencephaly with a P > A gradient and complete agenesis of the corpus callosum), which appear pathognomonic of the disorder.

The close alignment of neuroradiological features between the tubulinopathies, *PAFAH1B1(LIS1)*-related

lissencephaly, and the *CAMSAP1*-related neuronal migration disorder is consistent with a shared pathomolecular mechanism underlying these diseases and highlights the role of the minus end of the microtubule in neuronal migration disorders. PAFAH1B1(LIS1) facilitates the function of the minus-end-directed microtubule motor dynein, and mutations in the heavy chain of cytoplasmic dynein (DYNC1H1) are associated with a neuronal migration disorder that may also show P > A gradient of lissencephaly in addition to extra-cortical malformations.[3,28] Dynein is a molecular motor that traffics substrates and organelles to this microtubule minus end, whereas CAMSAP proteins play a stabilizing role and tubulins are the core structural component.[11,16,17]

There are, however, distinct clinical, neuroradiological, and pathophysiological features only associated with the *CAMSAP1*-related neuronal migration disorder. We thus investigated the temporospatial expression of *Camsap1* to better understand these phenotypes. Previous studies have identified high *Camsap1* expression in the cortex, subventricular zone, and hippocampus of mice and rats[19,24] and in neurons, astrocytes, and their precursor neuronal stem cells.[24,29] Our murine brain expression studies determined that *Camsap1* is widely expressed early in neurogenesis in the developing brain and neural tube, in keeping with the wide range of brain malformations observed in the disorder. In addition, the fine spatial resolution that our methods enable highlight CAMSAP1 specificity to the ganglionic eminences, ventricular zone, and outer cortical layers throughout embryonic neurogenesis, in keeping with a role in neuronal migration. Supporting this, postnatal *Camsap1* expression remains evident in the outer cortex and hippocampus, the endpoints of these migration routes. The normal development of these structures is critically dependent on precisely controlled molecular mechanisms, which govern the highly intricate neuronal migration events ongoing during these developmental stages.[30] Our findings thus implicate *Camsap1* in these neuronal migration processes, potentially explaining the clinical findings in affected individuals with the *CAMSAP1*-related neuronal migration disorder.

Previously studied *Camsap1* knockout mice generated by Zhou et al. identified radial migration defects of cortical neurons leading to cortical laminar disorganization and a significant increase of neurons in the intermediate zone after the completion of neuronal migration.[19] Although mice do not have sufficiently folded brains to adequately model human lissencephaly, the identification of impaired neuronal migration by Zhou et al.[19] is highly consistent with the lissencephaly phenotype identified here. The mice were born in Mendelian ratios, although they exhibited reduced brain and body size and a high level of early postnatal mortality resulting from seizures in the immediate postnatal period.[19] This phenotype is analogous to the early and severe forms of epilepsy invariably observed in individuals with the *CAMSAP1*-related neuronal migration disorder (Table 1), with the lack of perinatal mortality observed in humans potentially explained by the efficacy of pharmacological anti-epileptic therapies. Our studies of *Camsap1*$^{null/null}$ mice indicate a significantly increased mortality occurring in the third trimester or at birth for null animals, with survival into the second trimester (E14.5–E18.5) unaffected. This may reflect a variation in phenotype severity, leading to prenatal seizures or CNS abnormalities affecting fundamental physiological processes and related to differences in the genomic knockout strategies and/or potentially different genetic strains used in generating each murine model. Alternatively, stochastic variation of expression of a dosage-sensitive gene may play a role. While we did not observe gross anatomical or histological changes in the second trimester (E14.5–E18.5), postnatal histological findings in the mice generated by Zhou et al. suggest that the absence of CAMSAP1 leads to disorganization of cortical layering.[19]

Histological studies in mammalian neurons have established that CAMSAP1 inhibits neurite extension[12] and is critical for the differentiation of neurites into mature axons and dendrites.[19] A proposed mechanism for ensuring the development of (typically) a single neuronal axon involves the accumulation of CAMSAP1-associated microtubule clusters in the longest neurite, given the finding that depletion of CAMSAP1 results in an abnormal multi-axon phenotype.[19] The kinase MARK2, which phosphorylates CAMSAP1, controlling its ability to bind microtubules, appears to regulate this process.[19] Additionally, a role in neuronal polarization and axonal/dendritic differentiation may not be unique to CAMSAP1: other studies also implicate a similar role for CAMSAP2 and CAMSAP3[31] in this process.[17,18] Other previous *in vitro* and animal studies have suggested roles for CAMSAP1 in mammalian neuronal polarization, axon/dendrite differentiation, and cytotaxis comprising important elements of neuronal migration, findings that are now consolidated by our studies.

The discovery of a non-tubulin cause of a tubulinopathy-like disorder highlights other microtubule-associated proteins, including microtubule minus-end-targeting proteins, as candidate genetic causes of neuronal migration disorders.

Data and code availability

Full exome sequencing data are not available because of reasons of confidentiality; anonymized variant data will be made available on reasonable request. The authors declare that all other data are contained within the manuscript and supplemental materials. The accession numbers for the *CAMSAP1* variants reported in this paper are ClinVar: SCV002574746–SCV002574751.

Supplemental information

Supplemental information can be found online at https://doi.org/10.1016/j.ajhg.2022.09.012.

Acknowledgments

First and foremost, we are grateful to the families for taking part in this study and Landon's League Foundation (https://landonsleague.com/) for their ongoing support of our work. The study was supported by the following grants: NIDCR R01 DE027091 (R.W.S.); MRC G1002279 (A.H.C.), G1001931 (E.L.B.), Proximity to Discover and Confidence in Concept grants MC-PC-18047, MC_PC_15054, MC_PC_15047 (University of Exeter, E.L.B. and A.H.C.); Medical Research Foundation MRF-145-0006-DG-BAPL-C0788 (E.L.B. and A.H.C.). The iPSC72.3, CCHMC PSCF iPSC line used as a control in this study was generated by the Cincinnati Children's Pluripotent Stem Cell Facility. This work was supported, in part, by US National Human Genome Research Institute (NHGRI)/National Heart Lung and Blood Institute (NHLBI) UM1 HG006542 to the Baylor Hopkins Center for Mendelian Genomics (BHCMG, J.R.L.). This research was funded in whole, or in part, by the Wellcome Trust (GW4-CAT Fellowship 216279/Z/19/Z [J.F.]). For the purpose of open access, the author has applied a CC BY public copyright license to any Author Accepted Manuscript version arising from this submission.

Author contributions

E.L.B., A.H.C., R.W.S., and R.K.N. conceived and supervised the study. R.W.S., D.T.C., and X.C. supervised cellular work. E.L.B., J.F., W.B.D., R.K.N., J.L.G., K.C.K., A.G., L.P., C.G.S., P.D.T., R.R., T.M., D.P., Z.C.A., J.E.P., and J.R.L. collated and analyzed clinical and genomic data. R.N., J.F., J.S.L., N.U., T.M., J.E.P., and Z.C.A. performed genetic studies/analysis. K.I., L.B., and E.K. performed cell and mouse studies. E.L.B., A.H.C., and J.R.W. coordinated/managed collaborations. J.F., K.I., W.B.D., A.H.C., R.W.S., and E.L.B. wrote the initial draft of the manuscript.

Declaration of interests

The authors declare no competing interests.

Received: August 4, 2022
Accepted: September 27, 2022
Published: October 24, 2022

References

1. Oegema, R., Barakat, T.S., Wilke, M., Stouffs, K., Amrom, D., Aronica, E., Bahi-Buisson, N., Conti, V., Fry, A.E., Geis, T., et al. (2020). International consensus recommendations on the diagnostic work-up for malformations of cortical development. Nat. Rev. Neurol. *16*, 618–635.
2. Severino, M., Geraldo, A.F., Utz, N., Tortora, D., Pogledic, I., Klonowski, W., Triulzi, F., Arrigoni, F., Mankad, K., Leventer, R.J., et al. (2020). Definitions and classification of malformations of cortical development: practical guidelines. Brain *143*, 2874–2894.
3. Di Donato, N., Chiari, S., Mirzaa, G.M., Aldinger, K., Parrini, E., Olds, C., Barkovich, A.J., Guerrini, R., and Dobyns, W.B. (2017). Lissencephaly: Expanded imaging and clinical classification. Am. J. Med. Genet. *173*, 1473–1488.
4. Fry, A.E., Cushion, T.D., and Pilz, D.T. (2014). The genetics of lissencephaly. Am. J. Med. Genet. C Semin. Med. Genet. *166C*, 198–210.
5. Smith, D.S., Niethammer, M., Ayala, R., Zhou, Y., Gambello, M.J., Wynshaw-Boris, A., and Tsai, L.H. (2000). Regulation of cytoplasmic dynein behaviour and microtubule organization by mammalian Lis1. Nat. Cell Biol. *2*, 767–775.
6. Di Donato, N., Timms, A.E., Aldinger, K.A., Mirzaa, G.M., Bennett, J.T., Collins, S., Olds, C., Mei, D., Chiari, S., Carvill, G., et al. (2018). Analysis of 17 genes detects mutations in 81% of 811 patients with lissencephaly. Genet. Med. *20*, 1354–1364.
7. Caspi, M., Atlas, R., Kantor, A., Sapir, T., and Reiner, O. (2000). Interaction between LIS1 and doublecortin, two lissencephaly gene products. Hum. Mol. Genet. *9*, 2205–2213.
8. Kumar, R.A., Pilz, D.T., Babatz, T.D., Cushion, T.D., Harvey, K., Topf, M., Yates, L., Robb, S., Uyanik, G., Mancini, G.M.S., et al. (2010). TUBA1A mutations cause wide spectrum lissencephaly (smooth brain) and suggest that multiple neuronal migration pathways converge on alpha tubulins. Hum. Mol. Genet. *19*, 2817–2827.
9. Desikan, R.S., and Barkovich, A.J. (2016). Malformations of cortical development. Ann. Neurol. *80*, 797–810.
10. Romaniello, R., Arrigoni, F., Fry, A.E., Bassi, M.T., Rees, M.I., Borgatti, R., Pilz, D.T., and Cushion, T.D. (2018). Tubulin genes and malformations of cortical development. Eur. J. Med. Genet. *61*, 744–754.
11. Hendershott, M.C., and Vale, R.D. (2014). Regulation of microtubule minus-end dynamics by CAMSAPs and Patronin. Proc. Natl. Acad. Sci. USA. *111*, 5860–5865.
12. Baines, A.J., Bignone, P.A., King, M.D.A., Maggs, A.M., Bennett, P.M., Pinder, J.C., and Phillips, G.W. (2009). The CKK domain (DUF1781) binds microtubules and defines the CAMSAP/ssp4 family of animal proteins. Mol. Biol. Evol. *26*, 2005–2014.
13. Pavlova, G.A., Razuvaeva, A.V., Popova, J.V., Andreyeva, E.N., Yarinich, L.A., Lebedev, M.O., Pellacani, C., Bonaccorsi, S., Somma, M.P., Gatti, M., and Pindyurin, A.V. (2019). The role of Patronin in Drosophila mitosis. BMC Mol. Cell Biol. *20*, 24.
14. Akhmanova, A., and Hoogenraad, C.C. (2015). Microtubule minus-end-targeting proteins. Curr. Biol. *25*, R162–R171.
15. King, M.D.A., Phillips, G.W., Bignone, P.A., Hayes, N.V.L., Pinder, J.C., and Baines, A.J. (2014). A conserved sequence in calmodulin regulated spectrin-associated protein 1 links its interaction with spectrin and calmodulin to neurite outgrowth. J. Neurochem. *128*, 391–402.
16. Atherton, J., Jiang, K., Stangier, M.M., Luo, Y., Hua, S., Houben, K., van Hooff, J.J.E., Joseph, A.-P., Scarabelli, G., Grant, B.J., et al. (2017). A structural model for microtubule minus-end recognition and protection by CAMSAP proteins. Nat. Struct. Mol. Biol. *24*, 931–943.
17. Jiang, K., Hua, S., Mohan, R., Grigoriev, I., Yau, K.W., Liu, Q., Katrukha, E.A., Altelaar, A.F.M., Heck, A.J.R., Hoogenraad, C.C., and Akhmanova, A. (2014). Microtubule minus-end stabilization by polymerization-driven CAMSAP deposition. Dev. Cell *28*, 295–309.
18. Yau, K.W., van Beuningen, S.F.B., Cunha-Ferreira, I., Cloin, B.M.C., van Battum, E.Y., Will, L., Schätzle, P., Tas, R.P., van Krugten, J., Katrukha, E.A., et al. (2014). Microtubule minus-end binding protein CAMSAP2 controls axon specification and dendrite development. Neuron *82*, 1058–1073.
19. Zhou, Z., Xu, H., Li, Y., Yang, M., Zhang, R., Shiraishi, A., Kiyonari, H., Liang, X., Huang, X., Wang, Y., et al. (2020). CAMSAP1 breaks the homeostatic microtubule network to instruct neuronal polarity. Proc. Natl. Acad. Sci. USA. *117*, 22193–22203.

20. Laver, T.W., De Franco, E., Johnson, M.B., Patel, K.A., Ellard, S., Weedon, M.N., Flanagan, S.E., and Wakeling, M.N. (2022). SavvyCNV: Genome-wide CNV calling from off-target reads. PLoS Comput. Biol. *18*, e1009940.
21. Sobreira, N., Schiettecatte, F., Valle, D., and Hamosh, A. (2015). GeneMatcher: a matching tool for connecting investigators with an interest in the same gene. Hum. Mutat. *36*, 928–930.
22. Mitani, T., Isikay, S., Gezdirici, A., Gulec, E.Y., Punetha, J., Fatih, J.M., Herman, I., Akay, G., Du, H., Calame, D.G., et al. (2021). High prevalence of multilocus pathogenic variation in neurodevelopmental disorders in the Turkish population. Am. J. Hum. Genet. *108*, 1981–2005.
23. Liegel, R.P., Finnerty, E., Blizzard, L., DiStasio, A., Hufnagel, R.B., Saal, H.M., Sund, K.L., Prows, C.A., and Stottmann, R.W. (2019). Using human sequencing to guide craniofacial research. Genesis *57*, e23259.
24. Yamamoto, M., Yoshimura, K., Kitada, M., Nakahara, J., Seiwa, C., Ueki, T., Shimoda, Y., Ishige, A., Watanabe, K., and Asou, H. (2009). A new monoclonal antibody, A3B10, specific for astrocyte-lineage cells recognizes calmodulin-regulated spectrin-associated protein 1 (Camsap1). J. Neurosci. Res. *87*, 503–513.
25. Centers for Disease Control and Prevention (2020). Facts about Microcephaly. https://www.cdc.gov/ncbddd/birthdefects/microcephaly.html.
26. Bahi-Buisson, N., Poirier, K., Fourniol, F., Saillour, Y., Valence, S., Lebrun, N., Hully, M., Bianco, C.F., Boddaert, N., Elie, C., et al. (2014). The wide spectrum of tubulinopathies: what are the key features for the diagnosis? Brain *137*, 1676–1700.
27. Hebebrand, M., Hüffmeier, U., Trollmann, R., Hehr, U., Uebe, S., Ekici, A.B., Kraus, C., Krumbiegel, M., Reis, A., Thiel, C.T., and Popp, B. (2019). The mutational and phenotypic spectrum of TUBA1A-associated tubulinopathy. Orphanet J. Rare Dis. *14*, 38.
28. Splinter, D., Razafsky, D.S., Schlager, M.A., Serra-Marques, A., Grigoriev, I., Demmers, J., Keijzer, N., Jiang, K., Poser, I., Hyman, A.A., et al. (2012). BICD2, dynactin, and LIS1 cooperate in regulating dynein recruitment to cellular structures. Mol. Biol. Cell *23*, 4226–4241.
29. Yoshioka, N., Asou, H., Hisanaga, S.I., and Kawano, H. (2012). The astrocytic lineage marker calmodulin-regulated spectrin-associated protein 1 (Camsap1): phenotypic heterogeneity of newly born Camsap1-expressing cells in injured mouse brain. J. Comp. Neurol. *520*, 1301–1317.
30. Ayala, R., Shu, T., and Tsai, L.-H. (2007). Trekking across the Brain: The Journey of Neuronal Migration. Cell *128*, 29–43.
31. Toya, M., Kobayashi, S., Kawasaki, M., Shioi, G., Kaneko, M., Ishiuchi, T., Misaki, K., Meng, W., and Takeichi, M. (2016). CAMSAP3 orients the apical-to-basal polarity of microtubule arrays in epithelial cells. Proc. Natl. Acad. Sci. USA. *113*, 332–337.

The American Journal of Human Genetics, Volume *109*

Supplemental information

Bi-allelic *CAMSAP1* variants cause a clinically recognizable neuronal migration disorder

Reham Khalaf-Nazzal, James Fasham, Katherine A. Inskeep, Lauren E. Blizzard, Joseph S. Leslie, Matthew N. Wakeling, Nishanka Ubeyratna, Tadahiro Mitani, Jennifer L. Griffith, Wisam Baker, Fida' Al-Hijawi, Karen C. Keough, Alper Gezdirici, Loren Pena, Christine G. Spaeth, Peter D. Turnpenny, Joseph R. Walsh, Randall Ray, Amber Neilson, Evguenia Kouranova, Xiaoxia Cui, David T. Curiel, Davut Pehlivan, Zeynep Coban Akdemir, Jennifer E. Posey, James R. Lupski, William B. Dobyns, Rolf W. Stottmann, Andrew H. Crosby, and Emma L. Baple

Supplemental Note

Detailed clinical summaries

Family 1, IV:10 is a three-year-old girl born to first-cousin parents at full term following an uncomplicated pregnancy. At one month of age, she was noted to be microcephalic with absent movement on her right side and increased tone. At three years and nine months of age she had made no developmental progress and made no eye contact. She startles to noise but has no verbal or non-verbal communication. She was severely microcephalic (-6.4 SDS) with large ears, gingival hyperplasia, high palate, metopic ridging, a flat wide nasal bridge (**Fig. S1A-B**) and bilateral fifth finger clinodactyly. Neurological examination revealed generally increased peripheral tone with upgoing planter reflexes; her right arm adopts a rigid extensor posture.

Her younger male sibling (**IV:11**) was found to have microcephaly with agenesis of the corpus callosum (aCC) on an antenatal ultrasound scan at 22 weeks gestation. He developed intractable epilepsy at nine weeks and was noted to have similar craniofacial dysmorphism to his sister.

A male second cousin (**V:1**) now aged three years, was born at 36 weeks gestation weighing 2kg (-1.8 SDS), microcephaly and shortened long bones were identified on antenatal scans. He had his first generalized seizure at six weeks of age, at three years these continue to be refractory to anti-epileptic treatment. Like his cousins, he has profound global developmental delay (GDD). He displayed extreme irritability and has suffered repeated, complicated respiratory tract infections. He is severely microcephalic (-4.8 SDS) with a prominent synophrys, metopic ridging, a flat wide nasal bridge, large ears, gingival hyperplasia, high palate (**Fig. S1C-D**) and fifth finger clinodactyly bilaterally. Neurological findings include generalized hypertonia, hyperreflexia and positive Babinski sign bilaterally.

MRI neuroimaging findings in all three children (**IV:10, IV:11** and **V:1**) are consistent and include agyria/severe pachygyria with a posterior-greater-than-anterior gradient, dysmorphic basal ganglia and absent corpus callosum (V-1: **Fig. 2A-D,** IV-11: **S2A-D**).

Family 2, II:1 is a 5-year-old male, the eldest of two siblings born to unaffected, unrelated North American parents of North European ancestry (**Fig. 1**). Antenatal brain imaging showed lissencephaly, absent corpus callosum and a small cerebellum. His birth at 38+2 weeks was uncomplicated, although he was subsequently noted to be hypotonic and exhibited early feeding difficulties.

Probable tonic seizure activity began at four-to-five months old as ocular roving/deviation, arm extension, and truncal extension in addition to infantile spasms. His electroencephalogram (EEG) showed diffuse beta frequencies, poor organization and right parietal interictal discharges. Seizure activity was refractory to initial levetiracetam monotherapy which was discontinued. Repeat EEG was performed that demonstrated a “modified hypsarrhythmia pattern” classically associated with West Syndrome. A 30-day prednisolone taper stabilized this pattern; seizure activity resolved and was maintained with vigabatrin and clobazam dual-therapy for approximately 18 months. Thereafter there was good control on clobazam monotherapy

(0.5-0.6 mg/kg/day). Recently breakthrough seizure activity has occurred requiring supplementation of the treatment regimen with Topiramate (~2 mg/Kg BD). At 3 years 7 months of age growth parameters were: Height 99.1 cm (-0.2 SDS), 13.5 kg (-1.4 SDS), 45 cm (-4.8 SDS).

Aged 5 years his neurodevelopment is severely, globally, delayed despite extensive physical therapy and without regression. He can roll and support his head and trunk for short periods, although cannot sit unsupported or crawl. He tolerates 30-45 minutes of upright weight-bearing daily using a standing aid and demonstrates slow gait/locomotion while supported in a gait trainer. His axial tone is hypotonic with variable spasticity of his limbs with intermittent guarded rigidity. He has been treated with botulinum toxin IM injections, which caused unacceptable side effects, and is currently trialing Carbidopa/Levodopa (Sinemet). He has a 15° thoracic rotoscoliosis deformity of his spine and severe left hip dysplasia, with corrective surgical intervention is planned for the latter at around five and a half years of age. He can sometimes reach for toys, recognizes a few words ("book") and uses a gaze tracking device to make choices between two or three options on a screen, but his productive speech is limited to babbles and consonant sounds without purposeful phonation of words.

There have been longstanding feeding difficulties and per oral intake consists of thickened liquids from a "sippy cup" and pureed diet via flat-plastic spoon. Steady state and flash visual evoked potential (SSVEP and FVEP) demonstrate mild cortical vision impairment and ophthalmoscopy demonstrates normal appearance of optic discs bilaterally. He also has hyperopia with astigmatism for which he wears corrective lens. There have been no concerns regarding his hearing. He was born with left unilateral cryptorchidism and has been affected by chronic constipation. Examination findings demonstrated central hypotonia with peripheral spasticity and brisk reflexes without ankle clonus.

MRI (**Fig. 2E-H)** performed on day two revealed diffuse severe pachygyria with a "posterior more severe than anterior" (P>A) gradient, dysmorphic basal ganglia, absent corpus callosum and enlarged posterior fossa or "mega cisterna magna" (**Fig. 2E-H**)

Diagnostic trio exome was performed at GeneDx (U.S.A) using a proprietary targeting system and a custom developed analysis tool (Xome analyzer), with raw data later reanalyzed in Exeter for robustness using the same pipeline as for Family 1. This identified no plausible variants in known disease genes but did reveal novel, *in trans* compound heterozygous *CAMSAP1* variants in exon 11/17: a paternally inherited Chr9(GRCh38):g.135822954dupA NM_015447.4:c.1707dupT; p.(Thr570TyrFs) variant, and a maternal Chr9(GRCh38):g.135821531G>A NM_015447.4:c.3130 C>T; p.(Gln1044*) variant also in exon 11.

Family 3, II:1 is the only child of North American parents of Northern European origin, born at 41 weeks gestation with a normal birthweight (3.27kg). Abnormal movements were noticed in early infancy and diagnosed as infantile spasms at 5 months of age. Spasms continues and additional seizure semiologies were noted over time including focal episodes of arm posturing and head turning with chaotic eye movements, asymmetric tonic seizures, asymmetric epileptic spasms and occasional epileptic status. Seizures eventually responded to a combination of vigabatrin, clobazam, levetiracetam and zonisamide, following unsuccessful trials of steroids and topiramate. Cannabidiol was also trialed with unclear benefit. Ketogenic diet was not

attempted due to chronic intolerance of other formulas. EEG showed hypsarrhythmia. Microcephaly was noted at an early age with severe delay to neurodevelopment subsequently apparent without evidence of neurodevelopmental regression – at age one there was very poor trunk/head control and intermittent extensor arm posturing, but no purposeful movements and no communication. At 1 year 1 month his growth parameters were: Height 66cm (-4.2 SDS), weight 7.7kg (-14.5 SDS), OFC 42cm (-5.0 SDS).

At 1 year 8 months he has started turning his head purposefully, occasionally reaching for and picking up objects with his hands and scooting in crawling position. He has been diagnosed with cortical visual impairment with poor eye tracking and deprivation nystagmus. He was affected by severe feeding difficulties and there were concerns about aspiration risk requiring nasogastric feeding with a parenteral gastrostomy (PEG) placed at 15 months of age. On examination he has decreased central and peripheral tone with symmetrical, normal deep tendon reflexes and absent Babinski sign.

MRI neuroimaging (**Fig. 2I-L**) revealed pachygyria with thicker cerebral mantle anteriorly, enlarged 3rd ventricle and dysmorphic basal ganglia and thalami with internal capsule not seen.

Proband-only exome was performed at GeneDx (U.S.A), identified compound heterozygous predicted loss-of-function variants in *CAMSAP1,* including the same frameshift variant identified in Family 1 [Chr9(GRCh38):g.135821923_135821944del NM_015447.4:c.2717_2738del; p.(Gln906Leufs*7)] and a novel nonsense variant Chr9(GRCh38):g.135822023G>A NM_015447.4:c.2638C>T; p.(Gln880*). These were proven to be *in trans* using the short-read exome sequencing data and parental segregation was not required. A previously reported pathogenic heterozygous variant in *POLG* [NM_002693.2:c.202C>T; p.(Gln68*), inheritance unknown] was also identified. This variant has been published twice previously, in both cases associated with mitochondrial DNA depletion syndrome 4A (MIM: 203700), also known as Alpers–Huttenlocher syndrome, when *in trans* with another pathogenic *POLG* variant and with transmitting parents unaffected.[1; 2] Since no second pathogenic *POLG* variant could be identified in this individual this is unlikely to be the cause of their neurodevelopmental disorder.

Family 4, II:1 is a male child of related Turkish parents. He was born at full term weighing 3.26kg (-0.3 SDS), length 49cm (-0.6 SDS) and with a head circumference of 32 cm (-2.5 SDS). He presented with severe developmental delay, central hypotonia, limb spasticity, epilepsy and relative microcephaly. Seizures were first observed at 5 months of age as infantile spasms then progressing to other semiology, occurring most frequently on waking and in the early morning and requiring Sodium Valproate, Vigabatrin, Clobazam for control. EEG showed a burst suppression pattern.

At 5 years 6 months he has no head control or other gross motor development, no fine motor development, cortical visual impairment and an unsafe swallow to both liquids and solids. He makes sounds, but these are without clear meaning and has no communicative language. Limb movements are dyskinetic with varying spasticity of his limbs and intermittent guarded rigidity. Central tone is severely reduced, with peripheral spasticity and hyperactive deep tendon reflexes. He has a prominent and wide nasal root, relatively large ears, an open mouth with high arched palate and left sided unilateral ptosis.

At 6 years 5 months growth parameters were: Height 125cm (+1.3 SDS), weight 25kg (+1.0 SDS) OFC 53cm (-0.2 SDS).

Brain MRI (**Fig. 2M-P**) revealed diffuse lissencephaly, dysmorphic basal ganglia, a thin corpus callosum and an enlarged posterior fossa or "mega cisterna magna".

Trio exome sequencing at Baylor College of Medicine, using previously described methods),[3] identified a novel homozygous candidate nonsense variant, Chr9(GRCh38):g.135822830T>A NM_015447.4:c.1831A>T; p.(Lys611*), located within a 4.2Mb region of homozygosity.

Family 5, II:1 was the adopted child of North American parents, only limited information was available regarding her biological parents. She was born at 41+1 weeks gestation following a high-risk pregnancy with a birthweight within the normal range, and head circumference of 31.8cm (-2.2 SDS). Neurodevelopment was reported as severely delayed, rolling first at 3y10m, no speech at 4y9m and dependent for all activities of daily living (Gross Motor Function Classification System (GMFCS) level V. Dystonia, presenting as neck extension and back arching, was diagnosed at 11m of age and managed with diazepam, gabapentin, and baclofen. Seizures semiologies include complex partial seizures involving the right size and unresponsive episodes with associated eye rolling, likely generalized seizures. These were associated with EEG findings of multifocal epileptiform discharges suggesting electroclinical seizures that appeared to lateralize to either hemisphere and have been treated with vigabatrin and diazepam for extended seizures. Neurological examination revealed central hypotonia with bilateral lower extremity spasticity, and investigations were consistent with cortical visual impairment. Her hearing was normal when assessed at 11m of age. At 4 years and 9 months she was no longer able to roll her growth parameters were: Height 106.5cm (-0.1 SDS), weight 17.3kg (-0.2 SDS) and OFC 42.5cm (-7.1 SDS). During her 5th year of life she developed episodes of "emesis with dark fluid", the family opted for hospice care after an extensive evaluation for her symptoms and she died at 5.5 years of age.

Brain MRI findings (**Fig. S2E-F**) included holohemispheric bilateral lissencephaly and grey matter band heterotopia with notable white matter volume loss, a prominent cisterna magna and diffusely small brainstem with decreased volume of the dorsal pons.

Proband only exome sequencing, performed at Cincinnati Children's Hospital Medical Center and analyzed using VarSeq 2.2.3 from Golden Helix identified a homozygous *CAMSAP1* variant [Chr9:g.135818055G>C NM _015447.3: c.4193C>G p.(Ser1398*).

Supplemental Figures

Figure S1: Facial features of individuals with *CAMSAP1*-related disorder

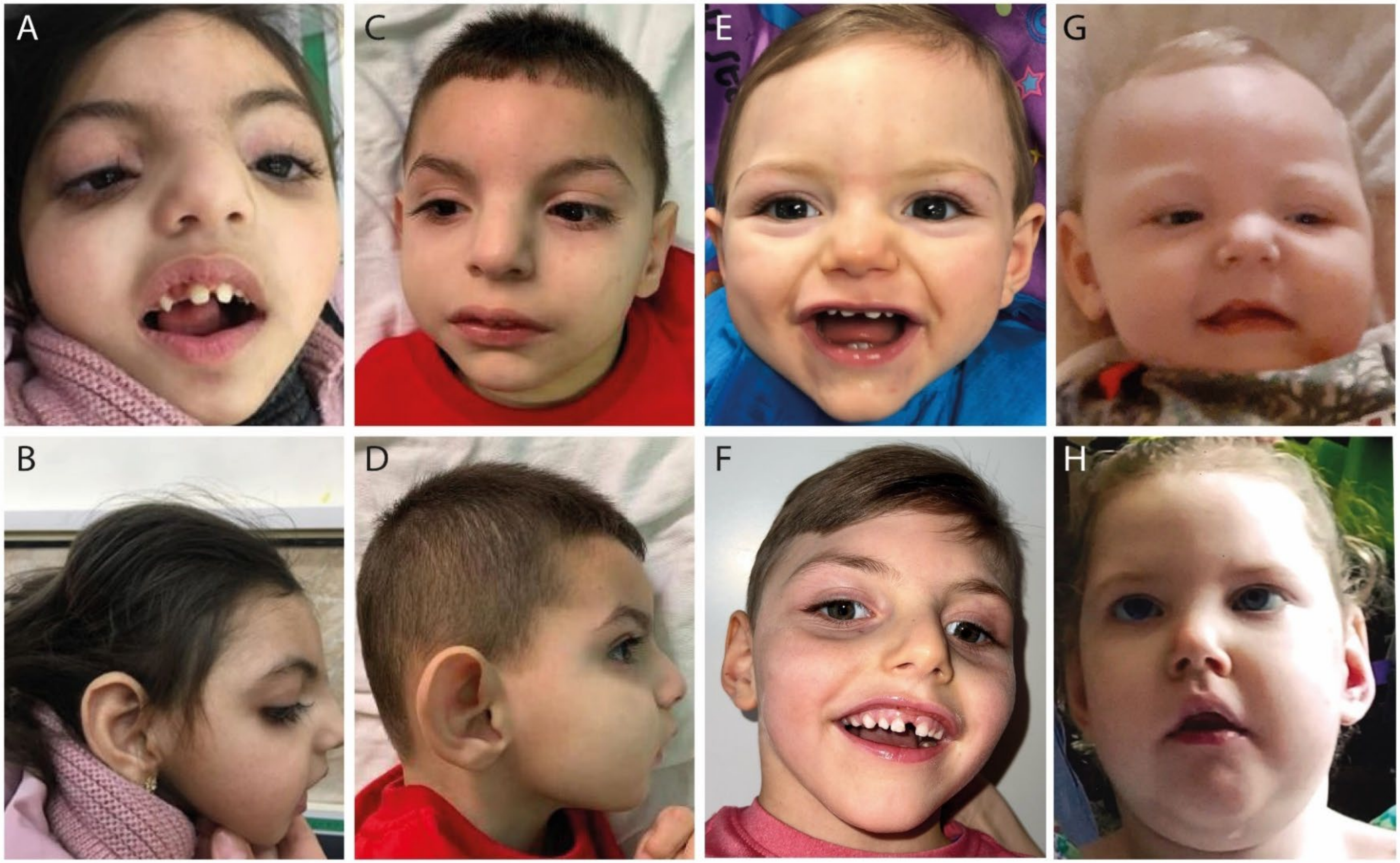

Clinical photographs demonstrating the cardinal features of the *CAMSAP1*-related disorder including microcephaly, large ears, prominent metopic suture, wide nasal bridge and pronounced cupids bow. **(A,B)** Family 1, Individual IV:10; **(C,D)** Family 1, Individual IV:11; **(E,F)** Family 2, Individual II:1; **(G)** Family 3, Individual II:1; **(H)** Family 5, Individual II:1.

Figure S2: Additional MRI brain images from individuals with *CAMSAP1*-related disorder

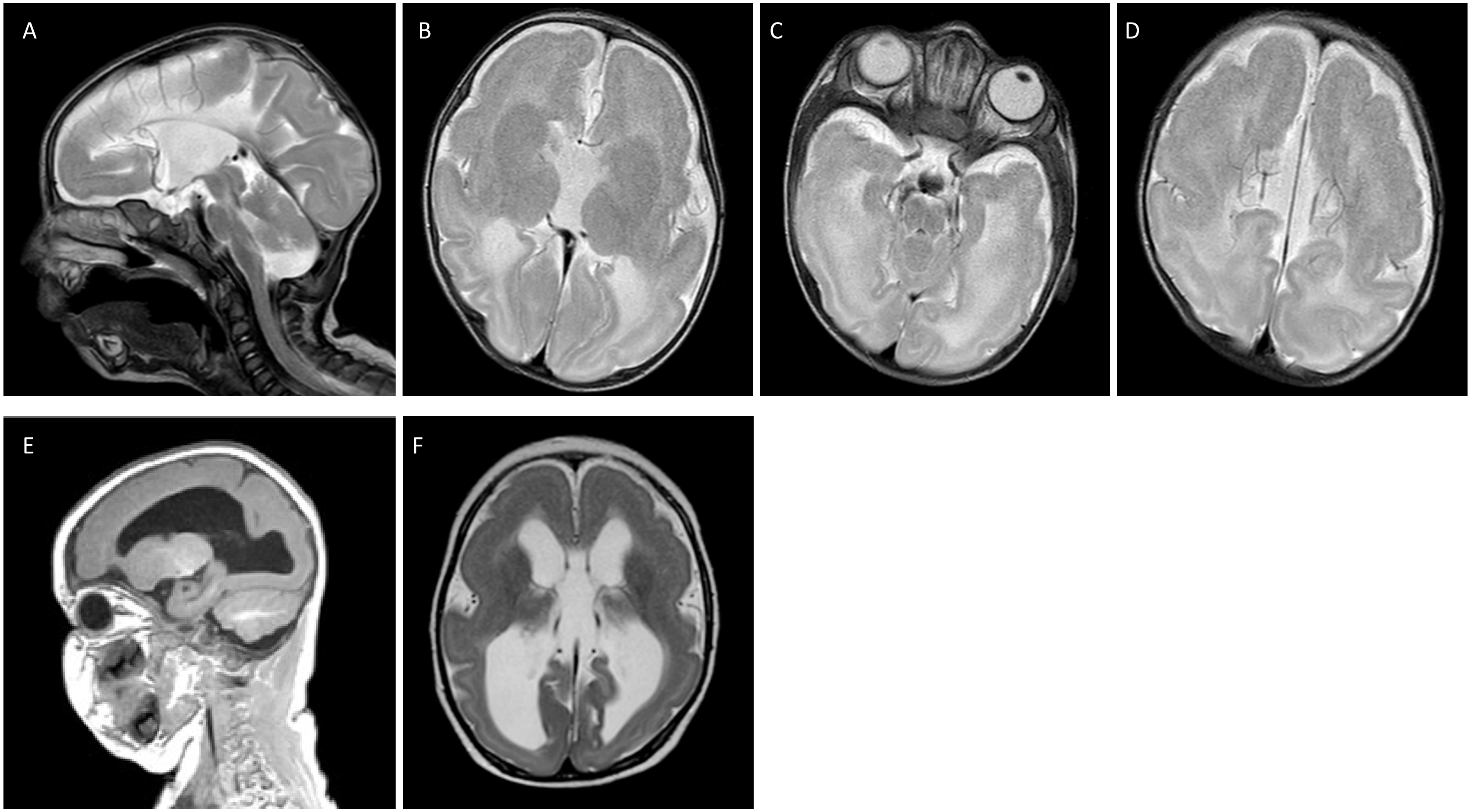

Row 1 (**A-D**) **Family 1, IV-11** aged 4 months, showing agenesis of the corpus callosum, dysmorphic basal ganglia, posterior-more-severe-than-anterior gradient pachygyria and cerebellar hypoplasia.

Row 2 (**E-F**) **Family 5, II-1.** In this case full imaging was not available for re-review, but report and static images were reviewed. There is holohemispheric bilateral lissencephaly with notable white matter volume loss and a prominent cisterna magna.

Figure S3: Homozygous regions greater than 1Mb shared between individuals IV:10 and V:1, generated using AutoMap[4]

Chr	Start	End	Mb
1	57,383,315	59,042,181	1.7
2	52,920,931	68,274,510	15.4
4	142,651,159	145,573,954	2.9
6	163,545,674	170,892,919	7.3
7	97,488,146	99,023,246	1.5
9	133,061,603	141,071,475	8.0

Chr	Start	End	Mb
12	51,585,601	52,680,008	1.1
16	58,537,897	62,851,413	4.3
20	853,502	1,961,134	1.1
21	39,764,437	41,029,831	1.3
22	27,146,768	28,293,980	1.1

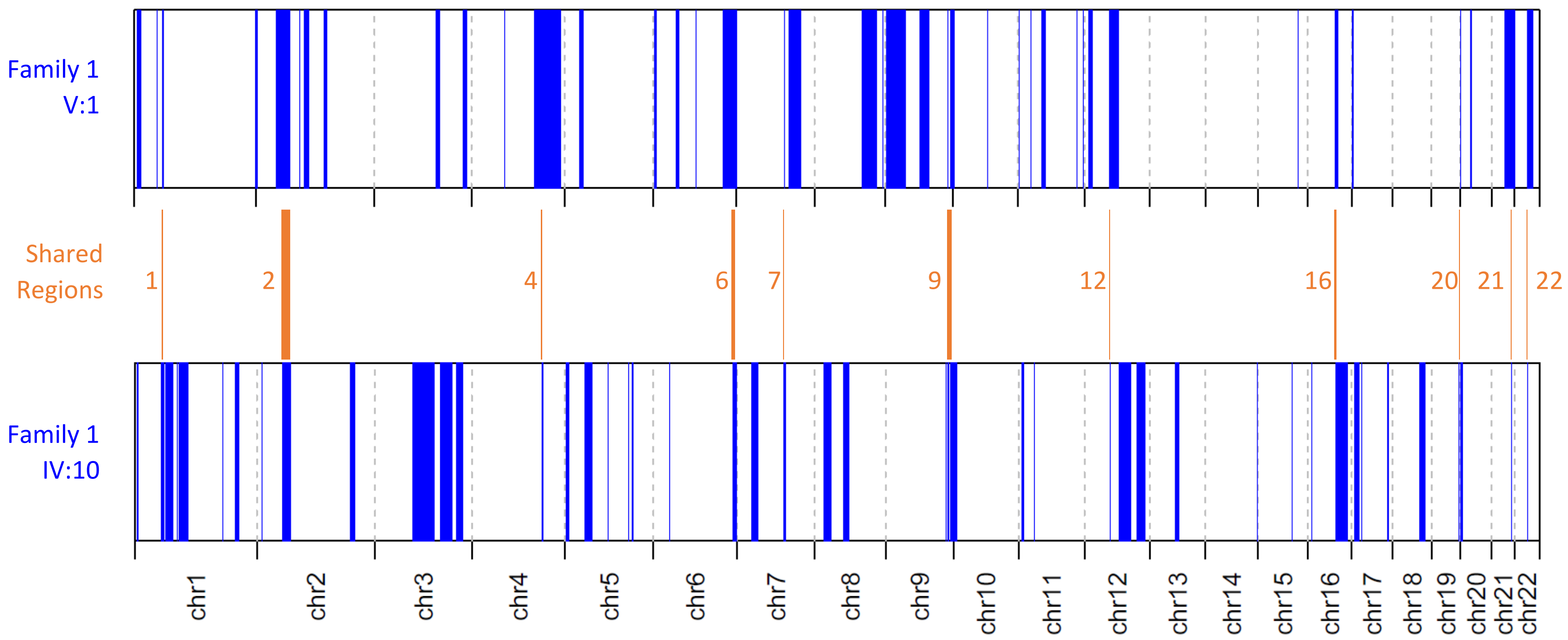

Figure S4: *CAMSAP1* sequencing chromatograms from Family 1

Individual V:1 (homozygous NM_015447.4:c.2717_2738del)

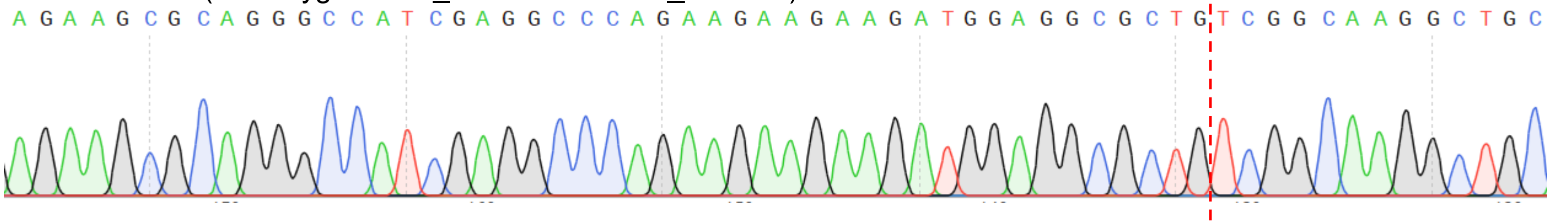

Individual IV:4 (heterozygous NM_015447.4:c.2717_2738del)

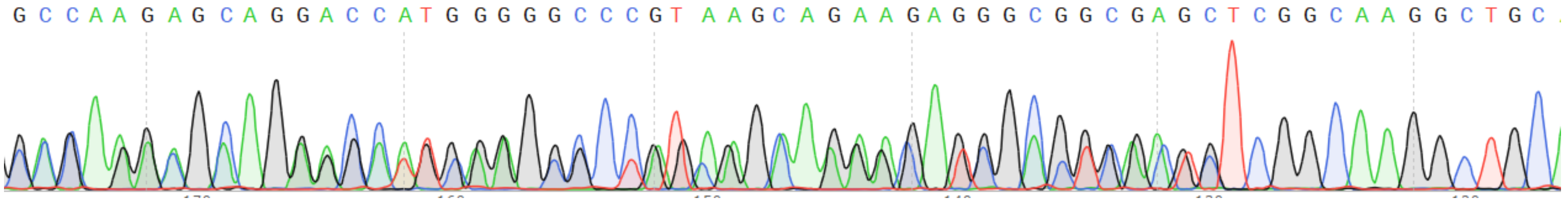

Individual IV:12 (homozygous wild type)

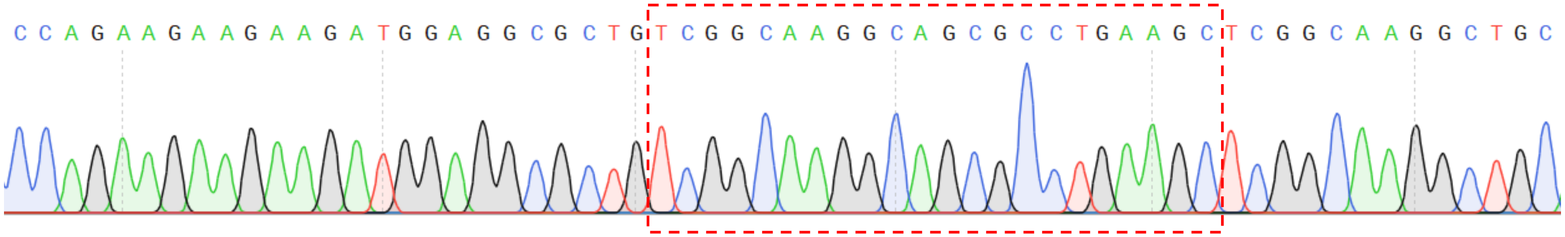

The position of the deletion is shown with a dotted red line on the chromatogram from the homozygous c.2717_2738del individual (*top*) The 22 deleted based are shown with a red box on the chromatograms from the homozygous wild-type individual (*bottom*).

Figure S5: Recurrent *CAMSAP1* deletion may result from homologous recombination

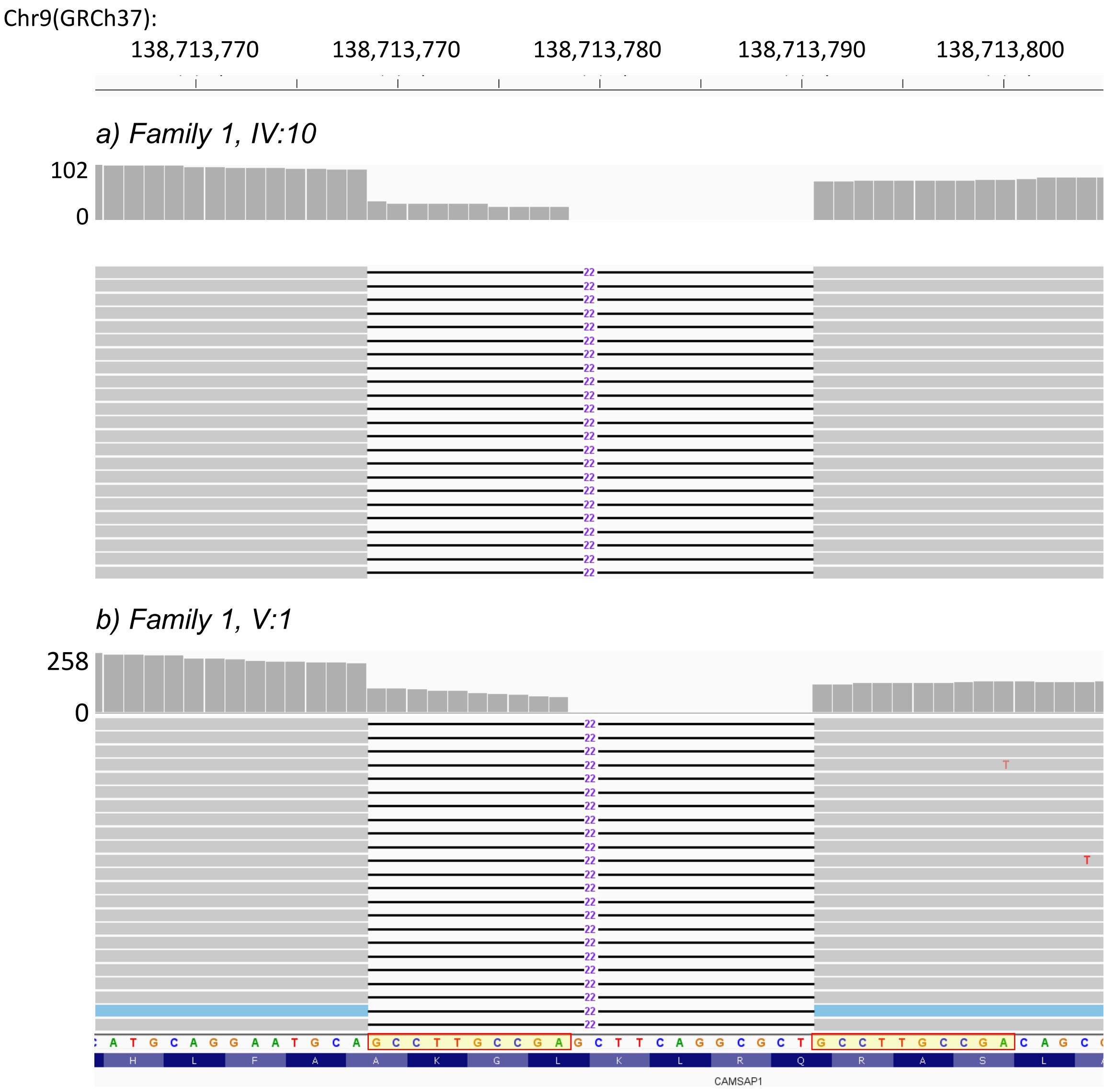

Alignment of reads generated through exome sequencing for Family 1, IV:10 and Family 1, V:1, visualized using the Integrative Genomics Viewer (version 2.9.2, Broad Institute). Position on Chromosome 9 (GRCh37) is shown at the top. Read depth is shown above a number of representative reads. The reference sequence is shown below the 22-base-pair deletion NM_015447.4:c.2717_2738del p.(Gln906Leufs*7) is shown with the homologous sequence GCCTTGCCGA highlighted in red and yellow. Recombination of these adjacent regions may be an explanation for the recurrent nature of this variant.

Figure S6: *Camsap1* genotyping and Mendelian survival

A: Conclusive genotyping example for *Camsap1* homozygous (null/null), control (wt/wt), and heterozygous (wt/null) animals. The null allele is *Camsap1*$^{em1(IMPC)J}$.

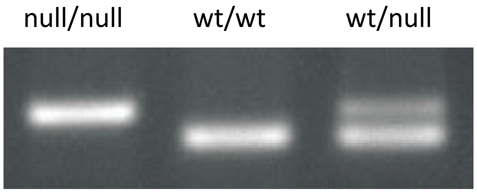

Mendelian survival tables for mice

B: **E14-E18.5:** Embryonic survival follows Mendelian ratios.

	Total	wt/wt	wt/null	null/null
Expected		12	24	12
Observed	48	9	28	11
% of total surviving	100%	19%	58%	23%

p=0.47

C: **P0-P1:** Approximately one-third of expected homozygous null animals survive birth.

	Total	wt/wt	wt/null	null/null
Expected		9	18	9
Observed	36	16	17	3
% of total surviving	100%	44%	47%	8%

p=0.087

D: P21: Homozygous null animals do not survive to weaning

	Total	wt/wt	wt/null	null/null
Expected		9.25	18.5	9.25
Observed	37	10	27	0
% of total surviving	100%	26%	69%	0%

p=0.001

Figure S7: Postnatal mutant mice do not survive in normal ratios but exhibit no skeletal abnormalities

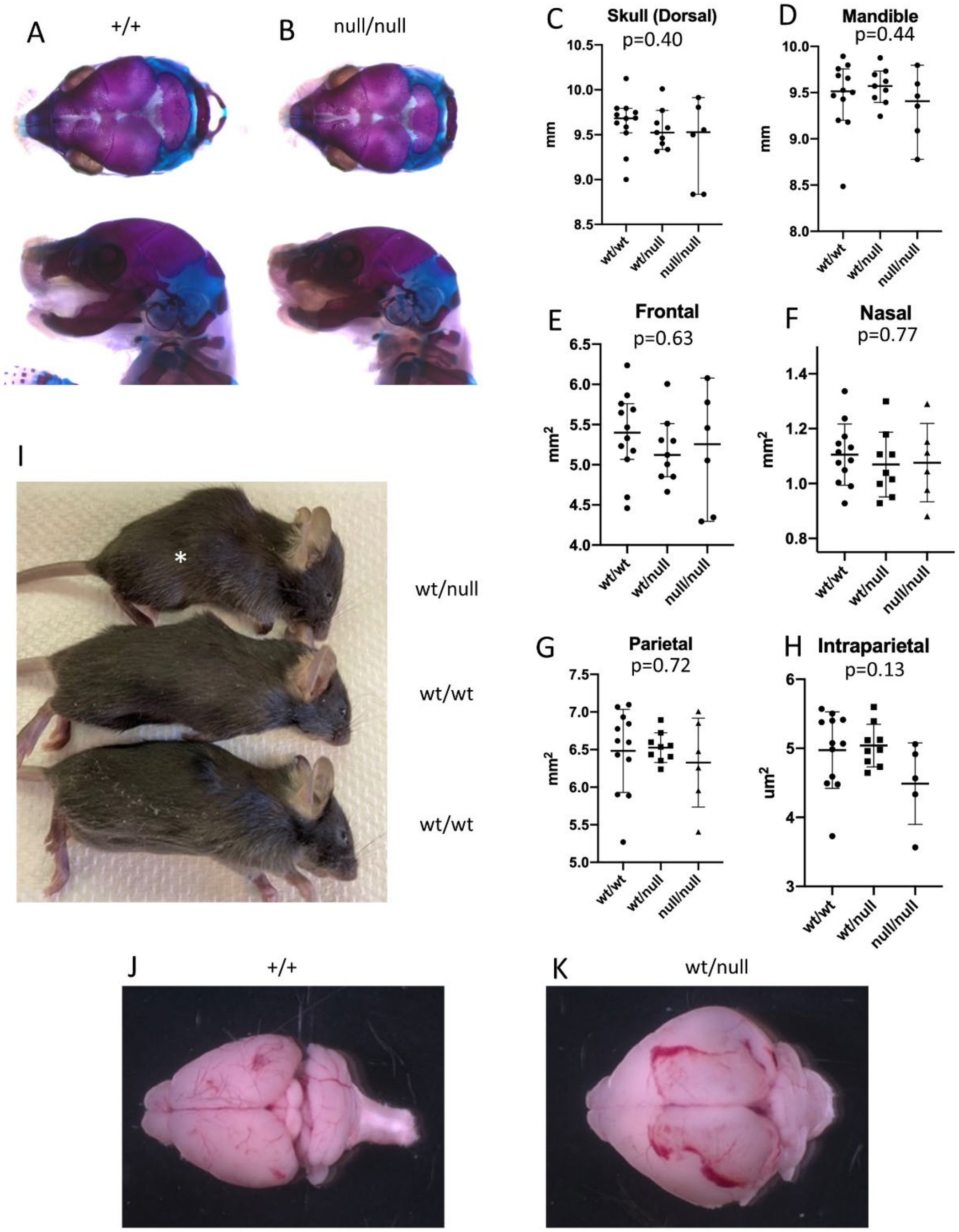

A,B - P0 skeletal preparation example images exhibiting no evident abnormalities in homozygous null animals. **C-H** - Quantification of bone length measurements from skeletal preparations (n=6-12 animals per genotype as shown in plots, Mean +/- SEM). **I** - P21 littermates, *Camsap1*$^{null/wt}$ individual with hydrocephaly marked with asterisk. **J-K** - Brains dissected from littermate control and hydrocephalic *Camsap1*$^{null/wt}$ individual. The null allele is *Camsap1*$^{em1(IMPC)J}$.

Figure S8: Embryonic *Camsap1* null mice exhibit no morphological phenotypes

Whole-mount control and *Camsap1*$^{null/null}$ embryos at stages E14.5 (**A-B**) and E16.5 (**C-D**). Hematoxylin and eosin-stained histological images of control and *Camsap1*$^{null/null}$ embryos at E14.5 (**E-F**) and E16.5 (**G-H**). No gross morphological or histological abnormalities were noted. The null allele is *Camsap1*$^{em1(IMPC)J}$.

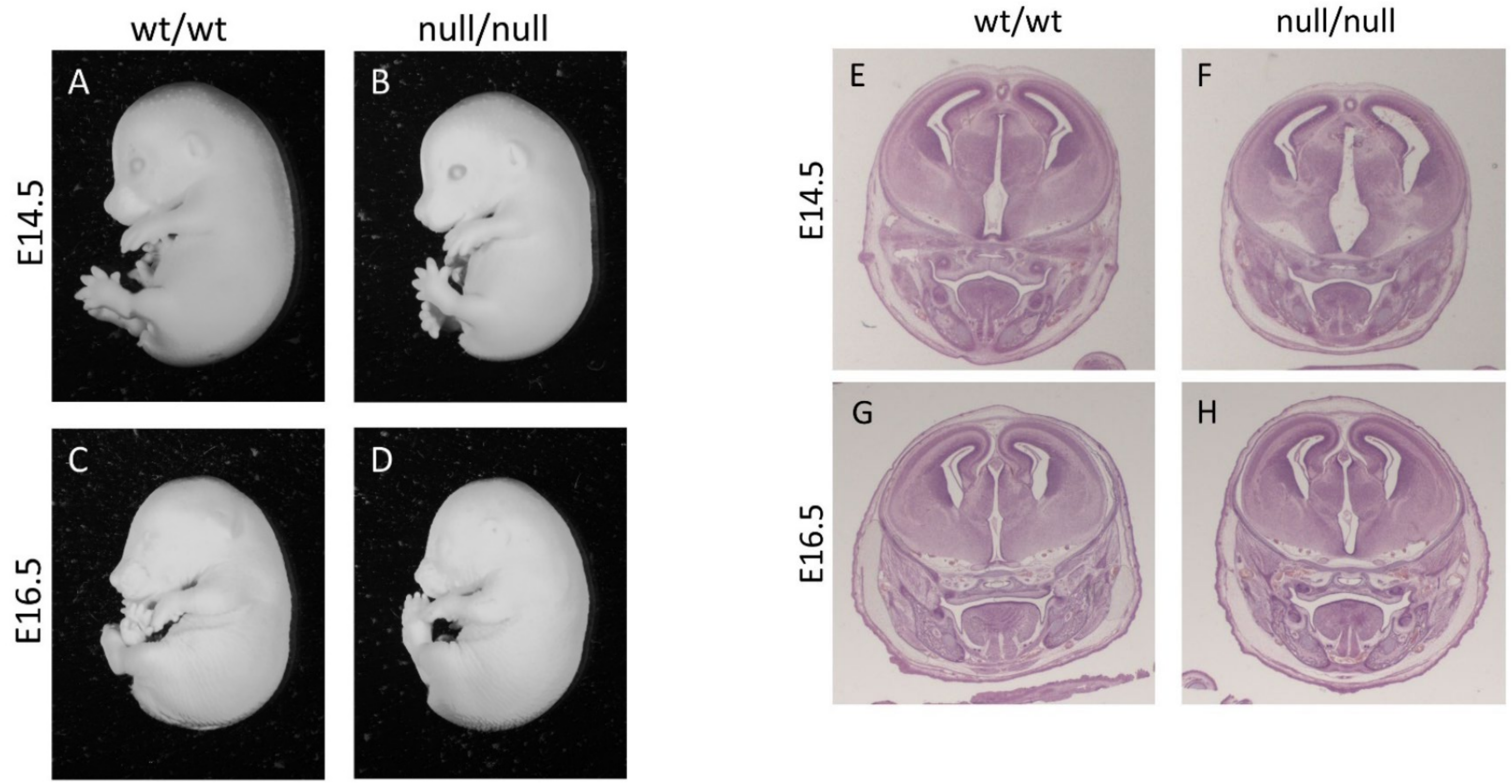

Whole-mount control and *Camsap1*$^{null/null}$ embryos at stages E14.5 (**A-B**) and E18.5 (**C-D**). Hematoxylin and eosin-stained histological images of control and *Camsap1*$^{null/null}$ embryos at E14.5 (**E-F**) and E16.5 (**G-H**). No gross morphological or histological abnormalities were noted. The null allele is *Camsap1*$^{em1(IMPC)J}$.

Figure S9: Camsap1 target region for RNA scope probe (ACDBio Cat# 866521)

Species* :	Mouse
Species (common):	House Mouse
Entrez Gene ID :	227634
Gene Alias :	9530003A05Rik
Accession No:	NM_001276359.1
Target Region :	6606 - 7920
No. of Pairs :	20

Target region as stated above:

acagt gtcccacatc ttctggcttc agggcagctg cgcatgactt ttgggttacc
6661 taggaattta tttttcatga agagttggaa gacctggtgt agagcaggaa gttgcatttc
6721 tgctcgactt ttaatacggt gacttggcat gtggcctgtc ctgaaggctg tctcagaacg
6781 cagggtctgc atgtgaagta cagtgccagt gtctcaaggc cgggctgtgg aggccatgtg
6841 gcttgatggc tcagaagcac tcgtgccttc tgtgttccat gacacagaag tagttttgat
6901 ttttttttta attaggaagt ttccggtaca ggattttgtg gtggtgatct ggagcctcca
6961 gggtgtgggt acactgtctt gaccaactgt gctgggaagg ctactcagct tttcgggcag
7021 aaagtgccaa gaaattgaat acatgacgac tcatgcagct cattccttag taacaacaga
7081 cccccccgag gagcccaggt cggttggcat taaaaatatt tctcagccgg gcggtggtgg
7141 cgcacgcctt taatccaagc acttgggagg cagagacagg cggatttctg agtttgaggc
7201 cagcctgatc tacaaagtga gttccaggac agccaggact acacagagaa accctgtctc
7261 gaaaaaataa aaaaaaaaaa tctcagaact agatgtgcag gtctacatgt ctgtgtcgat
7321 gtgggatgta cctgtgtgtg cgtatatgag tatgtgcaca caaacatgtg catatggcct
7381 gtgtacacgt gtgtatgcat gccaaagata gcgtttccag tgtgaccttt tgacctggta
7441 ttctcggtgt gttcctgcaa tacaatctcc aatcagactt ttaaggccca gactgcttca
7501 ggcaacattg aaaagtggca taatacaaaa tttacttcta gattgtttga aactatggtt
7561 gttacttgga agaagaaagt gttaaaagtc catttttctt gttgaaagtt aatcaacttg
7621 agtaaacctt tatagattgg tgctggttta cctagtgaaa tggctttgat cttggttacc
7681 tgagcctatt ggtgatttca ttctatctgt gtccagtacc acatgtgtaa agccagttct
7741 aactcctgtt ttgtgactat ggccaagtca caagccccaa ctgggaacga gccatgccca
7801 gcatcctctt gtttctcact gattccttgg caataaactt gtaccttgct gactcagggt
7861 cttcctggtg tctgaggtgc actctggagg tgcttttggt atctggtatc actggtgttc

Supplemental Tables

Table S1: Pathogenic *CAMSAP1* variants identified in this study with their frequency in population databases

CAMSAP1 variant (NM_015447.4)	Population	Chr9(GRCh38): g.	Chr9(GRCh37): g.	Nucleotide change	Exon	gnomAD v2.1.1	gnomAD v3.1.1
**c.1707dupT p.(Thr570Tyrfs*17)**	White European	135,822,954	138,714,800	insA	11/17	absent	absent
**c.1831A>T p.(Lys611*)**	Turkey	135,822,830	138,714,676	T>A	11/17	absent	absent
**c.2638C>T p.(Gln880*)**	White European	135,822,023	138,713,869	G>A	11/17	absent	absent
**c.2717_2738del p.(Gln906Leufs*7)**	Arab / White European	135,821,923_ 135,821,944	138,713,769_ 138,713,790	AGCCTTGCCGAGCTTCAGGCGCT > A	11/17	1 het	absent
**c.3130 C>T p.(Gln1044*)**	North America	135,821,531	138,713,377	G>A	11/17	absent	absent
**c.4193C>G p.(Ser1398*)**	North America	135,818,055	138,709,901	G>C	14/17	absent	absent

Abbreviations: het, heterozygous individual

Table S2: Other variants identified through exome sequencing

Individual	Variant	Zygosity, Inheritance	Gene	OMIM phenotype	Gene expression	gnomAD v2.1.1	ClinVar	SIFT	Polyphen 2	Interpretation
Family 1 V:1, IV:10 (shared)	NM_024757.4:c.623C>T; p.(Pro208Leu)	Homozygous	*EHMT1*	(AD) Kleefstra Syndrome 1; 610253	Widespread	4 het	-	0.001	0.998	Phenotype absent from parents
	NM_153710.4:c.1819G>A; p.(Asp607Asn)	Homozygous	*STKLD1*	-	Testes only	10 het	-	0.257	0.855	No phenotype, Expression only in testes
	NM_004269.3:c.107A>G; p.(Lys36Arg)	Homozygous	*MED27*	-	Esophagus, v low in brain	27 het	-	0.240	0.899	No phenotype, Low brain expression
	NM_020695.3:c.3547G>A; p.(Asp1183Asn)	Heterozygous	*REXO1*	-	Widespread	3 het	-	0.141	0.001	No phenotype, Predicted benign
	NM_020695.3:c.1493G>A; p.(Arg498His)	Heterozygous	*REXO1*	-	Widespread	13 het	-	0.022	0.955	No phenotype, Second REXO1 variant predicted benign
	NM_016252.3:c.11032A>G; p.(Ile3678Val)	Heterozygous	*BIRC6*	-	Widespread	174 het	-	0.353	0	No phenotype, predicted benign
	NM_016252.3:c.14294A>G; p.Glu4765Gly)	Heterozygous	*BIRC6*	-	Widespread	132 het	-	0.000	0.96	No phenotype, Second BIRC6 variant predicted benign
Family 2 II:1	NM_013358.2:c.592A>G; p.(Lys198Glu)	Heterozygous *de novo*	*PADI1*	-	Widespread	absent	-	0.624	0.844	No phenotype
	NM_001369.2:c.7246C>T; p.(Arg2416Cys)	Heterozygous Paternal	*DNAH5*	Ciliary dyskinesia, primary, 3, with or without situs inversus	Low in brain	2 hets	-	0.000	0.055	Phenotype absent, 2nd DNAH5 variant predicted benign
	NM_001369.2:c.4687G>A; p.(Gly1563Ser)	Heterozygous Maternal	*DNAH5*	Ciliary dyskinesia, primary, 3, with or without situs inversus	Low in brain	24 hets	Conflicting	0.54	0.002	Phenotype absent, Predicted benign
	NM_002850.3:c.1828C>G; p.(Arg610Gly)	Heterozygous Maternal	*PTPRS*	-	Widespread	1 het	-	0.36	0.081	No phenotype, Predicted benign
	NM_002850.3:c.1438G>T; p.(Val480Leu)	Heterozygous Paternal	*PTPRS*	-	Widespread	2 hets	-	0.000	0.969	No phenotype, Second PTPRS variant predicted benign
Family 3 II:1*	NM_002693.2:c.202C>T; p.(Gln68*)	Heterozygous	*POLG*	Mitochondrial DNA depletion syndrome 4A; 203700, 4B; 613622 & others	Widespread	absent	Pathogenic	-	-	Mitochondrial phenotypes are recessive, no 2nd variant
Family 4 II:1	NM_004817.4:c.2065C>T; p.(Arg689Cys)	Homozygous	*TJP2*	Cholestasis, progressive familial intrahepatic 4; 615878	Widespread	1 het	-	0.001	1	Phenotype absent in the proband
	NM_001100112.2:c.2654C>T; p.(Thr885Met)	Homozygous	*MYH2*	Proximal myopathy and ophthalmoplegia; 605637	Low in brain, high in muscle	16 het	-	0.029	0.001	Phenotype absent in the proband
	NM_014733.6:c.1670C>G; p.(Ser557Cyst)	Heterozygous *de novo*	*ZFYVE16*	-	Widespread	17 het	-	0.037	0.995	No phenotype
Family 5 II:1	NM_001099439.1:c.712G>A; p.Gly238Arg	Heterozygous	*EPHA10*	-	Low expression in cortex	3 hets for same aa	-	0.000	-	No phenotype, Low brain expression
	NM_001134479.1:c.442T>C; p.Phe148Leu	Heterozygous	*LRRC8D*	-	Widespread	absent	-	0.003	0.975	No phenotype

Individual	Variant	Zygosity, Inheritance	Gene	OMIM phenotype	Gene expression	gnomAD v2.1.1	ClinVar	SIFT	Polyphen 2	Interpretation
Family 5 II:1	NM_001531.2:c.602_603delCA; p.Thr201Argfs*47	Heterozygous	*MR1*	-	Widespread	1, multiple fs* in earlier aa	-	-	-	No phenotype
	NM_018263.4:c.1952G>C; p.Arg651Thr	Heterozygous	*ASXL2*	Shashi-Pena syndrome; 617190	Widespread	absent	-	0.000	0.942	Does not match established phenotypes
Family 5 II:1	NM_001277115.1:c.2966G>A; p.Arg989Gln	Heterozygous	*DNAH11*	Ciliary dyskinesia, primary, 7; 611884	Low expression in cortex	1 het for same amino acid	Conflicting	0.004	0.994	Does not match established phenotypes
	NM_006955.2:c.1910A>G; p.Tyr637Cys	Heterozygous	*ZNF33B*	-	Widespread	1 het	-	0.001	0.991	No phenotype
	NM_019006.3:c.579C>G; p.Ile193Met	Heterozygous	*ZFAND6*	-	Widespread	absent	-	0.002	0.991	No phenotype
	NM_018146.3:c.175delC; p.Arg59Alafs*46	Heterozygous	*MRM3*	-	Widespread	2 het for same fs*	-	-	-	No phenotype
	NM_030962.3:c.191C>T; p.Thr64Met	Homozygous	*SBF2*	Charcot-Marie-Tooth disease, type 4b2; 604563	Widespread	absent	-	0.011	0.996	Does not match established phenotypes
	NM_001039958.1: c.546_558delAGGGCAGGGGCAG; p.Gln184Argfs	Homozygous	*MESP2*	Spondylocostal dysostosis 2, autosomal recessive; 608681	Low expression in cortex	absent	-	-	-	Does not match established phenotypes
	NM_001145402.1:c.2344delG; p.Glu782Argfs*2	Homozygous	*FAM71E2*	-	Low expression in cortex	absent	-	-	-	No phenotype, Low brain expression
	NM_001145402.1:c.2333delA; p.Gln778Argfs*6	Homozygous	*FAM71E2*	-	Low expression in cortex	absent	-	-	-	No phenotype, Low brain expression
	NM_152503.5:c.93-1_93insCTTATAGACAGGGCC CCGCGGCCGGCACT; p.Asn31Lysfs*10,	Homozygous	*MROH8, RPN2*	-	Widespread	absent	-	-	-	No phenotype
	NM_032796.3:c.455G>A; p.Arg152His	Homozygous	*SYAP1*	-	Widespread	1 het	-	0.000	0.994	No phenotype

Abbreviations: aa = amino acid, fs = frameshift variant, het = heterozygous individual, hom = homozygous individual, *A full variant list was not available for Family 3, II:1. A low SIFT score suggests pathogenicity as does a high Polyphen2 score.

Table S3: Antibodies used in immunocytochemistry experiments.

Antibody Name	Concentration	Source
Cleaved Caspase-3	1:1000	Cell Signaling
Pax6	1:500	MBL
PHH3	1:500	Sigma
TUJ1	1:500	Abcam

Supplemental Methods

Clinical and genetic methods

DNA was extracted from blood/buccal samples using standard techniques. Exome sequencing was performed using DNA from individuals V:1 and IV:10 (Family 1) using either Agilent SureSelect Whole Exome v6 (Agilent Technologies, Santa Clara, CA) or Twist Human Core Exome Kit (Twist Bioscience, San Francisco, CA) exon targeting respectively. Reads were aligned (BWA-MEM v0.7.17), mate-pairs fixed and duplicates removed (Picard v2.15.0), InDel realignment/base quality recalibration (GATK v3.7.0), single-nucleotide variant (SNV) / InDel detection (GATK HaplotypeCaller), annotation (Alamut v1.8), and read depth was determined for the whole exome through our in-house pipeline. This conforms to GATK best practices. Variants were filtered based on call quality, segregation with disease, impact on gene function and allele frequency in population databases. Homozygous or compound heterozygous variants present in exons or adjacent intronic regions were evaluated and assessed for clinical correlation with phenotype.

Diagnostic exome was performed for Individual II:1 (Family 2; trio of both parents and proband) and Individual II:1 (Family 3; proband only) at GeneDx (U.S.A) using a proprietary targeting system and a custom developed analysis tool (Xome analyzer). Raw data from Individual II:1 (Family 2) was also re-analyzed using the same bioinformatic pipeline and filtering strategy as in Family 1, with the inclusion of the analysis of *de novo* variants. Trio exome was performed for Individual II:1 (Family 4) at Baylor College of Medicine as described.[5] Individual II:1 (Family 5) was analyzed using - VarSeq 2.2.3 from Golden Helix. Protein coding variants with plausible variant allele fraction (VAF - 0.3-0.7 for heterozygous variants) were selected and filtered by frequency (MAF < 0.0001, <2 individuals in gnomAD for heterozygous variants) and CADD score prediction (>25).

iPSC culture

Human induced pluripotent stem cells (iPSCs) from a control line (iPSC72.3) and from an affected individual (Family 2, II:1) were cultured in MTeSR media (STEMCELL) in Nunc plates (Fisher) on a matrix of Matrigel (CCHMC PSCF) dissolved in DMEM/F12. One clone of each line was received from the CCHMC PSCF and the Genome Engineering & Stem Cell Center, Department of Genetics, School of Medicine, Washington University in Saint Louis respectively. Cells were passaged every 7 days using Gentle Cell Dissociation Reagent (GCDR, STEMCELL) and fed daily. The STEMdiff™ SMADi Neural Induction Kit (STEMCELL) was used to generate neural rosettes from high-quality iPSC colonies. Briefly, iPSCs were dissociated into single cells and plated into an Aggrewell 800 well at 10,000 cells per well, forming embryoid bodies. These were fed daily, then replated on day 5 by filtering through a 37μM reversible strainer into a 24-well plate containing coverslips coated in Matrigel/DMEM/F12. On day 8, percent neural induction was visually estimated for each well and confirmed to be 75% or above for neural rosettes which were harvested on day 8 or day 11.

Immunocytochemistry

Cells were plated onto coverslips in a 24-well tissue culture plate and fixed in 4% paraformaldehyde for 15 minutes. To permeabilize cell membranes, coverslips were immersed in 0.1% Triton-X 100 for 5 minutes prior to blocking. Coverslips were blocked in 4% NGS for 30 mins before addition of primary antibodi(es) at 4°C overnight. Secondary antibody was applied for 1 hour, then coverslips were co-stained with DAPI for 15 min. They were sealed to glass slides with ProLong Gold Antifade Mountant. Images were acquired on a Nikon C2 Confocal Microscope. Tuj1+ (n=2), PHH3+ (n=3), and CC3+ (n=3) cells were quantified using the Brightspot Detection automated measurement function in NIS-Elements AR software. Three images were captured per coverslip per experiment, with n=2 or n=3 experimental replicates as listed above. The averages of each set of three images are shown in **Fig. 3**. Antibodies and concentrations for immunocytochemistry are listed in **Table S3**.

RNAScope

Wild-type mouse embryos maintained on a CD1 genetic background were dissected at ages E10.5, E14.5, E18.5, P7, and P27 and fixed in formalin for 16-24 h; brains were sub-dissected for ages E18.5-P27. The tissue was washed in PBS, then dehydrated and paraffin embedded by the Cincinnati Children's Hospital Medical Center (CCHMC) Pathology Core. Paraffin blocks were sectioned at 5 um, placed on SuperFrost slides, and baked at 60°C for 1 hour. Target retrieval steps outlined in the manual assay protocol were followed based on recommendations for brain tissue, then slides were dried at room temperature overnight. Hybridization and amplification steps were performed using the HybEZ oven set at 40C. Manual assay protocol from ACDBio was performed using RNAscope Multiplex Fluorescent Reagent Kit V2 (323100), TSA Cyanine 3 Fluorophores (NEL744001KT) at 1:750, and Camsap1 probe made to order by ACDBio (Cat# 866521) (**Fig. S9**).

Mouse husbandry

All animals were maintained through a protocol approved by the Cincinnati Children's Hospital Medical Center IACUC committee (IACUC2019-0068). C57BL/6NJ-*Camsap1*$^{em1(IMPC)J}$ / Mmjax mice (Jackson Labs, MMRRC Stock No. 65662-JAX) were housed in a vivarium with a 12-h light cycle with food and water *ad libitum*. Mice were maintained by intercross from the stock commercially obtained for up to three generations. Mice for dissection were euthanized with isoflurane and cervical dislocation. Whole-brain and skeletal images were taken on a Zeiss Discovery V8 microscope.

Histology

Embryos were dissected, fixed in Bouin's fixative for 48h, washed in 70% ethanol, and dehydrated and paraffin embedded by the CCHMC Pathology Core. Blocks were sectioned by microtome at 10um, then sections were placed on SuperFrost slides, baked >1 hour, and stained with hematoxylin and eosin using standard methods.

Skeletal Preparations

Pups were collected at P0-P1 and frozen. Skin and fat were removed from the embryos prior to fixation in 95% ethanol for 2-5d. The skeletons were stained with Alizarin red and Alcian blue and cleared with potassium hydroxide using standard procedures. Bone measurements were taken with Zen software and assessed for statistical significance with one-way ANOVA (n=6-12 animals per genotype).

Study approvals

Studies were conducted in accordance the declaration of Helsinki. Written informed consent was received from participants prior to inclusion in the study.

- Palestinian Health Research Council - PHRC/HC/518/19
- Cincinnati Children's Hospital Medical Center - 2014-3789
- Baylor College of Medicine - H-29697

Supplemental References

1. Wong, L.J., Naviaux, R.K., Brunetti-Pierri, N., Zhang, Q., Schmitt, E.S., Truong, C., Milone, M., Cohen, B.H., Wical, B., Ganesh, J., et al. (2008). Molecular and clinical genetics of mitochondrial diseases due to POLG mutations. Hum Mutat 29, E150-172.
2. Saneto, R.P., Lee, I.C., Koenig, M.K., Bao, X., Weng, S.W., Naviaux, R.K., and Wong, L.J. (2010). POLG DNA testing as an emerging standard of care before instituting valproic acid therapy for pediatric seizure disorders. Seizure 19, 140-146.
3. Pehlivan, D., Bayram, Y., Gunes, N., Coban Akdemir, Z., Shukla, A., Bierhals, T., Tabakci, B., Sahin, Y., Gezdirici, A., Fatih, J.M., et al. (2019). The Genomics of Arthrogryposis, a Complex Trait: Candidate Genes and Further Evidence for Oligogenic Inheritance. The American Journal of Human Genetics 105, 132-150.
4. Quinodoz, M., Peter, V.G., Bedoni, N., Royer Bertrand, B., Cisarova, K., Salmaninejad, A., Sepahi, N., Rodrigues, R., Piran, M., Mojarrad, M., et al. (2021). AutoMap is a high performance homozygosity mapping tool using next-generation sequencing data. Nature Communications 12, 518.
5. Mitani, T., Isikay, S., Gezdirici, A., Gulec, E.Y., Punetha, J., Fatih, J.M., Herman, I., Akay, G., Du, H., Calame, D.G., et al. (2021). High prevalence of multilocus pathogenic variation in neurodevelopmental disorders in the Turkish population. Am J Hum Genet 108, 1981-2005.
